# Regioselectivity of glycosylation reactions of galactose acceptors: an experimental and theoretical study

**DOI:** 10.3762/bjoc.15.294

**Published:** 2019-12-19

**Authors:** Enrique A Del Vigo, Carlos A Stortz, Carla Marino

**Affiliations:** 1 Universidad de Buenos Aires, Facultad de Ciencias Exactas y Naturales, Consejo Nacional de Investigaciones Científicas y Técnicas, Centro de Investigaciones en Hidratos de Carbono (CIHIDECAR), Departamento de Química Orgánica, Pab. 2, Ciudad Universitaria, 1428 Buenos Aires, Argentina

**Keywords:** Fukui indexes, galactopyranosyl acceptors, galactose donors, molecular modeling, regioselectivity

## Abstract

Regioselective glycosylations allow planning simpler strategies for the synthesis of oligosaccharides, and thus reducing the need of using protecting groups. With the idea of gaining further understanding of such regioselectivity, we analyzed the relative reactivity of the OH-3 and OH-4 groups of 2,6-diprotected methyl α- and β-galactopyranoside derivatives in glycosylation reactions. The glycosyl acceptors were efficiently prepared by simple methodologies, and glycosyl donors with different reactivities were assessed. High regioselectivities were achieved in favor of the 1→3 products due to the equatorial orientation of the OH-3 group. A molecular modeling approach endorsed this general trend of favoring O-3 substitution, although it showed some failures to explain subtler factors governing the difference in regioselectivity between some of the acceptors. However, the Gal*p*-(β1→3)-Gal*p* linkage could be regioselectively installed by using some of the acceptors assayed herein.

## Introduction

Given the importance of carbohydrates in living systems, oligosaccharides and other glycoconjugates are needed to carry out the corresponding glycobiological studies. The heterogeneity of carbohydrates from natural sources makes their isolation difficult, which results in synthesis being the best alternative to obtain the required amounts of carbohydrate-containing molecules. Due to the chemical nature of carbohydrates, with multiple possible linkage positions giving rise to different regioisomers, with two possible anomeric configurations, the chemical synthesis of complex oligosaccharides is difficult and a rather time-consuming effort [1]. Therefore, a carefully designed plan is necessary before starting the synthesis of the desired target structure. Such a plan must include the choice of the glycosylation strategy for the formation of each glycosidic bond, as well as the design of derivatives with temporary protecting groups and one free hydroxy unit in order to achieve glycosylations with respect to the desired regiochemistry. The synthesis of such building blocks is usually the most time-consuming process of oligosaccharide synthesis [2–3].

The knowledge and control of glycosylation regioselectivity of building blocks with more than one free hydroxy group allows reducing the usage of protecting groups, and thus developing simpler reaction sequences for the synthesis of oligosaccharides and glycoconjugates. A current alternative is the use of biocatalysts [4–5], although limited specific enzymes are available. Regioselectivity responds to multiple steric and electronic factors present in both the glycosyl donor and acceptor, and they are characteristic for each particular sugar. Although relative reactivity values have been established for glycosyl donors, it has not been possible to do the same for glycosyl acceptors, whose relative reactivity is still rather poorly understood [6]. Regioselective approaches for the glycosylation of acceptors with more than one free hydroxy group have been developed, and in some of the cases they were successfully rationalized [7–9]. In other cases, the results could not be supported by theoretical studies [10–11].

**Figure 1:**
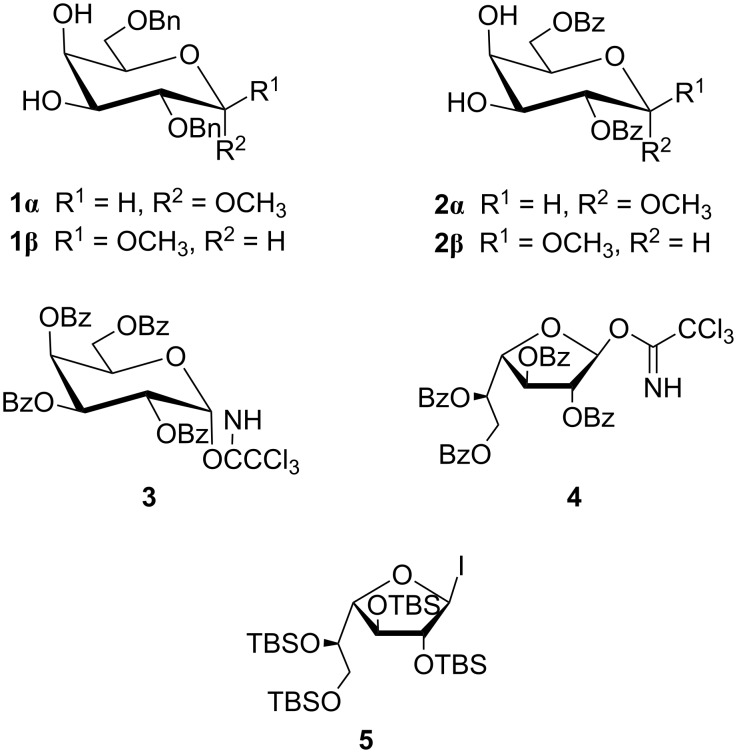
Studied glycosyl acceptors and donors.

ᴅ-Galactose (ᴅ-Gal) is one of the most abundant sugars in nature and a component of oligosaccharides and glycoconjugates with relevant functions [12]. Following a methodology previously applied to ᴅ-glucosamine acceptors [8] with some modifications, in the present study, we evaluated the model of ᴅ-galactose and analyzed the relative reactivity of the OH-3 and OH-4 groups of methyl α- and β-galactose derivatives **1α**/**β** and **2α**/**β** in glycosylation reactions with glycosyl donors **3–5** (Figure 1). We also compared our experimental results with those obtained by a molecular modeling approach.

## Results and Discussion

For this study, ᴅ-Gal*p* derivatives with both their OH-2 and OH-6 group blocked were required. The regioselective functionalization of carbohydrates is usually a difficult task due to the similar reactivity of secondary hydroxy groups [13]. We synthesized derivatives **1α**/**β** and **2α**/**β** in order to compare the differences in the regioselectivity of the glycosylation reaction due to the different electron-withdrawing/-donating properties and anomeric configurations. As donors, **3**–**5** were chosen to assess the effects of the donor's reactivity. The use of acetyl groups was avoided, both in the donors and acceptors, to preclude migration during the glycosylation reactions [14–15].

### Synthesis of the glycosyl acceptors

**Scheme 1:**
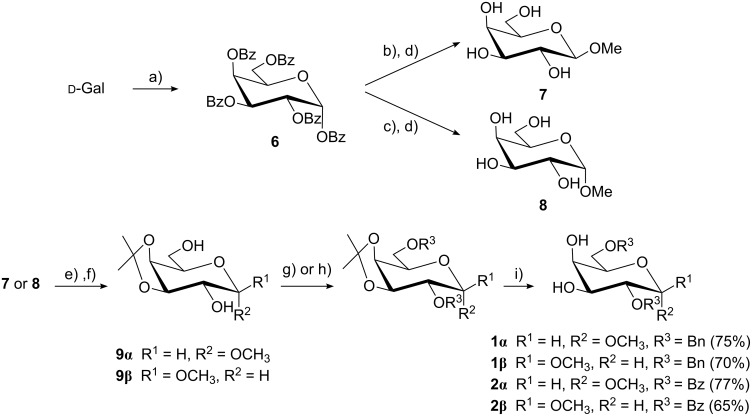
Synthesis of glycosyl acceptors **1α**/**β** and **2α**/**β**. a) BzCl, pyridine, 0 °C, 2 h; b) BF_3_·OEt_2_, MeOH, CH_2_Cl_2_, 4 h; c) SnCl_4_, MeOH, CH_2_Cl_2_, 20 h; d) NaOMe/MeOH, CH_2_Cl_2_, 0 ºC, 2 h; e) (CH_3_)_2_C(OCH_3_)_2_, *p*-TsOH, acetone, rt, 16 h; f) 50% CF_3_COOH, CH_2_Cl_2_, 0 ºC, 15 min; g) BnBr, NaH, THF, rt, 16 h; h) BzCl, pyridine, CH_2_Cl_2_, rt, 12 h; i) AcOH/H_2_O, 4:1, v/v, 65 ºC, 6 h.

The glycosyl acceptors **1α**/**β** and **2α**/**β** were prepared employing protecting group chemistry while trying to simplify the reaction sequences and to optimize the yields. Methyl galactopyranosides **7** and **8** were synthesized from per-*O*-benzoyl-α-ᴅ-Gal*p* (**6**), prepared by benzoylation of galactose in pyridine [16] at low temperature (0 ºC) in order to avoid the formation of furanosic forms, which are usually generated from ᴅ-Gal [17]. The β-anomer **7** was obtained by BF_3_·OEt_2_-promoted glycosylation [18] with a short reaction time, exploiting anchimeric assistance, followed by Zemplén de-*O*-acylation. On the other hand, for the synthesis of the α-anomer **8**, a SnCl_4_-promoted glycosylation was found to be very effective [19], but with a longer reaction time in order to allow for anomerization to occur (Scheme 1) [20].

In our hands, treatment of methyl glycosides **7** and **8** with two equivalents of protecting reagents resulted in the formation of a mixture of di- and trisubstituted derivatives, and thus the regioselectivity could not be controlled. All Gal*p* acceptors were prepared from the corresponding isopropylidene derivatives **9α** or **9β**. For their preparation, methyl glycosides **7** or **8** were treated with 2,2-dimethoxypropane and catalytic amounts of *p*-toluenesulfonic acid, followed by a mild treatment with TFA to hydrolyze the formed byproducts, such as open and mixed acetals [21–22]. Either by benzoylation or benzylation of **9α** or **9β** and subsequent deisopropylidenation, glycosyl acceptors **1α**/**β** and **2α**/**β** were efficiently obtained (Scheme 1). Compounds **1α** and **1β** were previously prepared, but in lower yield [23–24], and compound **2α** was obtained as a byproduct [25].

### Glycosylation reactions

**Scheme 2:**
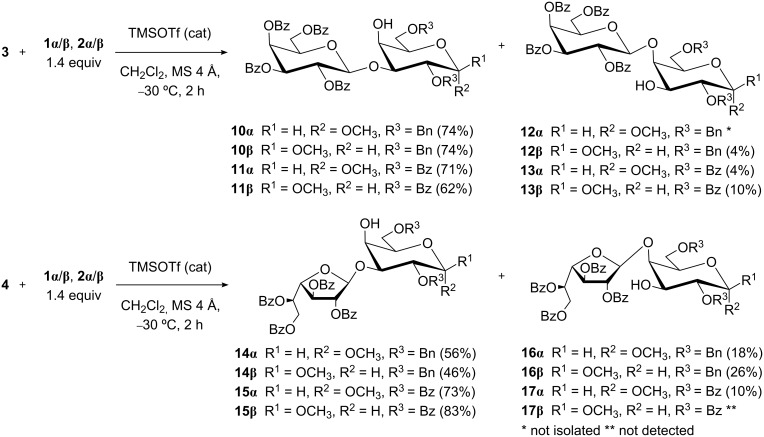
Glycosylation of D-Gal*p* acceptors **1α**/**β** and **2α**/**β** using trichloroacetimidate donors **3** and **4**.

**Scheme 3:**
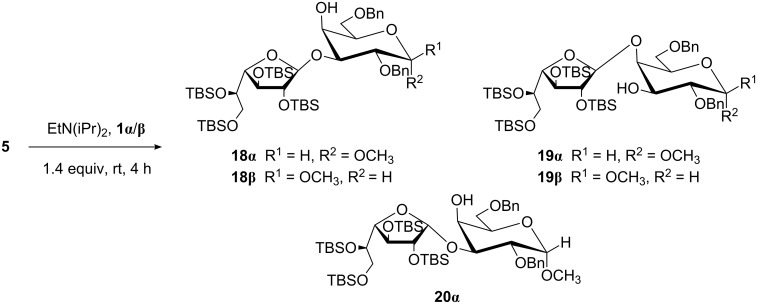
Glycosylation of acceptors **1α**/**β** using galactofuranosyl iodide **5** as donor.

With acceptors **1α**/**β** and **2α**/**β** in hand, we assayed the glycosylation reactions of glycosyl donors **3**–**5**. Trichloroacetimidates **3** [26] and **4** [27] were prepared by treatment of the corresponding benzoylated hemiacetals with trichloroacetonitrile and DBU, as previously described. Glycosylations were performed in CH_2_Cl_2_ and TMSOTf catalysis (Scheme 2). Galactofuranosyl iodide **5** was obtained by the treatment of per-*O*-TBS-β-ᴅ-Gal*f* with a stoichiometric amount of TMSI, and glycosylated in situ by adding the acceptor in the presence of EtN(iPr)_2_ as acid scavenger (Scheme 3) [28]. The acceptor/donor ratio was 1.4:1 to avoid double glycosylation of the acceptors.

All glycosylations (Scheme 2 and Scheme 3) were monitored by TLC, and after the corresponding work-up steps, the crude mixtures were analyzed by ^1^H NMR spectroscopy to establish the ratio of regioisomeric disaccharides and the yield by integration of the signals corresponding to the anomeric positions or other well-resolved signals. The reaction mixtures were purified by column chromatography in order to obtain the products for characterization, and to confirm the yields of the isolated regioisomers. The structures of the disaccharides were univocally assigned on the basis of NMR spectra (see Experimental section, Supporting Information File 1). The position of the interglycosidic linkages was verified from the deshielding of the ^13^C NMR signals involved in such linkages. For example, for disaccharide **10β** (1→3-linked), the main product of the coupling between **3** and **1β**, signals corresponding to C-3 and C-4 were observed at 80.7 and 68.8 ppm, respectively. Instead, for the minor product **12β** (1→4-linked), such signals were observed at 73.5 (C-3) and 76.2 ppm (C-4). A further confirmation was obtained by HMBC analysis, which was particularly useful in the cases in which only one product was detected. For example, for compound **14α**, correlations between signals corresponding to H-1' and C-3 and between C-1' and H-3 were observed. The stereochemistry of the newly formed glycosidic linkages was established from the ^3^*J*_H-1',H-2'_ coupling constants, which were around 8 Hz for disaccharides obtained from pyranosic donor **3** and <0.5 Hz for those obtained from furanosic donors **4** and **5** [29].

For all the acceptors, 1→3 glycosylation products were favored (Table 1, entries 1–10). This trend is in line with the general concept that the equatorial position (OH-3) is more reactive than the axial one (OH-4) due to steric factors [30]. The 1→3 disaccharide **11β** was previously obtained in a similar yield using the same precursors, although the formation of a minor amount of the 1→4 regioisomer **13β** was not reported [31]. The observation that **13β** was formed (Table 1, entry 4) helped to understand the reaction performance and the relative reactivity of hydroxy groups. With the 2,3,4,6-tetra-*O*-benzyl-β-ᴅ-Gal*p* trichloroacetimidate donor, regioselectivity in favor of the OH-3 group of **1α** [32] or allyl 2,6-di-*O*-benzyl-α- or β-ᴅ-Gal*p* was also observed [33].

**Table 1:** Ratios and yields of 1→3 and 1→4 disaccharides obtained by reaction of donors **3**–**5** with acceptors **1α**/**β** and **2α**/**β**.

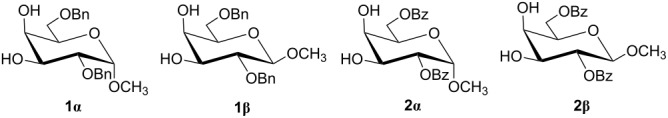							

entry	donor	acceptor	product		ratio^a^ 1→3:1→4	yield (%)^b^	
			1→3	1→4		NMR^a^	isolated^c^

1	**3**	**1α**	**10α**	**12α**	10.3:1	81	74
2	**3**	**1β**	**10β**	**12β**	7:1	81	78
3	**3**	**2α**	**11α**	**13α**	10.8:1	90	75
4	**3**	**2β**	**11β**	**13β**	5.7:1	100	72
5	**4**	**1α**	**14α**	**16α**	3.0:1	79	74
6	**4**	**1β**	**14β**	**16β**	1.8:1	95	72
7	**4**	**2α**	**15α**	**17α**	7.3:1	89	83
8	**4**	**2β**	**15β**	**17β**	1:0	84	83
9	**5**	**1α**	**18α**	**19α**	2.8:1^d^	56	70
10	**5**	**1β**	**18β**	**19β**	2.3:1	47	70

^a^Determined from the ^1^H NMR spectrum of the crude reaction mixture. ^b^Combined yield of the 1→3 and 1→4 regioisomers. ^c^Refers to the isolated pure products after column chromatography on the basis of the donor amount used in the reaction. ^d^**19α** was obtained as an inseparable mixture with **20α**.

For donor **3**, there was no major difference between benzylated (**1α**/**β**) and benzoylated acceptors (**2α**/**β**), and the regioselectivity was higher for the α-anomers (compare Table 1, entries 1 and 2 or 3 and 4, for example). The low nucleophilic character of the OH-4 group in α-anomers could be associated with the lower capacity of the O-5 atom to establish hydrogen bond interactions due to the anomeric effect [34].

For donor **4**, the regioselectivity observed for **1α**, **1β**, and **2α** was lower than that observed for **3**, but for **2β**, the only product detected was the 1→4 disaccharide **15β**. On the other hand, benzoylated acceptors showed higher regioselectivity than benzylated ones. This fact could be attributed to the withdrawing effect of the benzoyl group, which diminished the reactivity of the proximal OH-4 group with respect to the OH-3 moiety [35].

Comparing the different donors, the regioselectivity observed followed the order **3** > **4** > **5** (compare Table 1, entries 1–4 vs 4–8 or 9 and 10), which means that the higher the reactivity of the donor was [35–36], the lower the regioselectivity was, as expected.

Due to the low stereo- and regioselectivities observed for the glycosylation of donor **5** with benzylated Gal*p* acceptors **1α** and **1β** (Scheme 2 and Table 1, entries 9 and 10), its glycosylation reactivity with acceptors **2α**/**β** was not assayed.

### Molecular modeling study

In order to rationalize the observed reactivity of the OH-3/OH-4 groups of acceptors **1α**/**β** and **2α**/**β**, we decided to pursue molecular modeling experiments to determine the atomic partial charges and condensed-to-atom Fukui functions [37]. The former parameter can be used as an estimation of the reactivity: a higher net charge is related to a more facile reaction with a hard electrophile [38]. On the other hand, Fukui functions describe better soft–soft interactions between nucleophiles and electrophiles [8,37–38]. The charge density was calculated for both methods using the Merz–Singh–Kollman scheme (MK) [39–40]. For the calculation of Fukui functions, besides the known computation of differences in atomic charges between the ground-state molecule and the radical cation (*f*_a_) [41], a direct calculation of the frontier molecular orbitals (*f*_b_) [42] was carried out.

**Figure 2:**
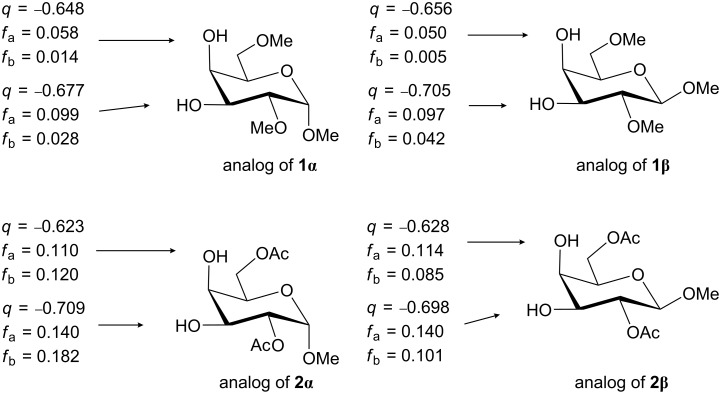
Model Gal*p* 3,4-diol acceptors and data obtained with B3LYP.

For simplicity, analogs of acceptors **1α**/**β** and **2α**/**β**, where benzoyl and benzyl groups were replaced by acetyl and methyl moieties, respectively, were used (Figure 2). After a full conformational search with MM3, the lower-energy structures were submitted to optimization with B3LYP/6-311+G**, and then, single-point calculations with M06-2X/6-311+G** (Figure S1 and Table S1, Supporting Information File 1). After calculations for each low-energy conformer and Boltzmann-averaging, the local charges and Fukui functions corresponding to each compound were generated (Table S2 and Table S3, Supporting Information File 1).

The higher reactivity of the O-3 atom with respect to position O-4 that was experimentally observed was also predicted by modeling. Figure 2 shows the data obtained with the B3LYP functional for the OH-3 and OH-4 groups, and Table 2 shows the difference in the charge of atoms O-3/O-4 (*q*) and Fukui functions (*f*). These differences are all positive for Fukui functions, whereas they are negative for charge determinations, indicating that for all acceptors, calculations predict that the OH-3 moiety is more nucleophilic, having higher negative charges *q* and Fukui coefficients *f* than the OH-4 function. In the case of acylated acceptors (analogs of **2α**/**β**), the system predicted the lower selectivity of the β-anomer, using either charges or Fukui coefficients (Table 2). Nevertheless, the change in selectivity predicted for the benzylated diol analogs of **1α**/**1β** did not match the experimental trend.

**Table 2:** Differences of charges and Fukui functions of the O-3/O-4 positions for analogs of acceptors **1α**/**β** and **2α**/**β**.

	B3LYP calculations			M06-2X calculations		
	*q*_O-3_ − *q*_O-4_	*f*_aO-3_ − *f*_aO-4_	*f*_bO-3_ − *f*_bO-4_	*q*_O-3_ − *q*_O-4_	*f*_aO-3_ − *f*_aO-4_	*f*_bO-3_ − *f*_bO-4_

analog of **1α**	−0.029	0.041	0.014	−0.028	0.042	0.035
analog of **1β**	−0.049	0.047	0.037	−0.047	0.039	0.040
analog of **2α**	−0.086	0.030	0.062	−0.084	0.025	0.084
analog of **2β**	−0.070	0.026	0.016	−0.078	0.049	0.030

Similar results were observed with the M06-2X functional (Table 2). The calculations gave a good prediction of the higher OH-3 group’s reactivity, but an accurate prediction of the trends in selectivity could not be achieved.

We have tried to explain the reduced regioselectivity of the β-anomers through hydrogen bonding interactions of the OH-3 and OH-4 groups of the model acceptors. Doutheau and co-workers proposed that such a reduced regioselectivity could be ascribed to the greater basicity of the O-ring of the β-anomers [34], which results in a stronger hydrogen bond OH-4⋅⋅⋅O-5. Although stronger interactions were observed for some of the conformers (Table S1, Supporting Information File 1), they corresponded to the less stable conformers.

## Conclusion

Simple procedures for the synthesis of acceptors **1α** and **2β** were optimized. Experimentally, a greater reactivity of the OH-3 group was observed for the acceptors **1α**/**β** and **2α**/**β**, in agreement to what is expected for equatorial hydroxy groups. Donor **3** reacted with more regioselectivity than **4** and **5**, in accordance with its lower reactivity.

Computational results have set out the predicted increase in reactivity of the OH-3 moiety compared to that of the OH-4 function by using either electron density or Fukui functions, but have failed to agree with the subtle factors governing the differences in regioselectivity between some of the acceptors.

The high regioselectivity achieved for the glycosylation of pyranosyl donor **3** with acceptors **1α** and **2α** indicates that they are good precursors to be taken into account when planning the synthesis of molecules containing the Gal*p*-(β1→3)-Gal*p* motif.

## Supporting Information

File 1 Additional figures and tables, full synthetic details, and ^1^H and ^13^C NMR spectra for compounds **1**, **2**, and **10**–**19**.
