# Supplementary material for: Regioselectivity of glycosylation reactions of galactose acceptors: an experimental and theoretical study
Source: Beilstein J Org Chem. 2019 Dec 19;15:2982–9. doi: 10.3762/bjoc.15.294 (PMC6941450; doi:10.3762/bjoc.15.294)
Supplement: File 1 — Additional figures and tables, full synthetic details, and 1H and 13C NMR spectra for compounds 1, 2, and 10–19. [file Beilstein_J_Org_Chem-15-2982-s001.pdf]

BEILSTEIN JOURNAL OF ORGANIC CHEMISTRY

Supporting Information

for

Regioselectivity of glycosylation reactions of galactose acceptors: an experimental and theoretical study

Enrique A. Del Vigo, Carlos A. Stortz and Carla Marino

Beilstein J. Org. Chem. **2019,** *15,* 2982–2989. doi:10.3762/bjoc.15.294

Additional figures and tables, full synthetic details, and ^{1}H and ^{13}C NMR spectra for compounds 1, 2, and 10–19

License and Terms: This is a supporting information file under the terms of the Creative Commons Attribution License (https://creativecommons.org/licenses/by/4.0). Please note that the reuse, redistribution and reproduction in particular requires that the authors and source are credited.
The license is subject to the *Beilstein Journal of Organic Chemistry* terms and conditions: (https://www.beilstein-journals.org/bjoc)

Table of contents **page**

Figure S1 .... S2
Table S1 .... S3
Table S2 .... S6
Table S3 .... S7
Experimental S12
Computational methods S31
References S32
^{1}H and ^{13}C NMR spectra S33

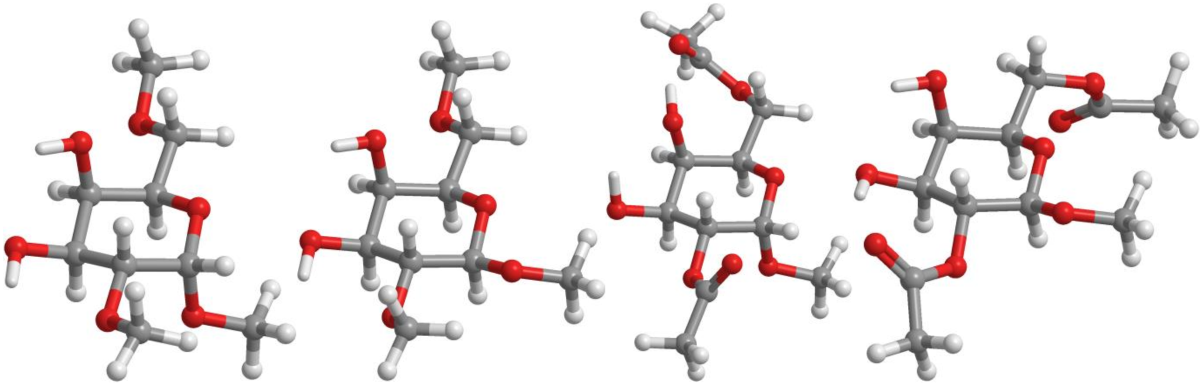

1α **1β** **2α** **2β**

Figure S1: Minimal-energy conformers of analogs of acceptors **1**–**2**.

Table S1: Relative energies and geometries of the conformers found by B3LYP/6-311+G** on analogues of compounds **1α**–**2β**.

								hydrogen bonds					
conformer	ΔE	χ_1[a]	χ_2[a]	χ_3[a]	χ_4[a]	ω[a]	χ_5[a]		d(O··H)/Å	Θ/°[b]		d(O··H)/Å	Θ/°[b]
analog of **1α**													
1	0.00	−47	−48	72	73	169	174	HO3···O2	2.32	109	HO4···O3	2.24	111
2	0.14	−50	−49	73	75	75	−175	HO3···O2	2.32	109	HO4···O3	2.22	112
3	1.00	−49	−49	72	74	69	-97	HO3···O2	2.33	109	HO4···O3	2.22	112
4	1.10	−41	35	−161	−87	−72	177	HO3···O4	2.13	114	HO4···O6	1.88	143
5	1.27	−41	34	−160	−83	−70	92	HO3···O4	2.13	114	HO4···O6	1.90	144
6	1.87	−47	−48	73	72	169	−86	HO3···O2	2.32	109	HO4···O3	2.25	110
7	2.06	−47	−49	73	75	71	83	HO3···O2	2.32	109	HO4···O3	2.22	112
8	2.16	−42	35	−157	−44	−166	−173	HO3···O4	2.12	116	HO4···O6	2.41	118
9	2.32	−43	−29	−162	−86	−72	177	HO3···O4	2.15	113	HO4···O6	1.88	143
10	2.35	−44	156	−168	−87	−72	176	HO3···O4	2.24	109	HO4···O6	1.89	143
11	2.46	−49	175	92	73	170	175	HO3···O2	2.34	113	HO4···O3	2.24	111
12	2.48	−44	156	−166	−83	−70	92	HO3···O4	2.23	110	HO4···O6	1.91	144
13	2.63	−51	178	91	75	75	−175	HO3···O2	2.33	114	HO4···O3	2.21	112
14	2.69	−42	37	−156	−160	173	177	HO3···O4	2.21	115	HO4···O5	2.46	109
analog of **1β**													
1	0.00	52	−34	71	74	171	175	HO3···O2	2.35	108	HO4···O3	2.26	110
2	0.34	52	−35	71	76	75	−174	HO3···O2	2.34	109	HO4···O3	2.23	112
3	1.31	50	−34	71	75	70	−97	HO3···O2	2.35	108	HO4···O3	2.24	111
4	1.85	49	−17	−162	−85	−73	177	HO3···O4	2.16	113	HO4···O6	1.89	142
5	1.88	47	−16	−160	−81	−70	91	HO3···O4	2.15	113	HO4···O6	1.92	143
6	2.00	45	20	−159	−81	−69	90	HO3···O4	2.16	113	HO4···O6	1.92	143
7	2.02	52	−34	71	73	169	−87	HO3···O2	2.35	108	HO4···O3	2.27	110
8	2.05	47	20	−161	−85	−73	176	HO3···O4	2.16	113	HO4···O6	1.89	142
9	2.08	48	−17	−156	−161	173	177	HO3···O4	2.23	115	HO4···O5	2.37	110
10	2.15	46	26	−156	−161	174	176	HO3···O4	2.24	115	HO4···O5	2.36	110

11	2.22	53	−35	72	76	69	81	HO3···O2	2.34	109	HO4···O3	2.23	111
12	2.61	48	−19	−157	−161	75	−175	HO3···O4	2.23	114	HO4···O5	2.37	110
13	2.78	46	26	−157	−161	76	−175	HO3···O4	2.23	114	HO4···O5	2.36	110
14	2.84	49	−19	−158	−42	−165	−174	HO3···O4	2.14	115	HO4···O6	2.51	115
analog of **2α**													
1	0.00	−44	−34	-160	-44	169	97	HO3···O4	2.15	114	HO4···O=C	1.95	152
2	1.21	−47	40	106	73	69	85	HO3···O=C	1.85	158	HO4···O3	2.15	113
3	1.26	−48	40	106	73	72	−179	HO3···O=C	1.85	158	HO4···O3	2.15	113
4	1.40	−48	40	105	72	172	−87	HO3···O=C	1.85	159	HO4···O3	2.16	113
5	1.45	−46	40	107	72	172	−178	HO3···O=C	1.86	158	HO4···O3	2.17	112
6	1.49	−46	−45	66	72	69	84	HO3···O2	2.44	104	HO4···O3	2.24	110
7	1.58	−48	−45	66	72	173	−87	HO3···O2	2.43	105	HO4···O3	2.25	110
8	1.62	−46	−46	66	72	172	−178	HO3···O2	2.43	104	HO4···O3	2.25	110
9	1.63	−48	−45	66	73	72	180	HO3···O2	2.44	104	HO4···O3	2.24	110
10	2.63	−43	6	−167	−89	−70	165	HO3···O4	2.22	110	HO4···O6	1.95	142
11	2.64	−44	−34	−164	−89	−70	166	HO3···O4	2.17	112	HO4···O6	1.95	142
12	2.81	−44	−33	−158	−160	174	180	HO3···O4	2.23	114	HO4···O5	2.46	108
13	2.90	−46	−33	−158	−161	72	−176	HO3···O4	2.23	114	HO4···O5	2.46	108
14	2.94	−46	−33	−158	−161	174	-88	HO3···O4	2.24	114	HO4···O5	2.43	108
analog of **2β**													
1	0.00	52	39	98	76	68	83	HO3···O=C	1.87	157	HO4···O3	2.13	114
2	0.08	49	9	−166	−43	170	97	HO3···O4	2.24	110	HO4···O=C	1.97	151
3	0.42	51	39	98	75	176	−86	HO3···O=C	1.87	157	HO4···O3	2.15	113
4	0.46	52	38	98	76	72	179	HO3···O=C	1.87	157	HO4···O3	2.14	114
5	0.51	51	39	98	76	173	−178	HO3···O=C	1.87	157	HO4···O3	2.14	113
6	0.93	177	−6	−162	−44	170	97	HO3···O4	2.20	112	HO4···O=C	1.96	151
7	1.74	47	−177	−162	−43	170	97	HO3···O4	2.18	113	HO4···O=C	1.95	152
8	1.91	48	6	−164	−162	175	179	HO3···O4	2.33	110	HO4···O5	2.42	108
9	1.96	48	7	−164	−163	67	83	HO3···O4	2.33	110	HO4···O5	2.42	108

10	1.99	47	6	−164	−162	177	−87	HO3···O4	2.34	110	HO4···O5	2.40	109
11	2.16	48	7	−164	−162	73	−177	HO3···O4	2.34	110	HO4···O5	2.42	108
12	2.45	52	−10	69	75	68	83	HO3···O2	2.55	104	HO4···O3	2.22	111
13	2.46	171	38	98	75	65	81	HO3···O=C	1.88	157	HO4···O3	2.14	113
14	2.59	49	10	−170	−90	−69	165	HO3···O4	2.27	107	HO4···O6	1.96	142
15	2.61	49	10	−170	−89	−66	130	HO3···O4	2.27	108	HO4···O6	1.97	142
16	2.74	51	−10	69	74	175	-86	HO3···O2	2.54	104	HO4···O3	2.24	110
17	2.77	174	37	99	73	173	180	HO3···O=C	1.88	157	HO4···O3	2.17	112
18	2.80	51	−10	69	74	173	−178	HO3···O2	2.54	104	HO4···O3	2.24	110
19	2.84	173	37	99	74	72	−175	HO3···O=C	1.88	157	HO4···O3	2.16	113
20	2.85	52	−10	69	75	72	180	HO3···O2	2.54	104	HO4···O3	2.23	111

[a]Dihedral angle χ_n, defined by the atoms H-*n*–C-*n*–O-n-H(O)-*n*, with *n* = 1 to 4. Angle χ_6 defined by the atoms C-5–C-6–O-6–(O) and ω by the atoms O-5-C-5-C-6-O-6. [b]Angle Ɵ defined by the atoms O–H··O.

Table S2: Charges and Fukui functions of O-3 and O-4 observed for analogs of acceptors **1** and **2**.

	B3LYP calculations						M06-2X calculations					
	$q_{O\text{-}3}$	$q_{O\text{-}4}$	$f_{aO\text{-}3}$	$f_{aO\text{-}4}$	$f_{bO\text{-}3}$	$f_{bO\text{-}4}$	$q_{O\text{-}3}$	$q_{O\text{-}4}$	$f_{aO\text{-}3}$	$f_{aO\text{-}4}$	$f_{bO\text{-}3}$	$f_{bO\text{-}4}$
analog of **1α**	−0.677	−0.648	0.099	0.058	0.028	0.014	−0.673	−0.645	0.097	0.056	0.056	0.021
analog of **1β**	−0.705	−0.656	0.097	0.050	0.042	0.005	−0.699	−0.652	0.096	0.058	0.047	0.006
analog of **2α**	−0.709	−0.623	0.140	0.110	0.182	0.120	−0.711	−0.627	0.142	0.116	0.163	0.079
analog of **2β**	−0.698	−0.628	0.140	0.114	0.101	0.085	−0.698	−0.620	0.134	0.085	0.088	0.058

Table S3: Coordinates of minimal energy conformers.

analog of **1α**	atom	x	y	z		atom	x	y	z
conformer 1	C	1.121948	0.991624	0.468310	conformer 2	C	0.710192	1.051327	0.424911
	C	1.601436	−0.458620	0.267077		C	1.666045	−0.152652	0.322066
	C	0.727246	−1.160270	−0.768905		C	1.182602	−1.112973	−0.760853
	C	−0.744085	−1.098553	−0.351530		C	−0.264787	−1.539768	−0.501040
	C	−1.136448	0.354044	−0.069598		C	−1.142088	−0.302762	−0.293153
	C	−2.526062	0.495401	0.536495		C	−2.552180	−0.652498	0.144794
	O	−0.239820	0.991058	0.857505		O	−0.596209	0.583404	0.698748
	H	1.636084	1.468402	1.312046		H	0.952293	1.679862	1.291141
	O	1.357137	1.712343	−0.710243		O	0.793560	1.801300	−0.755707
	O	2.943191	−0.588252	−0.179004		O	3.011675	0.166888	0.001142
	H	1.481383	−0.965967	1.233324		H	1.628534	−0.664074	1.293285
	H	0.853187	−0.658550	−1.734928		H	1.240808	−0.605166	−1.730148
	O	1.079463	−2.538930	−0.882539		O	1.973628	−2.301022	−0.780628
	H	−1.367254	−1.459952	−1.178601		H	−0.632270	−2.073853	−1.389264
	O	−0.970757	−1.889749	0.810104		O	−0.351882	−2.385142	0.639468
	H	−1.115428	0.892577	−1.024268		H	−1.203142	0.226240	−1.251850
	H	−2.679470	1.542725	0.831606		H	−2.549397	−0.916082	1.209044
	H	−2.608191	−0.137291	1.427611		H	−2.905558	−1.526023	−0.423137
	O	−3.468333	0.107250	−0.448775		O	−3.392011	0.463616	−0.100334
	C	−4.795135	0.077048	0.040349		C	−4.706394	0.276720	0.384845
	C	3.933103	−0.277689	0.789115		C	3.738918	0.817585	1.031592
	C	1.172157	3.118412	−0.580634		C	0.083993	3.037822	−0.705684
	H	2.040086	−2.578870	−0.964549		H	2.897851	−2.024275	−0.759070
	H	−0.544640	−2.740847	0.648411		H	0.366331	−3.026506	0.562416
	H	−5.115951	1.068001	0.391779		H	−4.714889	0.126337	1.473258
	H	−4.899920	−0.640410	0.865353		H	−5.193991	−0.585984	−0.091962
	H	−5.436490	−0.231492	−0.785683		H	−5.270180	1.178980	0.145738
	H	3.772244	−0.841622	1.717586		H	3.681209	0.256752	1.974004

	H	3.956659	0.793535	1.020660		H	3.381259	1.839766	1.200027
	H	4.894055	−0.561611	0.359649		H	4.777858	0.861861	0.704334
	H	1.825455	3.526841	0.201282		H	0.459569	3.667521	0.111260
	H	0.133862	3.367624	−0.341873		H	−0.988953	2.877330	−0.568979
	H	1.442312	3.558768	−1.539856		H	0.264778	3.538164	−1.656491
analog of **1β**									
conformer 1	C	1.093138	0.940922	−0.280665	conformer 2	C	0.689269	0.997085	−0.267584
	C	1.533046	−0.491856	0.044522		C	1.627738	−0.173015	0.052007
	C	0.527392	−1.472214	−0.564361		C	1.070213	−1.441776	−0.597709
	C	−0.901326	−1.172162	−0.108446		C	−0.381333	−1.707659	−0.192323
	C	−1.209800	0.306785	−0.366305		C	−1.214690	−0.438665	−0.408327
	C	−2.551511	0.748930	0.202603		C	−2.625990	−0.570834	0.135332
	O	−0.223033	1.159770	0.215114		O	−0.610398	0.692075	0.218274
	H	1.096434	1.099377	−1.375743		H	0.640838	1.160653	−1.360899
	O	1.952525	1.838938	0.336648		O	1.142473	2.143701	0.369606
	O	2.796794	−0.837186	−0.510791		O	2.942037	−0.021890	−0.471046
	H	1.545763	−0.602127	1.135122		H	1.656219	−0.292218	1.141750
	H	0.575166	−1.375640	−1.658544		H	1.109752	−1.309381	−1.688466
	O	0.829516	−2.813090	−0.189830		O	1.832919	−2.586838	−0.227965
	H	−1.600601	−1.772948	−0.702867		H	−0.781407	−2.498382	−0.843824
	O	−1.063766	−1.457873	1.274430		O	−0.470978	−2.109615	1.166981
	H	−1.231148	0.455544	−1.457091		H	−1.282780	−0.261909	−1.493243
	H	−2.646990	1.836908	0.082332		H	−2.602494	−0.470002	1.226768
	H	−2.596818	0.506764	1.270503		H	−3.020576	−1.568576	−0.107994
	O	−3.567974	0.071431	−0.515952		O	−3.434048	0.434049	−0.452644
	C	−4.862139	0.306486	0.005045		C	−4.739133	0.473741	0.089561
	C	3.934023	−0.525710	0.293830		C	3.860270	0.691078	0.357874
	C	1.737562	3.203027	−0.023337		C	0.422311	3.327993	0.023712
	H	1.771037	−2.945044	−0.356029		H	2.761985	−2.356971	−0.353264
	H	−0.677565	−2.329605	1.424652		H	0.234377	−2.752570	1.314701
	H	−5.124829	1.372906	−0.038718		H	−4.719695	0.693043	1.165979

	H	−4.940858	-0.033782	1.046272		H	−5.269601	−0.477034	−0.064873
	H	−5.564161	-0.257520	-0.609632		H	−5.278606	1.268491	−0.426452
	H	3.857934	-1.006464	1.277305		H	3.944856	0.214655	1.342811
	H	4.044391	0.551762	0.429566		H	3.555174	1.730872	0.488014
	H	4.800788	-0.924273	-0.234388		H	4.825799	0.646389	−0.146901
	H	0.759357	3.550037	0.317133		H	−0.622070	3.256573	0.334320
	H	1.815068	3.334033	-1.110201		H	0.470774	3.507776	−1.057744
	H	2.522214	3.778903	0.466060		H	0.909485	4.149057	0.548401
analog of 2α									
conformer 1	C	1.248542	1.102030	0.390514	conformer 2	C	−0.540965	1.034945	−0.640177
	C	1.550876	−0.380479	0.117579		C	−1.648881	0.017498	−0.323821
	C	0.524938	−0.995416	−0.838550		C	−1.200677	−0.974200	0.752264
	C	−0.895108	−0.742377	−0.303674		C	0.163416	−1.588790	0.361407
	C	−1.097432	0.744936	0.030495		C	1.172103	−0.518889	−0.052367
	C	−2.388984	1.059277	0.784129		C	2.431500	−1.136200	−0.639691
	O	−0.071400	1.240957	0.896805		O	0.627492	0.364886	−1.049223
	H	1.890558	1.483278	1.189902		H	-0.834117	1.648332	−1.500428
	O	1.441271	1.826362	−0.793818		O	−0.340500	1.839469	0.490633
	O	2.846318	−0.541199	−0.484684		O	−2.805506	0.812378	0.062353
	H	1.533222	−0.900124	1.076467		H	−1.887750	−0.520886	−1.243226
	H	0.636813	−0.524802	−1.817637		H	−1.094620	−0.436997	1.701059
	O	0.738227	−2.381703	−1.026056		O	−2.087158	−2.077562	0.918001
	H	−1.604468	−1.027117	−1.091297		H	0.558220	−2.104745	1.247836
	O	−1.052372	−1.593398	0.825999		O	−0.001649	−2.510143	−0.706088
	H	−1.090635	1.306490	−0.912690		H	1.432406	0.058638	0.840050
	H	−2.393908	2.114569	1.050612		H	2.234480	−1.503491	−1.643588
	H	−2.471364	0.454738	1.686098		H	2.776433	−1.950335	−0.002369
	O	−3.557946	0.873280	−0.050696		O	3.490748	−0.163598	−0.788530
	C	−4.231721	-0.290030	0.024534		C	4.268386	0.071761	0.291625
	C	3.920781	-0.585454	0.334481		C	−4.039148	0.297491	−0.067985
	C	1.500204	3.238139	−0.610643		C	0.445877	3.001651	0.240784

	H	0.338879	−2.823062	−0.263121		H	−2.941092	−1.875396	0.500829
	H	−2.002229	−1.700197	0.991340		H	−0.768212	−3.050986	−0.469431
	O	−3.870245	−1.239142	0.687182		O	4.097741	−0.450219	1.367508
	C	−5.489453	−0.248121	−0.799128		C	5.369952	1.045788	−0.039810
	C	5.175628	−0.864674	−0.450562		C	−5.097297	1.346074	0.136039
	O	3.859756	−0.425831	1.530020		O	−4.260220	−0.868294	−0.324154
	H	2.308088	3.506772	0.080773		H	−0.037369	3.642364	−0.507375
	H	0.554909	3.633917	−0.225832		H	1.449224	2.738625	−0.107063
	H	1.704797	3.671736	−1.589006		H	0.514485	3.540092	1.185162
	H	−5.292397	0.188974	−1.778992		H	4.977360	1.896223	−0.599387
	H	−6.223716	0.387270	−0.296327		H	6.109676	0.549093	−0.673554
	H	−5.892977	−1.253228	−0.900755		H	5.849526	1.379320	0.878265
	H	5.266649	-0.160412	-1.279786		H	−5.139028	1.987635	−0.748639
	H	5.119027	−1.868575	−0.878661		H	−4.849084	1.977355	0.990170
analog of **2β**									
conformer 1	C	−0.479602	1.062040	−0.134760	conformer 2	C	1.141280	1.297388	−0.261307
	C	−1.593053	0.007675	−0.147397		C	1.525885	−0.160479	−0.004964
	C	−1.125737	−1.324315	0.455112		C	0.500719	−1.109689	−0.642613
	C	0.257495	−1.750178	−0.078268		C	−0.920553	−0.758800	−0.178882
	C	1.236498	−0.575879	−0.052146		C	−1.186118	0.743801	−0.391902
	C	2.556134	−0.930387	−0.720366		C	−2.491771	1.252117	0.218467
	O	0.691205	0.549623	−0.739519		O	−0.181299	1.542254	0.222058
	H	−0.251994	1.353140	0.908195		H	1.163257	1.514765	−1.345940
	O	−0.920949	2.154924	−0.861244		O	2.018326	2.119401	0.417242
	O	−2.666266	0.594952	0.643410		O	2.799594	−0.394816	−0.625767
	H	−1.936853	-0.125361	−1.174552		H	1.601383	−0.325318	1.069913
	H	−1.047938	−1.190008	1.543579		H	0.558753	−0.991944	−1.729718
	O	−2.003937	−2.405028	0.164907		O	0.802220	−2.465532	−0.384746
	H	0.638985	−2.536385	0.589120		H	−1.625555	−1.334855	−0.792170
	O	0.155561	−2.239774	−1.403454		O	−1.010960	−1.153702	1.180963
	H	1.424571	−0.314636	0.999170		H	−1.208196	0.932872	−1.478099

H	2.420743	−0.986485	−1.797465	H	−2.549235	2.330686	0.083742
H	2.923003	−1.882583	−0.336423	H	−2.540319	1.012345	1.279666
O	3.558791	0.088792	−0.513037	O	−3.649834	0.720669	−0.468763
C	4.242128	0.054888	0.652521	C	−4.267151	−0.362835	0.042408
C	−3.932744	0.200274	0.430874	C	3.792491	−0.945417	0.124070
C	−0.064131	3.293980	−0.790748	C	1.914985	3.502846	0.089639
H	−2.886855	−2.055385	−0.044513	H	0.537683	−2.640831	0.528966
H	−0.633887	−2.798988	−1.419976	H	−1.949489	−1.241326	1.407481
O	4.034801	−0.752793	1.526862	O	−3.857558	−0.984748	0.998717
C	5.286333	1.140459	0.695871	C	−5.528482	−0.680158	−0.712791
C	−4.916706	1.110601	1.110197	C	5.025177	−1.150388	−0.717377
O	−4.230144	−0.769821	−0.235276	O	3.683194	−1.228839	1.288184
H	0.908805	3.083104	−1.240389	H	0.950858	3.912572	0.401714
H	0.072670	3.612287	0.250659	H	2.048172	3.657965	−0.988753
H	−0.562677	4.086810	−1.346704	H	2.717005	4.006656	0.627362
H	6.056005	0.936090	−0.052759	H	−5.358074	−0.621314	−1.788670
H	5.738401	1.171554	1.685025	H	−6.291390	0.060970	−0.458942
H	4.840330	2.106126	0.450545	H	−5.882052	−1.670205	−0.433137
H	−4.980585	2.044523	0.544918	H	5.301951	−0.220083	−1.217204
H	−4.579203	1.357354	2.117726	H	4.816044	−1.893339	−1.491081

Experimental

General Procedures

The solvents used were distilled, dried, and stored according to standard procedures. Analytical thin-layer chromatography (TLC) was performed on Silica Gel 60 F254 (Merck) aluminium supported plates (layer thickness 0.2 mm). Visualization of the spots was effected by exposure to UV light and charring with a solution of 5% (v/v) sulfuric acid in EtOH containing 0.5% *p*-anisaldehyde. Column chromatography was carried out with Silica Gel 60 (230–400 mesh, Merck). Optical rotations were measured with a Perkin-Elmer 343 digital polarimeter at 25 ºC. NMR spectra were recorded with a Bruker AMX 500 instrument. Chemical shifts (δ) are reported in ppm, with residual chloroform (δ 7.26 for ^{1}H and δ 77.16 for ^{13}C) as internal references. Assignments of ^{1}H and ^{13}C NMR spectra were assisted by 2D ^{1}H COSY and HSQC experiments. High-resolution mass spectra (HRMS) were obtained by Electrospray Ionization (ESI) and Q-TOF detection.

Glycosyl acceptors

Methyl 2,6-di-*O*-benzyl-α-D-galactopyranoside (1α)

To a suspension of methyl α-D-galactopyranoside (**8**, 0.15 g, 0.78 mmol) in anhydrous acetone (7.0 mL) 2,2-dimethoxypropane (1.1 mL, 8.8 mmol) and *p*-toluenesulfonic acid (5.0 mg, 0.03 mmol) were added and the mixture was stirred at room temperature for 16 h. Then, the reaction mixture was neutralized with Et_3N (15 µl, 0,11 mmol), and the mixture was concentrated and codistillated with toluene under reduced pressure (10.0 mL). The residue was dissolved in CH_2Cl_2 (1.5 mL) and treated with 50% TFA (22 µL, 0.14 mmol) at 0 °C for 15 min. Then, Et_3N (33 µL, 0.26 mmol) was added, and the solvent was evaporated under reduced pressure to afford **9α**.

Dried compound **9α** was dissolved in anhydrous THF (5.0 mL) and treated with a suspension of 60% NaH in mineral oil (0.15 g, 3.75 mmol), previously washed twice with hexane. The suspension was cooled to 0 °C, and BnBr (0.04 g, 3.36 mmol) was added. The mixture was stirred at room temperature for 16 h. After cooling to 0 ºC, the reaction was quenched by addition MeOH/H_2O, 1:1, v/v (1.0 mL) and stirred for 0.5 h. The solvent was evaporated under reduced pressure and the residue was dissolved in 80% HAcO and stirred at 65 °C for 15 h. The mixture was concentrated under reduced pressure, diluted with CH_2Cl_2 (20 mL), and washed successively with water, saturated $NaHCO_3$, and water, dried (Na_2SO_4) and concentrated under reduced pressure. The crude mixture was purified by silica gel column chromatography (65:35, hexane/EtOAc, v/v) to afford **1α** (0.22 g, 75%) as a white solid. R_f 0.45 (1:1 hexane/AcOEt), $[\alpha]_D$ +77 (*c* 1, $CHCl_3$); lit: $[\alpha]_D^{22}$ +74.9 (c 1.68, $CHCl_3$) [1]. ^{1}H NMR (500 MHz, $CDCl_3$): δ 7.39-7.27 (m, 10H, aromatic), 4.74 (d, 1H, $J_{1,2}$ = 3.7 Hz, H-1), 4.72, 4.68 (2d, 2H, J_{gem} = 12.1 Hz, CH_2Ph), 4.60 (s, 2H, CH_2Ph), 4.09 (d, 1H, $J_{3,4}$ = 3.3 Hz, H-4), 3.99 (dd, 1H, $J_{3,4}$ = 3.3 Hz, $J_{2,3}$ = 9.8 Hz, H-3), 3.94 (t, 1H, $J_{5,6a}$ = $J_{5,6a}$ = 5.6 Hz, H-5), 3.78 (m, 3H, H-6a, H-2, H-6b), 3.38 (s, 3H, CH_3O),^{13}C NMR (126 MHz, $CDCl_3$): δ 138.0, 137.7, 128.5, 128.4, 128.07, 128.02, 127.7, 127.6 (aromatic), 97.9 (C-1), 76.6 (C-2), 73.6 (*C*H_2Ph), 72.9 (*C*H_2Ph), 69.9, 69.7 (C-3, C- 4), 69.3 (C-6), 68.2 (C-5), 55.3 (*C*H_3O).

Methyl 2,6-di-*O*-benzyl-β-D-galactopyranoside (1β)

Obtained from methyl β-D-Gal*p* (**8**, 0.1 g, 0,52 mmol) with the same procedure described for **1α**. After purification by column chromatography (65:35, hexane/AcOEt, v/v), compound **1β** (0.13 g, 70%) was obtained as as a white crystalline solid. R_f 0.40 (1:1, hexane/AcOEt, v/v), $[\alpha]_D$ +9 (*c* 1, $CHCl_3$); mp 84-84; lit: $[\alpha]_D^{25}$ +8.6 (*c* 1.08, $CHCl_3$); mp 83-85 ºC [2]. ^1H NMR (500 MHz, $CDCl_3$): δ 7.39-7.27 (m, 10H, aromatic), 4.94 (d, 1H, J_{gem} = 11.5 Hz, CH_2Ph), 4.66 (d, 1H, J_{gem} = 11.5 Hz, CH_2Ph), 4.59 (s, 2H, CH_2Ph), 4.28 (d, 1H, $J_{1,2}$ = 7.7 Hz, H-1), 3.98 (dd, 1H, $J_{4,5}$ = 0.9 Hz,$J_{3,4}$ = 3.4 Hz, H-4), 3.80 (dd, 1H, $J_{5,6a}$ = 5.6 Hz, J_{gem} = 10.1 Hz, H-6a), 3.75 (dd, 1H, $J_{5,6b}$ = 5.7 Hz, J_{gem} = 10.1 Hz, H-6b), 3.61 (ddd, 1H,$J_{4,5}$ = 0.9 Hz, $J_{5,6a}$ = 5.6 Hz, $J_{5,6b}$ = 5.7 Hz, H-5), 3.59 (dd, 1H, $J_{3,4}$ = 3.4 Hz, $J_{2,3}$ = 9.4 Hz, H-3), 3.57 (s, 3H, CH_3O), 3.49 (dd, 1H, $J_{1,2}$ = 7.7 Hz, $J_{2,3}$ = 9.4 Hz, H-2), ^{13}C NMR (126 MHz, $CDCl_3$): δ 138.4, 137.8, 128.5, 128.4, 128.0, 127.8, 127.78, 127.72 (aromatic), 104.6 (C-1), 79.1 (C-2), 74.5 (*C*H_2Ph), 73.6 (*C*H_2Ph), 73.2 (C-5), 73.1 (C-3), 69.3 (C-6), 68.9 (C-4), 56.8 (*C*H_3O).

Methyl 2,6-di-*O*-benzoyl-α-D-galactopyranoside (2α)

Compound **9α** (0.2 g), obtained as described above for the synthesis of **1α**, was dissolved in CH_2Cl_2 (6.6 mL) and cooled to 0 ºC. BzCl (1.5 mL) and pyridine (1.5 mL) were added and the mixture was stirred at rt during 12 h. The excess of BzCl was quenched with water (0.5 mL), and after 0.5 h of stirring, the solution was coevaporated with toluene. Then, the residue was dissolved in 80% HAcO (8.0 mL) and stirred at 65 ºC for 6 h. After concentration under reduced pressure, the residue was diluted with CH_2Cl_2 (50 mL) and washed successively with water, saturated $NaHCO_3$, and water,

dried (Na_2SO_4) and concentrated under reduced pressure. The crude mixture was purified by silica gel column chromatography (3:2, hexane/EtOAc, v/v) to afford **2α** (0.31 g, 77%). R_f 0.37 (1:1 hexane-AcOEt) as a crystalline solid. $[\alpha]_D$ +103 (*c* 1, $CHCl_3$); mp 129-130 ºC; ^{1}H NMR (500 MHz, $CDCl_3$): δ 8.05-7.40 (m, 10H, aromatic), 5.27 (dd, 1H, $J_{1,2}$ = 3.7 Hz, $J_{2,3}$ = 10.1 Hz, H-2), 5.07 (d, 1H, $J_{1,2}$ = 3.7 Hz, H-1), 4.72 (dd, 1H,$J_{5,6a}$ = 6.1 Hz, J_{gem} = 11.4 Hz, H-6a), 4.51 (dd, 1H, $J_{5,6b}$ = 6.8 Hz, J_{gem} = 11.4 Hz, H-6b), 4.25-4.17 (m, 2H, H-3, H-5), 4.11 (m, 1H, H-4), 3.39 (s, 3H, CH_3O), 2.98 (m, 1H, OH-4), 2.88 (m, 1H, OH-3) ppm; ^{13}C NMR (126 MHz, $CDCl_3$): δ 167.0, 166.6 (2 *C*OPh), 133.4, 133.3, 129.9, 129.7, 129.5, 129.4, 128.48, 128.43 (aromatic), 97.5 (C-1), 72.2 (C-2), 69.3 (C-4), 68.2 (C-3), 67.5 (C-5), 63.3 (C-6), 55.4 (*C*H_3O) ppm. ESIMS: *m*/*z* calcd for $C_{21}H_{22}NaO_8$ $[M+Na]^+$ 425.1207. Found: 425.1207.

Methyl 2,6-di-*O*-benzoyl-β-D-galactopyranoside (2β)

The synthetic method was the same used for **2α**. By column chromatography (3:2, hexane/EtOAc, v/v), compound **2β** was afforded in 65% yield as a white crystalline solid. $[\alpha]_D$ -12 (*c* 1, $CHCl_3$); mp 158-159 ºC. ^{1}H NMR (500 MHz, $CDCl_3$): δ 8.08-8.02 (m, 4H, aromatic), 7.60-7.54 (m, 2H, aromatic), 7.48-7.40 (m, 4H, aromatic), 5.24 (dd, 1H,$J_{1,2}$ = 7.9 Hz,$J_{2,3}$ = 9.7 Hz,H-2), 4.70 (dd, 1H,$J_{5,6a}$ = 6.4 Hz, J_{gem} = 11.4 Hz, H-6a), 4.59 (dd, 1H, $J_{5,6b}$ = 6.7 Hz, J_{gem} = 11.4 Hz, H-6b), 4.51 (d, 1H, $J_{1,2}$ = 7.9 Hz, H-1), 4.05 (dd, 1H,$J_{4,5}$ = 0.9 Hz, $J_{3,4}$ = 3.5 Hz, H-4), 3.90 (dt, 1H, $J_{4,5}$ = 0.9 Hz, $J_{5,6a}$ = $J_{5,6b}$ = 6.7 Hz, H-5), 3.84 (dd, 1H, $J_{3,4}$ = 3.5 Hz, $J_{2,3}$ = 9.7 Hz, H-3), 3.51 (s, 3H, CH_3O) ppm; ^{13}C NMR (126 MHz, $CDCl_3$): δ 167.2, 166.6 (2 *C*OPh), 133.4, 133.3, 129.9, 129.7, 129.5, 129.4, 128.49, 128.41 (aromatic), 101.8 (C-1), 74.1 (C-2), 72.8 (C-3), 72.1 (C-5), 68.6 (C-4), 62.7 (C-6), 56.8 (*C*H_3O) ppm. ESIMS: *m*/*z* calcd for $C_{21}H_{22}NaO_8$ $[M+Na]^+$ 425.1207. Found: 425.1205.

General procedure for glycosylations using trichloroacetimidates 3 or 4 as glycosyl donors

A suspension of trichloroacetimidate **3** [3] or **4** [4] (74 mg, 0.10 mmol), glycosyl acceptor **1α/β** or **2 α/β** (0.14 mmol, 1.4 equiv) and 4 Å powdered molecular sieves (0.1 g) in dry CH_2Cl_2 (10 mL) was stirred for 30 min under Ar. Then, the mixture was cooled to −30 °C and TMSOTf (8 μL, 0.04 mmol) was added. When TLC analysis showed optimal conversion (normally after 2 h of stirring at −30 °C), the reaction mixture was quenched with triethylamine, filtered, diluted with CH_2Cl_2 (20 mL), and successively washed with $NaHCO_3$ and water (30 mL). After drying with Na_2SO_4, the solvent was evaporated under reduced pressure and the residue was purified by column chromatography, as indicated in each case.

Methyl 2,3,4,6-tetra-*O*-benzoyl-β-D-galactopyranosyl-(1→3)-2,6-di-*O*-benzyl-α-D-galactopyranoside (10α)

Obtained according to the general procedure by condensation of **3** with **1α**. TLC of the crude product showed a major component of R_f 0.34 and the excess of **1α** (R_f 0.05; 85:15, toluene/EtOAc, v/v). Signals corresponding to OCH_3 groups of two disaccharides were observed in the ^{1}H NMR spectrum of the crude mixture at 3.29 and 3.26 ppm in a 10.3:1 ratio, and assigned to **10α** and **12α**, respectively. In the ^{13}C NMR spectrum was observed that in both products the newly formed interglycosidic linkages were β.

After purification by column chromatography (9:1, toluene/EtOAc, v/v) fractions of R_f 0.34 (85:15, toluene/EtOAc, v/v) afforded a syrupy compound **10α** (0.07 g, 74% based on the donor). $[\alpha]_D$ +54 (*c* 1, $CHCl_3$). ^{1}H NMR (500 MHz, $CDCl_3$): δ 8.12−7.06 (m, 30H, aromatic), 6.00 (d, 1H, $J_{3',4'}$ = 3.5 Hz, H-4'), 5.89 (dd, 1H, $J_{1',2'}$ = 8.0 Hz, $J_{2',3'}$ = 10.4 Hz, H-2'), 5.65 (dd, 1H, $J_{3,4'}$ = 3.5 Hz, $J_{2'-3'}$= 10.4

Hz, H-3'), 5.22 (d, 1H, $J_{1',2'}$ = 8.0 Hz, H-1'), 4.64 (dd, 1H, $J_{5'-6'a}$ = 7.0 Hz, J_{gem} = 11.4 Hz, H-6'a), 4.54, 4.47 (2d, 2H, J_{gem} = 12.0 Hz, C*H2*Ph), 4.45-4.37 (m, 3H, H-1, H-6'b and C*H2*Ph), 4.37 (apparent t, 1H, $J_{5',6'b}$ = 6.1 Hz, $J_{5',6'a}$ = 7.0 Hz, H-5'), 4.18-4.14 (m, 2H, H-4 and C*H2*Ph), 4.12 (dd, 1H, $J_{3,4}$ = 3.1 Hz, $J_{2,3}$ = 9.7 Hz, H-3), 3.85 (apparent t, 1H, $J_{5,6b}$ = 4.6 Hz, $J_{5,6a}$ = 6.9 Hz, H-5), 3.72 (dd, 1H, J_{1-2} = 3.6 Hz, $J_{2,3}$ = 9.7 Hz, H-2), 3.63 (dd, 1H, $J_{5,6a}$ = 6.9 Hz, J_{gem} = 10.2 Hz, H-6a), 3.57 (dd, 1H, $J_{5,6b}$ = 4.6 Hz, J_{gem} = 10.2 Hz, H-6b), 3.29 (s, 3H, CH_3O) ppm. ^{13}C NMR (126 MHz, $CDCl_3$): δ 165.8, 165.5, 165.49, 165.45 (*C*OPh), 138.2, 138.1, 133.6, 133.34, 133.30, 130.0, 129.74, 129.72, 129.3, 129.1, 129.0, 128.94, 128.65, 128.45, 128.41, 128.3, 128.2, 128.1, 127.76, 127.72, 127.57, 127.51, 125.2 (aromatic), 102.1 (C-1'), 98.4 (C-1), 78.5 (C-3), 75.3 (C-2), 73.6 (*C*H_2Ph), 73.4 (*C*H_2Ph), 71.5 (C-5'), 71.4 (C-3'), 69,9 (C-4), 69.77 (C-2'), 69.74 (C-6), 68.3 (C-5), 68.1 (C-4'), 61.9 (C-6'), 55.1 (*C*H_3O) ppm. ESIMS: *m*/*z* calcd for $C_{55}H_{52}NaO_{15}$ $[M+Na]^+$ 975.3198. Found: 975.3176.

No other compound was isolated from the column chromatography.

Methyl 2,3,4,6-tetra-*O*-benzoyl-β-D-galactopyranosyl-(1→3)-2,6-di-*O*-benzyl-β-D-galactopyranoside (10β) and methyl 2,3,4,6-tetra-*O*-benzoyl-β-D-galactopyranosyl-(1→4)-2,6-di-*O*-benzyl-β-D-galactopyranoside (12β)

Obtained according to the general procedure by condensation of **3** with **1β**. The crude mixture showed by TLC, among the excess of **1β** (R_f 0.05; 85:15, toluene/EtOAc, v/v), the presence of a major product of R_f 0.36 and a minor product of R_f 0.19. According to the integration of the ^{1}H NMR signals corresponding to H-4 of both disaccharides (4.11 and 4.18 ppm), they were in a 7.0:1 ratio. After purification by column chromatography (9:1, toluene/EtOAc, v/v) fractions of R_f 0.36 afforded a syrupy compound **10β** (0.07 g, 74%). $[\alpha]_D$ +80 (*c* 1, $CHCl_3$). ^{1}H NMR (500 MHz, $CDCl_3$): δ 8.13–7.13 (m, 30H, aromatic), 5.99 (d, 1H, $J_{3',4'}$ = 3.5 Hz, H-4'), 5.86 (dd, 1H, $J_{1',2'}$ = 8.0 Hz, $J_{2',3'}$ = 10.5 Hz, H-2'), 5.61 (dd, 1H, $J_{3,4'}$ = 3.5 Hz, $J_{2',3'}$= 10.5 Hz, H-3'), 5.25 (d, 1H, $J_{1',2'}$ = 8.0 Hz, H-1'), 4.66 (d, 1H, J_{gem} = 11.0 Hz, CH_2Ph), 4.64 (dd, 1H, $J_{5',6'a}$ = 7.0; J_{gem} = 11.4 Hz, H-6'a), 4.58, 4.54 (2d, 2H, J_{gem} = 12.0 Hz, CH_2Ph), 4.44 (dd, 1H, $J_{5',6'b}$ = 6.1 Hz, J_{gem} = 11.4 Hz, H-6'b), 4.34 (d, 1H, J_{gem} = 11.0 Hz, CH_2Ph), 4.27 (t aparente, 1H, $J_{5',6'b}$ = 6.1 ; $J_{5',6'a}$ = 7.0 Hz, H-5'), 4.23 (d, 1H, $J_{1,2}$ = 7.7 Hz, H-1), 4.00 (d, 1H, $J_{3,4}$ = 3.3 Hz, H-4), 3.82 (dd, 1H, $J_{3,4}$ = 3.3 Hz, $J_{2,3}$ = 9.4 Hz, H-3), 3.75 (dd, 1H, $J_{5,6a}$ = 6.6 Hz, J_{gem} = 10.2 Hz, H-6a), 3.71 (dd, 1H, $J_{5,6b}$ = 5.0 Hz, J_{gem} = 10.2 Hz, H-6b), 3.59 (m, 1H, H-5), 3.59 (dd, 1H, J_{1-2} = 7.7 Hz, $J_{2,3}$ = 9.4 Hz, H-2), 3.51 (s, 3H, CH_3O) ppm. ^{13}C NMR (126 MHz, $CDCl_3$): δ 165.9, 165.5, 165.47, 165.46 (*C*OPh), 138.3, 138.0, 133.6, 133.35, 133.30, 133.2, 130.0, 129.74, 129.70, 129.6, 129.0, 128.8, 128.66, 128.61, 128.4, 128.39, 128.36, 128.26, 128.24, 127.68, 127.67, 127.4 (aromatic), 104.4 (C-1), 101.6 (C-1'), 80.7 (C-3), 78.7 (C-2), 74.7 (*C*H_2Ph), 73.6 (*C*H_2Ph), 73.1 (C-5), 71.5 (C-5'), 71.3 (C-3'), 69.8 (C-2'),

69.4 (C-6), 68.8 (C-4), 68.0 (C-4'), 61.9 (C-6'), 56.8 (CH_3O) ppm. ESIMS: *m/z* calcd for $C_{55}H_{52}NaO_{15}$ $[M+Na]^+$ 975.3198. Found: 975.3211.

Fractions of R_f 0.19 afforded syrupy compound **12β** (3.7 mg, 4%). $[\alpha]_D$ +36 (*c* 0.3, $CHCl_3$). ^{1}H NMR(500 MHz, $CDCl_3$): δ 7.83–7.00 (m, 30H, aromatic), 6.00 (dd, 1H, $J_{3',4'}$ = 0.9 Hz, $J_{3',4'}$ = 3.4 Hz, H-4'), 5.88 (dd, 1H, $J_{1',2'}$ = 8.0 Hz, $J_{2',3'}$ = 10.4 Hz, H-2'), 5.63 (dd, 1H, $J_{3',4'}$ = 3.4 Hz, $J_{2',3'}$ = 10.4 Hz, H-3'), 5.25 (d, 1H, $J_{1',2'}$ = 8.0 Hz, H-1'), 4.63 – 4.52 (m, 4H, 2xCH_2Ph and H-6'a), 4.40 (dd, 1H, $J_{5',6'b}$ = 6.7 Hz, J_{gem} = 11.3 Hz, H-6'b), 4.30 (apparent t, 1H, $J_{5',6'a}$ = 6.2 Hz, $J_{5',6'b}$ = 6.7 Hz, H-5'), 4.21 (d, 1H, $J_{1,2}$ = 7.7 Hz, H-1), 4.18 (d, 1H, $J_{3,4}$ = 3.0 Hz, H-4), 3.90 (dd, 1H, $J_{5,6a}$ = 5.0 ; J_{gem} = 10.4 Hz, H-6a), 3.84 (dd, 1H, $J_{5,6b}$ = 6.4; J_{gem} = 10.4 Hz, H-6b), 3.67 (m, 1H, H-5), 3.63 (dd, 1H, $J_{3,4}$ = 3.0 ; $J_{2,3}$ = 9.7 Hz, H-3), 3.54 (s, 3H, CH_3O), 3.53 (d, 1H, J_{gem} = 11.0 Hz, CH_2Ph), 3.19 (dd, 1H, $J_{1,2}$ = 7.7; $J_{2,3}$ = 9.7 Hz, H-2) ppm. ^{13}C NMR (126 MHz, $CDCl_3$): δ 166.3, 165.9, 165.7, 165.5 (*C*OPh), 138.2, 138.1, 133.5, 133.2, 129.9, 129.88, 129.83, 129.7, 129.1, 128.6, 128.47, 128.40, 128.27, 128.22, 127.7, 127.5, 127.4, 126.3 (aromatic), 104.4 (C-1), 102.2 (C-1'), 79.8 (C-2), 76.2 (C-4), 74.4 (CH_2Ph), 73.69 (C-5), 73.66 (CH_2Ph), 73.5 (C-3), 71.8 (C-3'), 71.0 (C-5'), 70.0 (C-2'), 69.9 (C-6), 68.2 (C-4'), 62.0 (C-6'), 56.8 (CH_3O) ppm. ESIMS: *m/z* calcd for $C_{55}H_{52}NaO_{15}$ $[M+Na]^+$ 975.3198. Found: 975.3180.

Methyl 2,3,4,6-tetra-*O*-benzoyl-β-D-galactopyranosyl-(1→3)-2,6-di-*O*-benzoyl-α-D-galactopyranoside (11α) and methyl 2,3,4,6-tetra-*O*-benzoyl-β-D-galactopyranosyl-(1→4)-2,6-di-*O*-benzoyl-α-D-galactopyranoside (13α)

Obtained according to the general procedure by condensation of **3** with **2α**. The crude mixture showed by TLC, among the excess of **2α** (R_f 0.05; 85:15, toluene/EtOAc, v/v), a major product

of R_f 0.37 and a minor product of R_f 0.34 in 10.8:1 ratio, according to the integration of the ^{1}H NMR signals corresponding to H-2 of both disaccharides (5.38 and 4.90 ppm). After purification by column chromatography (9:1, toluene/EtOAc, v/v), fractions of R_f 0.37 afforded a syrupy compound **11α** (0.07 g, 71%). $[\alpha]_D$ +77 (*c* 1, $CHCl_3$). ^{1}H NMR(500 MHz, $CDCl_3$): δ 8.12-7.00 (m, 30H, aromatic), 5.97 (dd, 1H, $J_{4',5'}$ = 0.9 Hz, $J_{3',4'}$ = 3.5 Hz, H-4'), 5.77 (dd, 1H, $J_{1',2'}$ = 8.0 Hz, $J_{2',3'}$ = 10.4 Hz, H-2'), 5.53 (dd, 1H, $J_{3',4'}$ = 3.5 Hz, $J_{2',3'}$ = 10.4 Hz, H-3'), 5.38 (dd, 1H, $J_{1,2}$ = 3.7 Hz, $J_{2,3}$ = 9.7 Hz, H-2), 5.12 (d, 1H, $J_{1',2'}$ = 8.0 Hz, H-1'), 5.02 (d, 1H, $J_{1,2}$ = 3.7 Hz, H-1), 4.68 (dd, 1H, $J_{5',6'a}$ = 7.3 Hz, J_{gem} = 11.4 Hz, H-6'a), 4.55 (dd, 1H, $J_{5,6a}$ = 7.9 Hz, J_{gem} = 11.7 Hz, H-6a), 4.48 (dd, 1H, $J_{5'-6'b}$ = 5.6 Hz, J_{gem} = 11.4 Hz, H-6'b), 4.43 (dd, 1H, $J_{5,6b}$ = 4.8 Hz, J_{gem} = 11.7 Hz, H-6b), 4.40 (ddd, 1H, $J_{4',5'}$ = 0.9 Hz, $J_{5'-6'b}$ = 5.6 Hz, $J_{5',6'a}$ = 7.3 Hz, H-5'), 4.35-4.30 (m, 2H, H-3 and H-4), 4.06 (dd, 1H, $J_{5,6b}$ = 4.8 Hz, $J_{5,6a}$ = 7.9 Hz, H-5), 3.30 (s, 3H, CH_3O) ppm. ^{13}C NMR (126 MHz, $CDCl_3$): δ 166.2, 166.0, 165.6, 165.4, 165.3, 165.0 (*C*OPh), 133.7, 133.4, 133.3, 133.09, 133.05, 132.9, 130.0, 129.7, 129.6, 129.58, 129.55, 129.3, 128.70, 128.58, 128.55, 128.47, 128.42, 128.3, 128.2, 128.0 (aromatic), 101.9 (C-1'), 97.0 (C-1), 76.7 (C-3), 71.7 (C-5'), 71.4 (C-3'), 70.1 (C-2), 69.5 (C-4), 69.4 (C-2'), 67.9 (C-4'), 67.5 (C-5), 64.2 (C-6), 62.0 (C-6'), 55.1 (*C*H_3O) ppm. ESIMS: *m*/*z* calcd for $C_{55}H_{48}NaO_{17}$ $[M+Na]^+$ 1003.2784. Found: 1003.2778.

Further elution from the column afforded compound **13α** (4,1 mg, 4%), impurified with **11α**. ^{1}H NMR (500 MHz, $CDCl_3$) δ 8.13-7.14 (m, 30H, aromatic), 5.98 (d, 1H, $J_{3',4'}$ = 3.4 Hz, H-4'), 5.90 (dd, 1H, $J_{1',2'}$ = 8.0 Hz, $J_{2',3'}$ = 10.4 Hz, H-2'), 5.60 (dd, 1H, $J_{2',3'}$ = 10.4 Hz, $J_{3',4'}$ = 3.4 Hz, H-3'), 5.24 (d, 1H, $J_{1',2'}$ = 8.0 Hz, H-1'), 5.03 (d, 1H, $J_{1,2}$ = 3.6 Hz, H-1), 4.90 (dd, 1H, $J_{1,2}$ = 3.6 Hz, $J_{2,3}$ = 10.4 Hz, H-2), 4.78 (dd, 1H, $J_{5,6a}$ = 4.5 Hz, J_{gem} = 10.8 Hz, H-6a), 4.62 (dd, 1H, $J_{5,6b}$ = 7.6 Hz, J_{gem} = 10.8 Hz, H-6b), 4.57 (dd, 1H, $J_{5',6'a}$ = 6.3 Hz, J_{gem} = 11.3 Hz, H-6'a), 4.40 (dd, 1H, $J_{5',6'b}$ =

6.7 Hz, J_{gem} = 11.3 Hz, H-6’b), 4.36-4.30 (m, 2H, H-4 and H-5'), 4.26-4.20 (m, 2H, H-3 and H-5), 3.32 (s, 3H, C*H*$_3$O) ppm. ^{13}C NMR (126 MHz, $CDCl_3$): δ 166.1, 165.96, 165.92, 165.58, 165.57, 165.1 (*C*OPh), 135.7, 133.5, 133.24, 133.20, 133.1, 133.0, 132.9, 130.0, 129.8, 129.79, 129.76, 129.56, 128.9, 128.7, 128.6, 128.45, 128.42, 128.27, 128.2 (aromatic), 102.4 (C-1'), 97.2 (C-1), 77.8 (C-4), 72.3 (C-2), 71.8 (C-3'), 71.3 (C-5'), 70.1 (C-2'), 68.8 (C-5), 68.0 (C-4'), 67.7 (C-3), 64.3 (C-6), 61.8 (C-6'), 55.3 (*C*H$_3$O) ppm.

Methyl 2,3,4,6-tetra-*O*-benzoyl-β-D-galactopyranosyl-(1→3)-2,6-di-*O*-benzoyl-β-D-galactopyranoside (11β) and methyl 2,3,4,6-tetra-*O*-benzoyl-β-D-galactopyranosyl-(1→4)-2,6-di-*O*-benzoyl-β-D-galactopyranoside (13β)

Obtained according to the general procedure by condensation of **3** with **2β**. The crude mixture showed by TLC the presence of a major product of R_f 0.34 and a minor product of R_f 0.36 (9:1, toluene/EtOAc, v/v) in a 5.7:1 ratio, according to the integration of the ^{1}H NMR signals corresponding to H-1′ of both disaccharides (5.01 and 5.29 ppm). After purification by column chromatography (91:9 → 87:13, toluene/AcOEt, v/v), fractions of R_f 0.36 afforded a mixture of **13β** and some hydrolyzed donor **3** (0.01 g, 10%).

Selected signals for **13β**: ^{1}H NMR (500 MHz, $CDCl_3$): δ 5.31 (d, 1H, $J_{1',2'}$ = 8.0 Hz, H-1'), 4.48 (d, 1H, $J_{1,2}$ = 7.8 Hz, H-1) ppm; ^{13}C NMR (126 MHz, $CDCl_3$): 102.4 (C-1'), 101.6 (C-1), 76.9 (C-4), 73.9 (C-2), 73.6 (C-3), 72.2 (C-3'), 72.0 (C-5), 71.4 (C-5'), 70.2 (C-2'), 68.1 (C-4'), 64.0 (C-6), 61.9 (C-6'), 56.7 (*C*H$_3$O) ppm.

Fractions of R_f 0.34 afforded syrupy compound **11β** (0.06 g, 62%). $[\alpha]_D$ +90 (*c* 1, $CHCl_3$), in agreement with reported data [5]. ^{1}H NMR(500 MHz, $CDCl_3$): δ 8.13-7.08 (m, 30H, aromatic),

5.94 (dd, 1H, $J_{4',5'}$ = 0.9 Hz, $J_{3',4'}$ = 3.3 Hz, H-4'), 5.76 (dd, 1H, $J_{1',2'}$ = 8.0 Hz, $J_{2',3'}$ = 10.4 Hz, H-2'), 5.50 (dd, 1H, $J_{1,2}$ = 8.0 Hz, $J_{2,3}$ = 9.8 Hz, H-2), 5.48 (dd, 1H, $J_{3',4'}$ = 3.5 Hz, $J_{2',3'}$ = 10.4 Hz, H-3'), 5.01 (d, 1H, $J_{1',2'}$ = 8.0 Hz, H-1'), 4.60 (dd, 1H, $J_{5',6'a}$ = 7.4 Hz, J_{gem} = 11.6 Hz, H-6'a), 4.59 (dd, 1H, $J_{5,6a}$ = 7.5 Hz, J_{gem} = 10.6 Hz, H-6a), 4.49 (dd, 1H, $J_{5',6'b}$ = 5.4 Hz, J_{gem} = 11.6 Hz, H-6'b), 4.46 (dd, 1H, $J_{5,6b}$ = 4.7 Hz, J_{gem} = 10.6 Hz, H-6b), 4.41 (d, 1H, $J_{1,2}$ = 8.0 Hz, H-1), 4.34 (ddd, 1H, $J_{4',5'}$ = 0.9 Hz, $J_{5',6'b}$ = 5.4 Hz, $J_{5',6'a}$ = 7.4 Hz, H-5'), 4.27 (bs, 1H, H-4), 3.99 (dd, 1H, $J_{3,4}$ = 3.3 Hz, $J_{2,3}$ = 9.8 Hz, H-3), 3.83 (m, 1H, H-5), 3.39 (s, 3H, C*H*$_3$O), 3.07 (bs, 1H, OH-4) ppm. ^{13}C NMR (126 MHz, CDCl$_3$): δ 166.2, 166.0, 165.6, 165.4, 164.8, 164.7 (*C*OPh), 133.7, 133.5, 133.3, 133.1, 132.7, 132.6, 130.0, 129.7, 129.66, 129.63, 129.44, 129.40, 128.9, 128.7, 128.69, 128.63, 128.5, 128.4, 128.2, 128.1, 128.0 (aromatic), 101.79 (C-1), 101.74 (C-1'), 81.0 (C-3), 72.0 (C-5), 71.8 (C-5'), 71.3 (C-3'), 70.3 (C-2), 69.3 (C-2'), 68.4 (C-4), 67.8 (C-4'), 63.6 (C-6), 62.1 (C-6'), 56.3 (*C*H$_3$O) ppm. ESIMS: *m*/*z* calcd for $C_{55}H_{48}NaO_{17}$ $[M+Na]^+$ 1003.2784. Found: 1003.2777.

Methyl 2,3,5,6-tetra-*O*-benzoyl-β-D-galactofuranosyl-(1→3)-2,6-di-*O*-benzyl-α-D-galactopyranoside (14α) and methyl 2,3,5,6-tetra-*O*-benzoyl-β-D-galactofuranosyl-(1→4)-2,6-di-*O*-benzyl-α-D-galactopyranoside (16α)

Obtained according to the general procedure by condensation of **4** with **1α**. The crude mixture showed by TLC the presence of a major product of R$_f$ 0.45 and a minor product of *R*$_f$ 0.40 (85:15, toluene/EtOAc, v/v) in 3.0:1 ratio, according to the integration of the ^{1}H NMR signals. After purification by column chromatography (90:10→86:14, toluene/EtOAc, v/v), fractions of *R*$_f$ 0.45 afforded syrupy compound **14α** (0.05 g, 53%). $[\alpha]_D$ +7 (*c* 1, CHCl$_3$). ^{1}H NMR (500 MHz, CDCl$_3$): δ 8.09-7.13 (m, 30H, aromatic), 6.02 (m, 1H, H-5'), 5.75 (s, 1H, H-1'), 5.67 (dd, 1H, $J_{2',3'}$ = 1.3 Hz,

$J_{3',4'}$ = 5.1 Hz, H-3'), 5.65 (d, 1H, $J_{2',3'}$ = 1.3 Hz, H-2'), 4.78 (d, 1H, J_{gem} = 12.4 Hz, C*H*2Ph), 4.77-4.70 (m, 3H, H-4', H-6'a and H-6'b), 4.69 (d, 1H, $J_{1,2}$ = 3.6 Hz, H-1), 4.65 (d, 1H, J_{gem} = 12.4 Hz, C*H*2Ph), 4.56, 4.52 (2d, 2H, J_{gem} = 11.9 Hz, C*H*2Ph), 4.16 (dd, 1H, $J_{3,4}$ = 3.2 Hz, $J_{2,3}$ = 9.9 Hz, H-3), 4.10 (dd, 1H, $J_{4,5}$ = 1.1 Hz, $J_{3,4}$ = 3.0 Hz, H-4), 3.96-3.91 (m, 2H, H-2 and H-5), 3.66 (d, 2H, $J_{5,6}$ = 5.3 Hz, H-6a and H-6b), 3.42 (s, 3H, C*H*3O) ppm. ^{13}C NMR (126 MHz, $CDCl_3$): δ 166.0, 165.6, 165.5, 165.1 (*C*OPh), 138.1, 137.8, 133.5, 133.3, 133.2, 133.0, 129.9, 129.8, 129.7, 129.6, 129.4, 129.3, 128.8, 128.7, 128.5, 128.3, 128.2, 128.0, 127.7, 127.6, 127.5 (aromatic), 107.2 (C-1'), 98.3 (C-1), 81.8 (C-4'), 81.6 (C-2'), 77.6 (C-3'), 76.1 (C-3), 75.3 (C-2), 73.55 (*C*H_2Ph), 73.50 (*C*H_2Ph), 70.5 (C-4), 70.2 (C-5'), 69.8 (C-6), 68.2 (C-5), 63.3 (C-6'), 55.3 (*C*H_3O) ppm. ESIMS: *m*/*z* calcd for $C_{55}H_{52}NaO_{15}$ $[M+Na]^+$ 975.3198. Found: 975.3201.

Fractions of R_f 0.40 afforded syrupy compound **16α** (0.02 g, 21%). $[\alpha]_D$ +10 (*c* 1, $CHCl_3$). ^{1}H NMR (500 MHz, $CDCl_3$): δ 8.10-7.15 (m, 30H, aromatic), 5.96 (m, 1H, $J_{5',6'b}$ = 3.5 Hz, $J_{4',5'}$ = 3.7 Hz, $J_{5',6'a}$ = 7.6 Hz, H-5'), 5.80 (s, 1H, H-1'), 5.65 (dd, 1H, $J_{2',3'}$ = 1.8 Hz, $J_{3',4'}$ = 5.5 Hz, H-3'), 5.56 (d, 1H, $J_{2',3'}$ = 1.8 Hz, H-2'), 4.76 (d, 1H, J_{gem} = 12.0 Hz, C*H*2Ph), 4.71 (d, 1H, $J_{1,2}$ = 3.5 Hz, H-1), 4.69 (d, 1H, J_{gem} = 12.0 Hz, C*H*2Ph), 4.65 (dd, 1H, $J_{5',6'a}$ = 7.6 Hz, J_{gem} = 12.0 Hz, H-6'a), 4.62 (dd, 1H, $J_{4',5'}$ = 3.7 Hz, $J_{3',4'}$ = 5.5 Hz, H-4'), 4.51 (dd, 1H, $J_{5',6'b}$ = 3.5 Hz, J_{gem} = 12.0 Hz, H-6'b), 4.45, 4.38 (2d, 2H, J_{gem} = 11.7 Hz, C*H*2Ph), 4.23 (dd, 1H, $J_{4,5}$ = 0.7 Hz, $J_{3,4}$ = 3.0 Hz, H-4), 4.10 (dd, 1H, $J_{3,4}$ = 3.0 Hz, $J_{2,3}$ = 10.1 Hz, H-3), 4.01 (ddd, 1H, $J_{4,5}$ = 0.7 Hz, $J_{5,6a}$ = 5.5 Hz, $J_{5,6b}$ = 6.8 Hz, H-5), 3.89 (dd, 1H, $J_{1,2}$ = 3.5 Hz, $J_{2,3}$ = 10.1 Hz, H-2), 3.72 (dd, 1H, $J_{5,6a}$ = 5.5 Hz, J_{gem} = 9.9 Hz, H-6a), 3.60 (dd, 1H, $J_{5,6b}$ = 6.8 Hz, J_{gem} = 9.9 Hz, H-6b), 3.35 (s, 3H, C*H*3O) ppm. ^{13}C NMR (126 MHz, $CDCl_3$): δ 166.0, 165.8, 165.6, 165.5 (*C*OPh), 138.0, 137.8, 133.5, 133.4, 133.1, 133.0, 129.99, 129.92, 129.91, 129.7, 129.5, 129.4, 129.0, 128.8, 128.5, 128.39, 128.36, 128.34, 128.1, 127.9, 127.6, 127.5

(aromatic), 106.9 (C-1'), 98.1 (C-1), 82.6 (C-2'), 81.5 (C-4'), 77.4 (C-3'), 77.1 (C-2), 74.2 (C-4), 73.4 (CH_2Ph), 73.2 (CH_2Ph), 70.4 (C-3), 70.3 (C-5'), 69.4 (C-6), 68.7 (C-5), 63.7 (C-6'), 55.3 (CH_3O) ppm. ESIMS: *m*/*z* calcd for $C_{55}H_{52}NaO_{15}$ $[M+Na]^+$ 975.3198. Found: 975.3202.

Methyl 2,3,5,6-tetra-*O*-benzoyl-β-D-galactofuranosyl-(1→3)-2,6-di-*O*-benzyl-β-D-galactopyranoside (14β) and methyl 2,3,5,6-tetra-*O*-benzoyl-β-D-galactofuranosyl-(1→4)-2,6-di-*O*-benzyl-β-D-galactopyranoside (16β)

Obtained according to the general procedure by condensation of **4** with **1β.** The crude mixture showed by TLC only one spot of R_f 0.45 (85:15, toluene/EtOAc, v/v) in a 1.8:1 ratio, according to the integration of the ^{1}H NMR spectrum. After purification by column chromatography (9:1, hexane/EtOAc, v/v), the first fractions afforded a syrupy compound **14β** (7.5 mg, 8%). $[\alpha]_D$ -6 (*c* 0.7, $CHCl_3$). ^{1}H NMR (500 MHz, $CDCl_3$): δ 8.13-7.00 (m, 30H, aromatic), 5.99 (ddd, 1H, $J_{4',5'}$ = 4.4 Hz, $J_{5',6'a}$ = 4.6 Hz, $J_{5',6'b}$ = 6.7 Hz, H-5'), 5.68 (s, 1H, H-1'), 5.64 (dd, 1H, $J_{2',3'}$ = 1.2 Hz, $J_{3',4'}$ = 5.0 Hz, H-3'), 5.59 (d, 1H, $J_{2',3'}$ = 1.2 Hz, H-2'), 4.85 (d, 1H, J_{gem} = 11.0 Hz, CH_2Ph), 4.74 (dd, 1H, $J_{5',6'a}$ = 4.6 Hz, J_{gem} = 11.9 Hz, H-6'a), 4.72 (d, 1H, J_{gem} = 11.0 Hz, CH_2Ph), 4.72-4.71 (m, 3H, H-4'), 4.68 (dd, 1H, $J_{5',6'b}$ = 6.7 Hz, J_{gem} = 11.9 Hz, H-6'b), 4.54 (s, 2H, CH_2Ph), 4.28 (d, 1H, $J_{1,2}$ = 7.7 Hz, H-1), 4.00 (m, 1H, H-4), 3.75 (dd, 1H, $J_{3,4}$ = 3.3 Hz, $J_{2,3}$ = 9.5 Hz, H-3), 3.73-3.66 (m, 3H, H-2, H-6a and H-6b), 3.62-3.56 (m, 4H, H-5 and CH_3O), 2.50 (d, 1H, $J_{4,OH}$ = 2.4 Hz, OH-4) ppm. ^{13}C NMR (126 MHz, $CDCl_3$): δ 166.0, 165.6, 165.5, 165.1 (*C*OPh), 138.3, 137.9, 133.5, 133.3, 133.2, 133.1, 129.9, 129.8, 129.7, 129.4, 129.3, 128.87, 128.81, 128.5, 128.4, 128.3, 128.2, 128.1, 127.7, 127.5, (aromatic), 107.1 (C-1'), 104.7 (C-1), 81.9 (C-4'), 81.6 (C-2'), 78.9 (C-3), 78.3 (C-2),

77.5 (C-3'), 74.9 (CH_2Ph), 73.6 (CH_2Ph), 73.1 (C-5), 70.2 (C-5'), 69.6 (C-4), 69.4 (C-6), 63.2 (C-6'), 56.9 (CH_3O) ppm. ESIMS: *m*/*z* calcd for $C_{55}H_{52}NaO_{15}$ $[M+Na]^+$ 975.3198. Found: 975.3188. Further elution of the column afforded 0.06 g (64%) of a mixture of **14β** and **16β**. ^{1}H NMR (500 MHz, $CDCl_3$) anomeric signals: δ 5.68 (s, 1H, H-1'), 4.23 (d, 1H, $J_{1,2}$ = 7.5 Hz, H-1) ppm. ^{13}C NMR (126 MHz, $CDCl_3$) anomeric signals: δ 106.9 (C-1'), 104.8 (C-1) ppm.

Methyl 2,3,5,6-tetra-*O*-benzoyl-β-D-galactofuranosyl-(1→3)-2,6-di-*O*-benzoyl-α-D-galactopyranoside (15α) and methyl 2,3,5,6-tetra-*O*-benzoyl-β-D-galactofuranosyl-(1→4)-2,6-di-*O*-benzoyl-α-D-galactopyranoside (17α)

Obtained according to the general procedure by condensation of **4** with **2α**. After 2 h of reaction, TLC analysis showed total consumption of **4** and two products of R_f 0.32 and 0.25 (85:15, toluene/EtOAc, v/v) in a 1:7.3 ratio, according to the integration of signals corresponding to H-1 of both products. Purification by column chromatography (toluene/EtOAc, 87:13, v/v) afforded a fraction containing **17α** impurified with **15α** (0.02 g, 21%). From the mixture, signals corresponding to **17α**: ^{1}H NMR (500 MHz, $CDCl_3$) δ 8.15-7.24 (m, 30H, aromatic), 5.99 (ddd, 1H, $J_{4',5'}$ = 4.3 Hz, $J_{5',6'a}$ = 4.3 Hz, $J_{5',6'b}$ = 6.4 Hz, H-5'), 5.84 (dd, 1H, $J_{2',3'}$ = 2.4 Hz, $J_{3',4'}$ = 6.2 Hz, H-3'), 5.66 (s, 1H, H-1'), 5.54 (dd, 1H, $J_{1',2'}$ = 0.6 Hz, $J_{2',3'}$ = 2.4 Hz, H-2'), 5.41 (dd, 1H, $J_{1,2}$ = 3.6 Hz, $J_{2,3}$ = 10.3 Hz, H-2), 5.13 (d, 1H, $J_{1,2}$ = 3.6 Hz, H-1), 4.95 (dd, 1H, $J_{4',5'}$ = 4.3 Hz, $J_{3',4'}$ = 6.2 Hz, H-4'), 4.74 (dd, 1H, $J_{5',6'a}$ = 4.3 Hz, J_{gem} = 12.0 Hz, H-6'a), 4.69 (dd, 1H, $J_{5',6'b}$ = 6.4 Hz, J_{gem} = 12.0 Hz, H-6'b), 4.63 (dd, 1H, $J_{5,6a}$ = 8.0 Hz, J_{gem} = 11.4 Hz, H-6a), 4.56 (dd, 1H, $J_{5,6b}$ = 4.4 Hz, J_{gem} = 11.4 Hz, H-6b), 4.33-4.26 (m, 3H, H-3, H-4 and H-5), 3.39 (s, 3H, CH_3O), 3.28 (d, 1H, $J_{3,OH}$ = 10.4 Hz, OH-3) ppm. ^{13}C NMR (126 MHz, $CDCl_3$): δ 166.77, 166.73, 166.0, 165.9,

165.6, 165.5 (COPh), 133.6, 133.5, 133.4, 133.23, 133.20, 133.1, 132.9, 130.09, 130.06, 130.01, 129.9, 129.8, 129.74, 129.70, 129.59, 129.50, 129.3, 128.9, 128.6, 128.5, 128.4, 128.39, 128.35, 128.31 (aromatic), 108.4 (C-1'), 97.5 (C-1), 84.2 (C-2'), 81.1 (C-4'), 77.5 (C-4), 76.7 (C-3'), 72.2 (C-2), 70.4 (C-5'), 68.8 (C-5), 67.6 (C-3), 64.1 (C-6), 63.2 (C-6'), 55.3 (CH_3O) ppm.

Further elution of the column afforded pure **15α** (0.06 g, 62%). $[\alpha]_D$ +33 (c 1, $CHCl_3$). ^{1}H NMR (500 MHz, $CDCl_3$): δ 8.08-7.21 (m, 30H, aromatic), 5.98 (ddd, 1H, $J_{4',5'}$ = 4.4 Hz, $J_{5',6'a}$ = 4.7 Hz, $J_{5',6'b}$ = 6.4 Hz, H-5'), 5.64 (dd, 1H, $J_{2',3'}$ = 1.6 Hz, $J_{3',4'}$ = 5.2 Hz, H-3'), 5.63 (s, 1H, H-1'), 5.47 (dd, 1H, $J_{1',2'}$ = 0.7 Hz, $J_{2',3'}$ = 1.6 Hz, H-2'), 5.46 (dd, 1H, $J_{1,2}$ = 3.7 Hz, $J_{2,3}$ = 10.2 Hz, H-2), 5.15 (d, 1H, $J_{1,2}$ = 3.7 Hz, H-1), 4.79 (dd, 1H, $J_{5',6'a}$ = 4.7 Hz, J_{gem} = 11.9 Hz, H-6'a), 4.77 (apparent t, 1H, $J_{4',5'}$ = 4.4 Hz, $J_{3',4'}$ = 5.2 Hz, H-4'), 4.71 (dd, 1H, $J_{5',6'b}$ = 6.4 Hz, J_{gem} = 11.9 Hz, H-6'b), 4.55 (dd, 1H, $J_{5,6a}$ = 7.6 Hz, J_{gem} = 11.6 Hz, H-6a), 4.49 (dd, 1H, $J_{5,6b}$ = 4.5 Hz, J_{gem} = 11.6 Hz, H-6b), 4.40 (dd, 1H, $J_{3,4}$ = 3.3 Hz, $J_{2,3}$ = 10.2 Hz, H-3), 4.24 (dd, 1H, $J_{4,5}$ = 1.0 Hz, $J_{3,4}$ = 3.3 Hz, H-4), 4.18 (ddd, 1H, $J_{4,5}$ = 1.0 Hz, $J_{5,6b}$ = 4.5 Hz, $J_{5,6a}$ = 7.6 Hz, H-5), 3.37 (s, 3H, CH_3O) ppm. ^{13}C NMR (126 MHz, $CDCl_3$): δ 166.2, 166.1, 166.0, 165.6, 165.5, 164.9 (COPh), 133.6, 133.3, 133.29, 133.25, 133.09, 133.0, 129.9, 129.83, 129.82, 129.72, 129.70, 129.5, 129.37, 129.31, 128.6, 128.5, 128.43, 128.41, 128.3, 128.2 (aromatic), 107.3 (C-1'), 97.2 (C-1), 82.1 (C-4'), 81.9 (C-2'), 77.08 (C-3'), 74.9 (C-3), 70.4 (C-2), 70.3 (C-5'), 69.7 (C-4), 67.5 (C-5), 64.0 (C-6), 63.0 (C-6'), 55.2 (CH_3O) ppm. ESIMS: m/z calcd for $C_{55}H_{48}NaO_{17}$ $[M+Na]^+$ 1003.2784. Found: 1003.2777.

Methyl 2,3,5,6-tetra-*O*-benzoyl-β-D-galactofuranosyl-(1→3)-2,6-di-*O*-benzoyl-β-D-galactopyranoside (15β)

Obtained according to the general procedure by condensation of **4** with **2β.** After 2 h of reaction, TLC analysis showed total consumption of **4** and the formation of a single product (R_f 0.32; 85:15, toluene/EtOAc, v/v). After purification by column chromatography (9:1, toluene/EtOAc, v/v), compound **15β** was obtained (0.08 g, 83%). $[\alpha]_D$ +14 (*c* 1, $CHCl_3$). ^{1}H NMR (500 MHz, $CDCl_3$): δ 8.07-7.17 (m, 30H, aromatic), 5.94 (ddd, 1H, $J_{4',5'}$ = 4.3 Hz, $J_{5',6'b}$ = 4.7 Hz, $J_{5',6'a}$ = 6.3 Hz, H-5'), 5.61 (dd, 1H, $J_{2',3'}$ = 1.7 Hz, $J_{3',4'}$ = 5.1 Hz, H-3'), 5.58 (dd, 1H, $J_{1,2}$ = 8.0 Hz, $J_{2,3}$ = 9.9 Hz, H-2), 5.46 (s, 1H, H-1'), 5.38 (d, 1H, $J_{2',3'}$ = 1.7 Hz, H-2'), 4.77 (dd, 1H, $J_{4',5'}$ = 4.3 Hz, $J_{3',4'}$ = 5.1 Hz, H-4'), 4.75 (dd, 1H, $J_{5',6'a}$ = 4.7 Hz, J_{gem} = 11.8 Hz, H-6’a), 4.68 (dd, 1H, $J_{5',6'b}$ = 6.3 Hz, J_{gem} = 11.8 Hz, H-6’b), 4.60 (dd, 1H, $J_{5,6a}$ = 7.2 Hz, J_{gem} = 11.6 Hz, H-6a), 4.53 (dd, 1H, $J_{5,6b}$ = 5.3 Hz, J_{gem} = 11.6 Hz, H-6b), 4.48 (d, 1H, $J_{1,2}$ = 8.0 Hz, H-1), 4.19 (dd, 1H, $J_{4,OH}$ = 2.9 Hz, $J_{3,4}$ = 3.4 Hz, H-4), 4.02 (dd, 1H, $J_{3,4}$ = 3.4 Hz, $J_{2,3}$ = 9.9 Hz, H-3), 6.05 (ta, 1H, H-5), 3.42 (s, 3H, CH_3O), 2.70 (d, 1H, $J_{4,OH}$ = 2.9 Hz, OH-4) ppm. ^{13}C NMR (126 MHz, $CDCl_3$): δ 166.3, 166.0, 165.6, 165.5, 165.2, 164.9 (*C*OPh), 133.6, 133.3, 133.24, 133.23, 133.1, 132.8, 129.9, 129.87, 129.83, 129.79, 129.75, 129.71, 129.67, 129.64, 129.3, 129.2, 128.9, 128.59, 128.57, 128.55, 128.53, 128.50, 128.4, 128.3, 128.2 (aromatic), 107.1 (C-1'), 102.0 (C-1), 82.2 (C-4'), 82.1 (C-2'), 79.0 (C-3), 76.8 (C-3'), 72.2 (C-5), 70.7 (C-2), 70.3 (C-5'), 68.7 (C-4), 63.3 (C-6), 62.9 (C-6'), 56.5 (*C*H_3O) ppm. ESIMS: *m*/*z* calcd for $C_{55}H_{48}NaO_{17}$ $[M+Na]^+$ 1003.2784. Found: 1003.2776.

General procedure for the glycosylations using 5 as glycosyl donor

A solution of 1,2,3,5,6-penta-*O*-tert-butyldimethylsilyl-β-D-galactofuranose (75 mg, 0.1 mmol) in anhydrous CH_2Cl_2 (7.5 mL) containing dry 4 Å powdered molecular sieves (0.1 g) was cooled to 0 °C and stirred for 10 min under Ar. Then, iodotrimethylsilane (0.014 mL, 0.12 mmol, 1.2 equiv) was added, and the solution was stirred at 0 °C until TLC monitoring showed complete transformation of the starting compound to two products with R_f 0.70 and 0.54 (10:1, hexane/EtOAc, v/v), respectively. Then, EtN(*i*-Pr)$_2$ (0.021 mL, 0.12 mmol) and a solution of the acceptor (1.4 equiv, 0.14 mmol) in CH_2Cl_2 (2.5 mL) were added by syringe, and the stirring was continued until consumption of the components with R_f 0.70 and 0.54. The solution was diluted with CH_2Cl_2, washed with $NaHCO_3$ and water, dried (Na_2SO_4), and concentrated. The syrup obtained was purified by column chromatography, as indicated in each individual case.

Methyl 2,3,5,6-tetra-*O*-*tert*-butyldimethylsilyl-β-D-galactofuranosyl-(1→3)-2,6-di-*O*-benzyl-α-D-galactopyranoside (18α) and methyl 2,3,5,6-tetra-*O*-*tert*-butyldimethylsilyl-β-D-galactofuranosyl-(1→4)-2,6-di-*O*-benzyl-α-D-galactopyranoside (19α)

Obtained according to the general procedure by condensation of **5**, with **1α**.^{1}H NMR analysis of the crude product showing the formation of three products in a 6.5:2.7:1 ratio, assigned as **18α**, **19α**, and **20α** (the analog of **18α** with the interglycosidic linkage in the α-configuration). TLC analysis of the crude mixture showed components of R_f 0.27 and 0.11 (hexane/EtOAc, 10:1, v/v). The mixture was purified by column chromatography (94:6 hexane-EtOAc). Fractions of *Rf* 0.27 afforded compound **18α** (0.02 g, 20%), $[\alpha]_D$ +4 (*c* 1, $CHCl_3$). ^{1}H NMR (500 MHz, $CDCl_3$): 1H NMR (500 MHz, CDCl3): δ 7.37-7.24 (m, 10H, aromatic), 5.17 (s,

1H, H-1'), 4.86 (d, 1H, J_{gem} = 11.2 Hz, CH_2Ph), 4.64-4.62 (m, 1H, H-1), 4.63, 4.59 (2d, 2H, J_{gem} = 12.0 Hz, CH_2Ph), 4.53 (d, 1H, J_{gem} = 11.2 Hz, CH_2Ph), 4.12-4.09 (m, 3H, H-2', H-3' and H-4), 3.99-3.94 (m, 3H, H-3, H-4' and H-5), 3.77-3.64 (m, 5H, H-2, H-5', H-6a, H-6b and H-6'a), 3.57 (dd, 1H, $J_{5',6'b}$ = 5.3, J_{gem} = 10.5 Hz, H-6'b), 3.40 (s, 3H, CH_3O), 0.90, 0.896, 0.890, 0.88 (4s, 36H, Si(CH$_3$)$_2$C(CH_3)$_3$), 0.13, 0.11, 0.10, 0.09, 0.06 (5s, 15H, Si(CH_3)$_2$C(CH$_3$)$_3$), 0.06-0.05 (m, 9H, Si(CH_3)$_2$C(CH$_3$)$_3$) ppm. ^{13}C NMR (126 MHz, CDCl$_3$): δ 138.49, 138.43, 128.3, 128.2, 127.9, 127.7, 127.48, 127.42 (aromatic), 109.0 (C-1'), 98.5 (C-1), 89.3 (C-4'), 83.0 (C-2'), 78.8 (C-3'), 77.1 (C-3), 74.5 (C-2), 74.4 (C-5'), 73.4 (*C*H$_2$Ph), 73.3 (*C*H$_2$Ph), 70.0 (C-6), 69.8 (C-4), 68.4 (C-5), 65.6 (C-6'), 55.2 (*C*H$_3$O), 26.0, 25.9, 25.74, 25.70 (Si(CH$_3$)$_2$C(*C*H$_3$)$_3$), 18.4, 18.3, 17.88, 17.87 (Si(CH$_3$)$_2$*C*(CH$_3$)$_3$), -4.31, -4.38, -4.4, -4.5, -4.6, -4.9, -5.1, -5.3 (Si(*C*H$_3$)$_2$C(CH$_3$)$_3$) ppm. ESIMS: *m*/*z* calcd for $C_{51}H_{93}O_{11}Si_4$ $[M+H]^+$ 993.5789. Found: 993.5781.

Fractions of R_f 0.11 afforded a mixture of **19α** and **20α** (0.05 g, 50%). ^{1}H NMR (500 MHz, CDCl$_3$) anomeric region: δ 5.25 (s, 1H, H-1', **19α**), 4.89 (d, 0.3H, $J_{1',2'}$ = 4.5 Hz, H-1', **20α**), 4.68 (d, 1H, $J_{1,2}$ = 3.4 Hz, H-1, **19α**), 4.58 (d, 0.3H, $J_{1,2}$ = 3.5 Hz, H-1, **20α**) ppm. ^{13}C NMR (126 MHz, CDCl$_3$) anomeric region: δ 108.9 (C-1', **19α**), 98.9 (C-1', **20α**), 97.5 (C-1, **19α**), 97.3 (C-1, **20α**) ppm.

Methyl 2,3,5,6-tetra-*O-tert*-butyldimethylsilyl-β-D-galactofuranosyl-(1→3)-2,6-di-*O*-benzyl-β-D-galactopyranoside (18β) and methyl 2,3,5,6-tetra-*O-tert*-butyldimethylsilyl-β-D-galactofuranosyl-(1→4)-2,6-di-*O*-benzyl-β-D-galactopyranoside (19β)

Obtained according to the general procedure by condensation of **5** with **1β**. The ^{1}H NMR spectrum of the crude mixture evidenced the formation of two disaccharides in a 2.3:1 ratio,

according to the integration of the H-1′ signals. After purification by column chromatography (hexane/EtOAc, 9:1, v/v), fractions of R_f 0.35 (10:1 hexane-EtOAc) afforded compound **18β** (0.05 g, 50%), $[\alpha]_D$ -18 (*c* 1, $CHCl_3$). ^1H NMR (500 MHz, $CDCl_3$): δ 7.38-7.24 (m, 10H, aromatic), 5.10 (s, 1H, H-1'), 4.86, 4.67 (2d, 2H, J_{gem} = 11.2 Hz, CH_2Ph), 4.66, 4.57 (2d, 2H, J_{gem} = 12.0 Hz, CH_2Ph), 4.30 (d, 1H, $J_{1,2}$ = 7.7 Hz, H-1), 4.10-4.06 (m, 3H, H-2', H-3' and H-4), 3.94 (dd, 1H, $J_{3',4'}$ = 2.6, $J_{4',5'}$ = 6.9 Hz, H-4'), 3.81-3.79 (m, 2H, H-6a and H-6b), 3.72 (ddd, 1H, $J_{5',6'a}$ = 5.3, $J_{5',6'b}$ = 5.3, $J_{4',5'}$ = 6.9 Hz, H-5'), 3.69-3.63 (m, 2H, H-5 and H-6’a), 3.62-3.52 (m, 6H, H-2, H-3, H-6’b and CH_3O), 0.89, 0.88, 0.878, 0.871 (4s, 36H, $Si(CH_3)_2C(CH_3)_3$), 0.11, 0.10, 0.08, (3s, 9H, $Si(CH_3)_2C(CH_3)_3$), 0.06-0.04 (m, 15H, $Si(CH_3)_2C(CH_3)_3$) ppm. ^{13}C NMR (126 MHz, $CDCl_3$): δ 138.8, 138.4, 128.3, 128.1, 127.7, 127.6, 127.5, 127.4 (aromatic), 109.1 (C-1'), 104.5 (C-1), 89.1 (C-4'), 82.9 (C-2'), 80.2 (C-3), 78.7 (C-3'), 78.1 (C-2), 74.9 (CH_2Ph), 74.2 (C-5'), 73.68 (C-5), 73.62 (CH_2Ph), 70.0 (C-6), 68.9 (C-4), 65.5 (C-6'), 56.7 (CH_3O), 26.0, 25.9, 25.7, 25.6 ($Si(CH_3)_2C(CH_3)_3$), 18.4, 18.3, 17.86, 17.83 ($Si(CH_3)_2C(CH_3)_3$), -4.2, -4.4, -4.60, -4.62, -4.9, -5.1, -5.3 ($Si(CH_3)_2C(CH_3)_3$) ppm. ESIMS: *m/z* calcd for $C_{51}H_{92}NaO_{11}Si_4$ $[M+Na]^+$ 1015.5609. Found: 1015.5609.

Fractions of *Rf* 0.22 afforded **19β** (0.02 g, 20%), $[\alpha]_D$ -28 (*c* 1, $CHCl_3$). ^1H NMR (500 MHz, $CDCl_3$): δ 7.38-7.27 (m, 10H, aromatic), 5.25 (s, 1H, H-1'), 4.95, 4.62 (2d, 2H, J_{gem} = 11.5 Hz, CH_2Ph), 4.58, 4.56 (2d, 2H, J_{gem} = 12.0 Hz, CH_2Ph), 4.25 (d, 1H, $J_{1,2}$ = 7.6 Hz, H-1), 4.10-4.07 (m, 2H, H-2' and H-3'), 4.03 (dd, 1H, *J* = 0.8, *J* = 1.2 Hz, H-4), 3.94 (dd, 1H, $J_{3',4'}$ = 4.5, $J_{4',5'}$ = 4.5 Hz, H-4'), 3.83-3.74 (m, 3H, H-5', H-6a and H-6b), 3.70 (dd, 1H, $J_{5',6'a}$ = 4.8, J_{gem} = 10.4 Hz, H-6’a), 3.68-3.62 (m, 2H, H-3 and H-5), 3.59 (dd, 1H, $J_{5',6'b}$ = 6.2 Hz, J_{gem} = 10.4 Hz, H-6’b), 3.55 (s, 3H, CH_3O). 3.53 (dd, 1H, J_{1-2} = 7.6 Hz, $J_{2,3}$ = 9.7 Hz, H-2), 0.91-0.89 (m, 27H,

Si(CH$_3$)$_2$C(C*H*$_3$)$_3$). 0.87 (s, 9H, Si(CH$_3$)$_2$C(C*H*$_3$)$_3$), 0.11, 0.09, 0.086, 0.083, 0.07, 0.06, 0.058, 0.051 (8s, 24H, Si(C*H*$_3$)$_2$C(CH$_3$)$_3$) ppm. ^{13}C NMR (126 MHz, CDCl$_3$): δ 138.59, 138.51, 128.5, 128.3, 128.0, 127.8, 127.6, 127.5 (aromatic), 108.8 (C-1'), 104.4 (C-1), 87.2 (C-4'), 83.7 (C-2'), 79.7 (C-3'), 79.5 (C-2), 74.6 (C-5), 74.5 (*C*H$_2$Ph), 74.1 (C-5'), 74.0 (C-3), 73.6 (*C*H$_2$Ph), 71.9 (C-4), 70.7 (C-6), 65.6 (C-6'), 56.6 (*C*H$_3$O), 26.0, 25.8, 25.7 (Si(CH$_3$)$_2$C(*C*H$_3$)$_3$), 18.4, 18.3, 17.9, 17.8 (Si(CH$_3$)$_2$*C*(CH$_3$)$_3$), -3.9, -4.0, -4.34, -4.39, -4.4, -4.7, -5.1, -5.2 (Si(*C*H$_3$)$_2$C(CH$_3$)$_3$) ppm. ESIMS: *m*/*z* calcd for $C_{51}H_{92}NaO_{11}Si_4$ $[M+Na]^+$ 1015.5609. Found: 1015.5615.

Computational methods

Molecular mechanics calculations were carried out using the program MM3(92) (QCPE, Indiana, USA) [6,7], with relevant parameters modified as in the MM3(2000) version [8]. Also, the maximum atomic movement at each step of minimization was reduced from 0.25 to 0.10 Å. The structures were minimized using the block diagonal method, with a termination condition 100 times tighter than the MM3 default. Quantum mechanical calculations were carried out using Gaussian 09W (rev. C.01) [9], with standard termination options.

From a given rotamer, an automated routine was used to generate the starting conformations produced by rotation of ±120º for each of the exocyclic dihedrals. Those conformers within the first 10 kcal/mol were submitted to DFT optimizations at the B3LYP/6-311+G** level and then to single point calculations with M06-2X at the same level. Charges were then obtained with both functionals at the same level of theory, for both the ground molecule and the radical cation, using the Merz–Kollmann scheme [10,11]. Condensed-to-atom Fukui functions were then obtained as

Boltzmann average ratios following both Yang and Mortier [12] and Contreras and co-workers' [13] methods.

Stationary points were characterized by frequency calculations in order to verify that the minima had no imaginary frequencies. Population analysis was carried out using the Boltzmann equation, with a temperature of 298 K and using electronic energies for DFT.

References

1. Schneider, J.; Lee, Y. C.; Flowers, H. M. *Carbohydr. Res.* **1974**, *36*, 159–166.
2. Flowers, H. M. *Carbohydr. Res.* **1975**, *39*, 245–251.
3. Wegmann, B.; Schmidt, R. R. *J. Carbohydr. Chem.* **1987**, *6*, 357–375.
4. Gallo-Rodriguez, C.; Gandolfi, L.; De Lederkremer, R. M. *Org. Lett.* **1999**, *1*, 245–247.
5. Lubineau, A.; Basset-Carpentier, K.; Augé, C. *Carbohydr. Res.* **1997**, *300*, 161–167.
6. Allinger, N. L.; Yuh, Y. H.; Lii, J. H. *J. Am. Chem. Soc.* **1989**, *111*, 8551–8566.
7. Allinger, N. L.; Rahman, M.; Lii, J. H. *J. Am. Chem. Soc.* **1990**, *112*, 8293–8307.
8. Stortz, C. A. *J. Comput. Chem.* **2005**, *26*, 471–483.
9. Gaussian 09, Revision C.01; Gaussian, Inc.: Wallingford, CT, 2009.
10. Singh, U. C.; Kollman, P. A. *J. Comput. Chem.* **1984**, *5*, 129–145.
11. Besler, B. H.; Merz, K. M.; Kollman, P. A. *J. Comput. Chem.* **1990**, *11*, 431–439.
12. Yang, W.; Mortier, W. J. *J. Am. Chem. Soc.* **1986**, *108*, 5708–5711.
13. Contreras, R. R.; Fuentealba, P.; Galván, M.; Pérez, P. *Chem. Phys. Lett.* **1999**, *304*, 405–413.

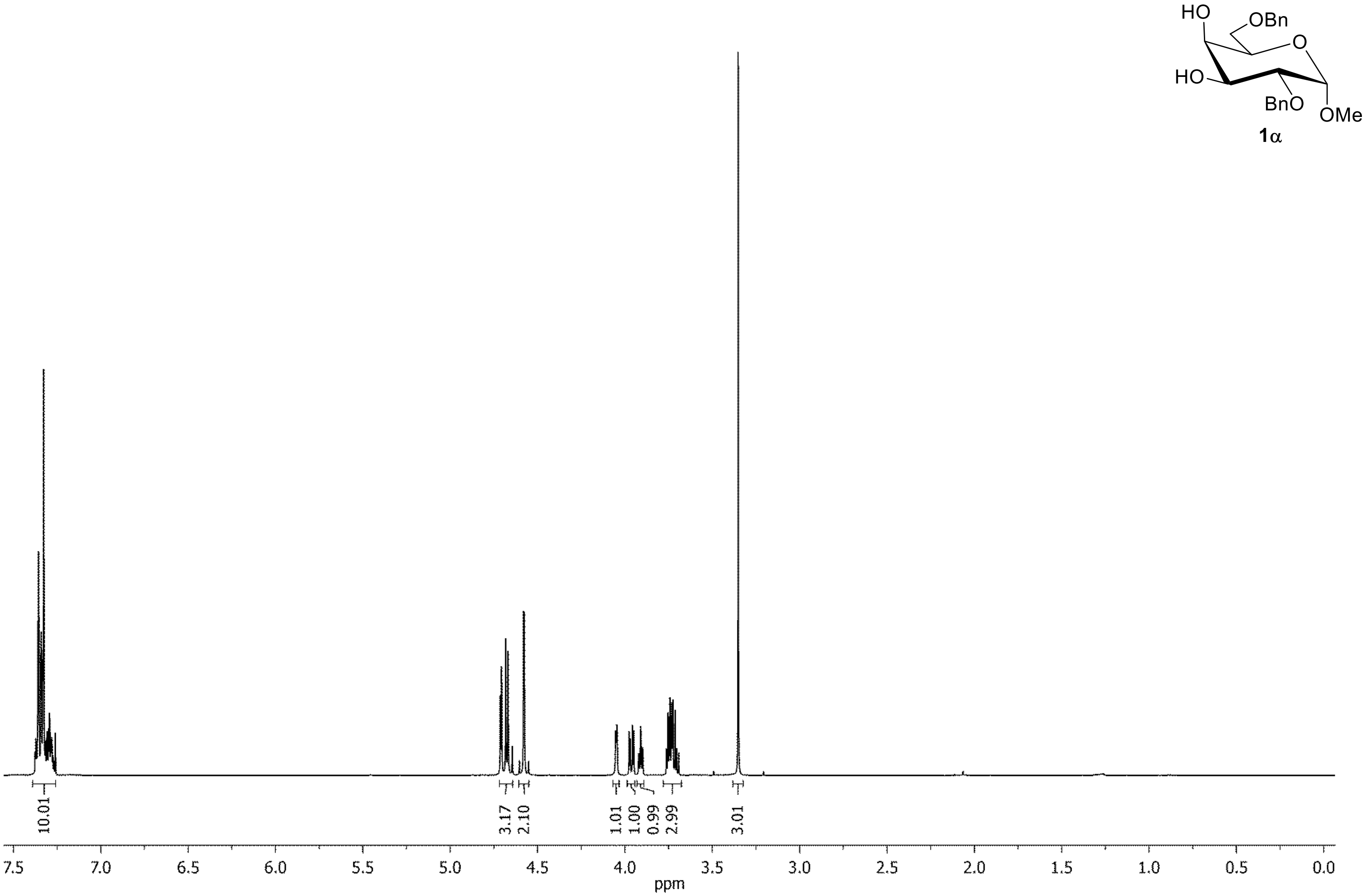
HO
OBn
O
HO
BnO
OMe
10.01
3.17
2.10
1.01
1.00
0.99
2.99
3.01
7.5
7.0
6.5
6.0
5.5
5.0
4.5
4.0
3.5
3.0
2.5
2.0
1.5
1.0
0.5
0.0
ppm

1α

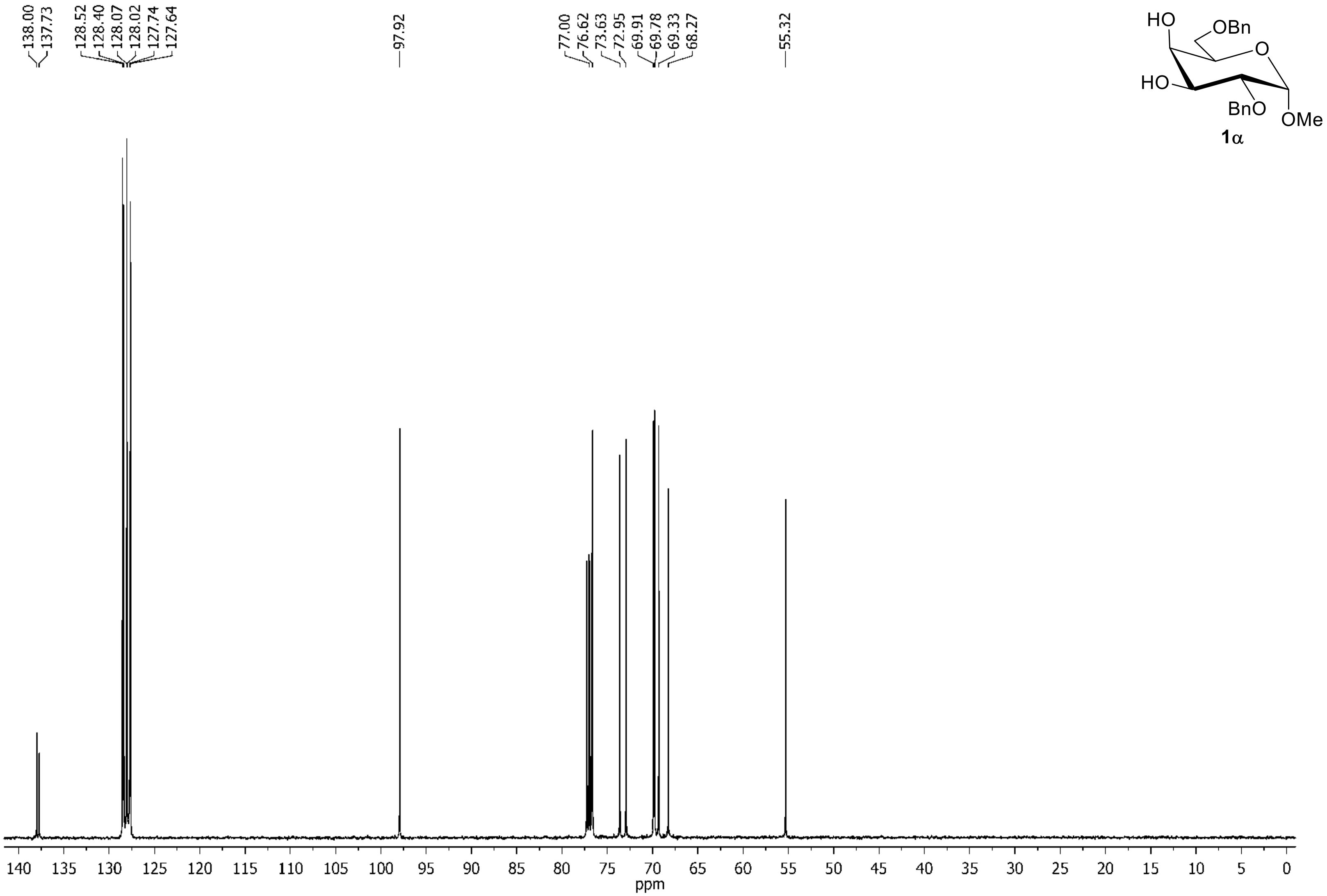
138.00
137.73
128.52
128.40
128.07
128.02
127.74
127.64
97.92
77.00
76.62
73.63
72.95
69.91
69.78
69.33
68.27
55.32
HO
OBn
O
HO
BnO
OMe
1α
140 135 130 125 120 115 110 105 100 95 90 85 80 75 70 65 60 55 50 45 40 35 30 25 20 15 10 5 0
ppm

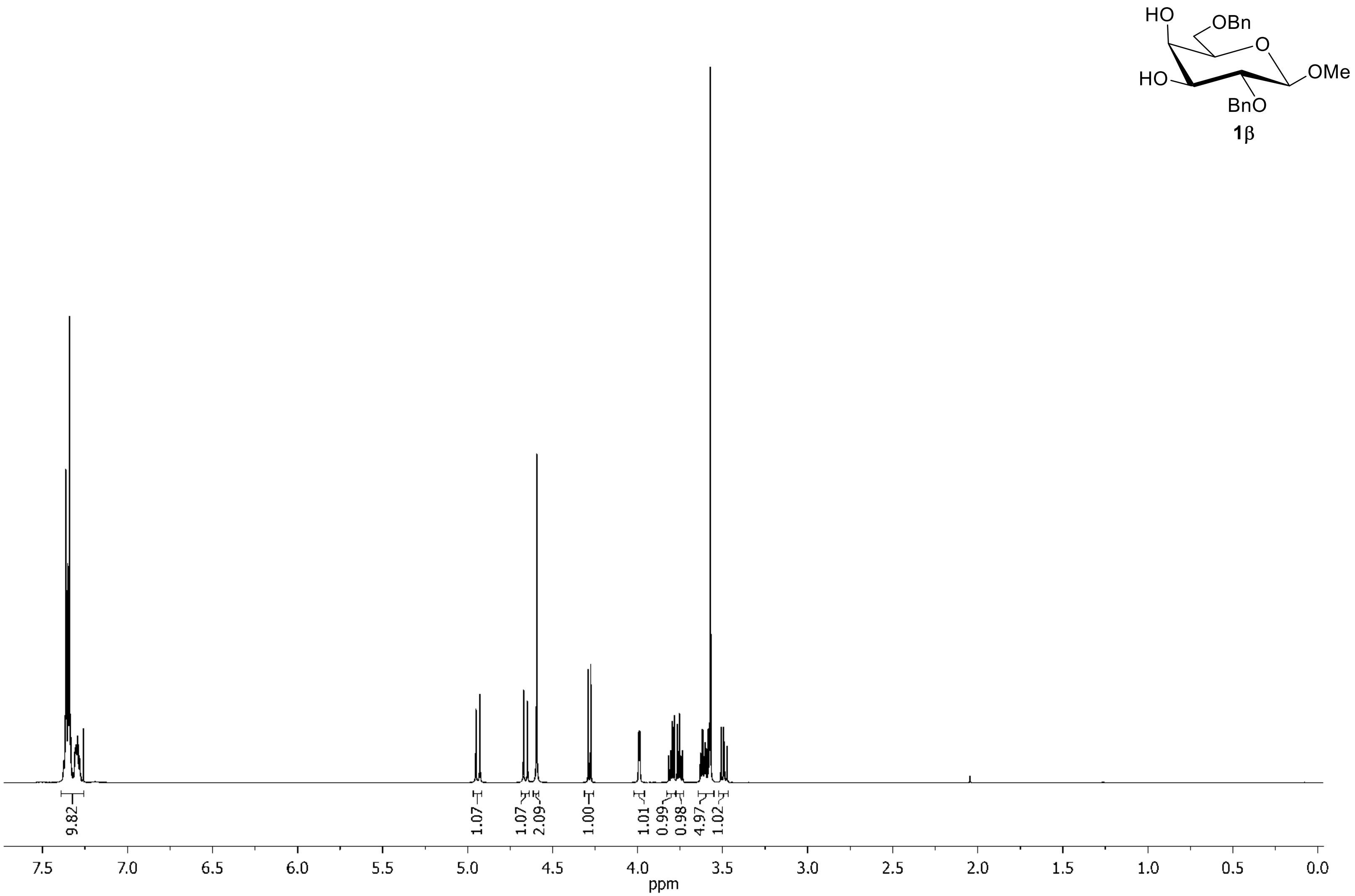
HO
OBn
O
OMe
HO
BnO
1β
9.82
1.07
1.07
2.09
1.00
1.01
0.99
0.98
4.97
1.02
7.5
7.0
6.5
6.0
5.5
5.0
4.5
4.0
3.5
3.0
2.5
2.0
1.5
1.0
0.5
0.0
ppm

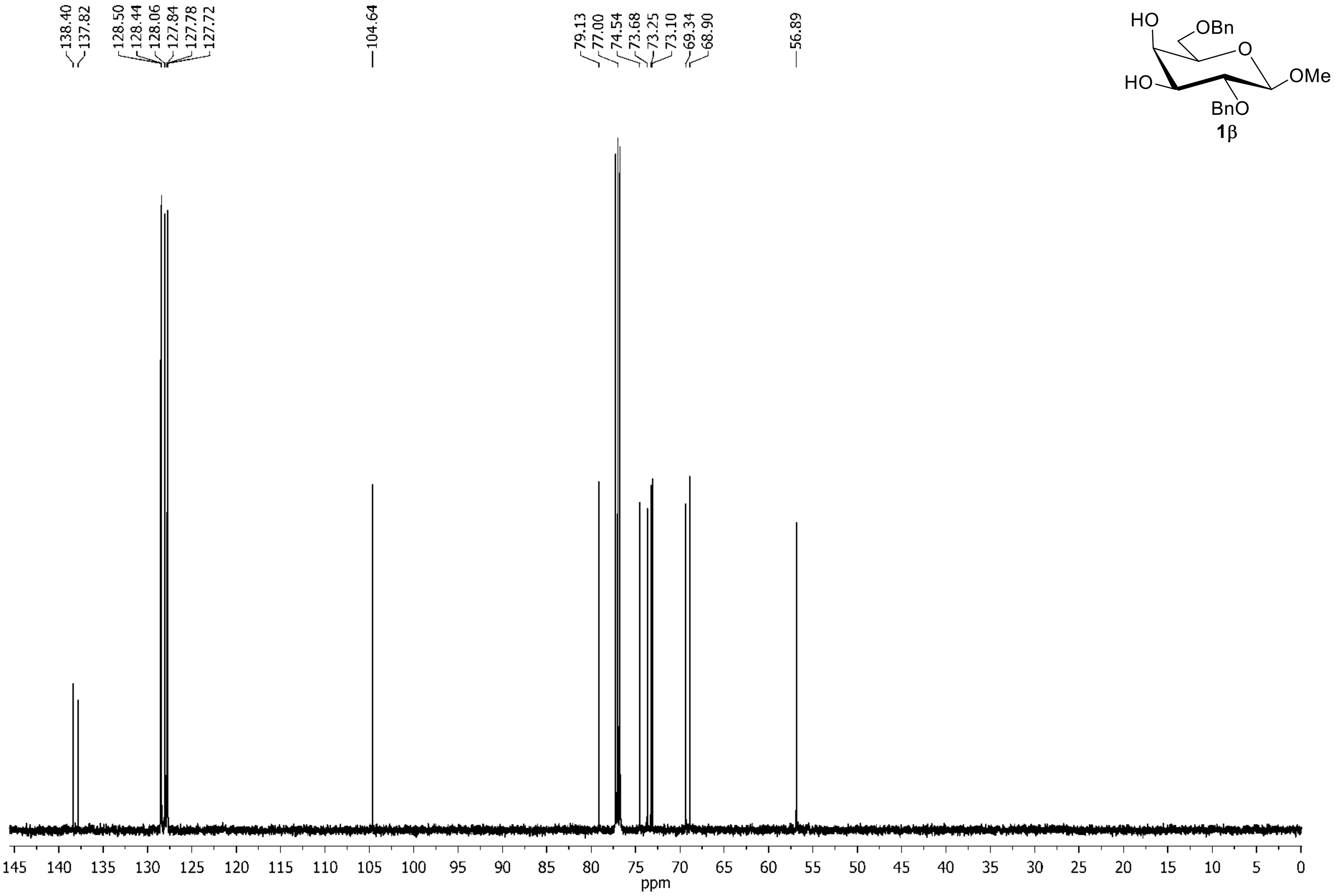
138.40
137.82
128.50
128.44
128.06
127.84
127.78
127.72
104.64
79.13
77.00
74.54
73.68
73.25
73.10
69.34
68.90
56.89
HO
OBn
O
OMe
HO
BnO
1β
145 140 135 130 125 120 115 110 105 100 95 90 85 80 75 70 65 60 55 50 45 40 35 30 25 20 15 10 5 0
ppm

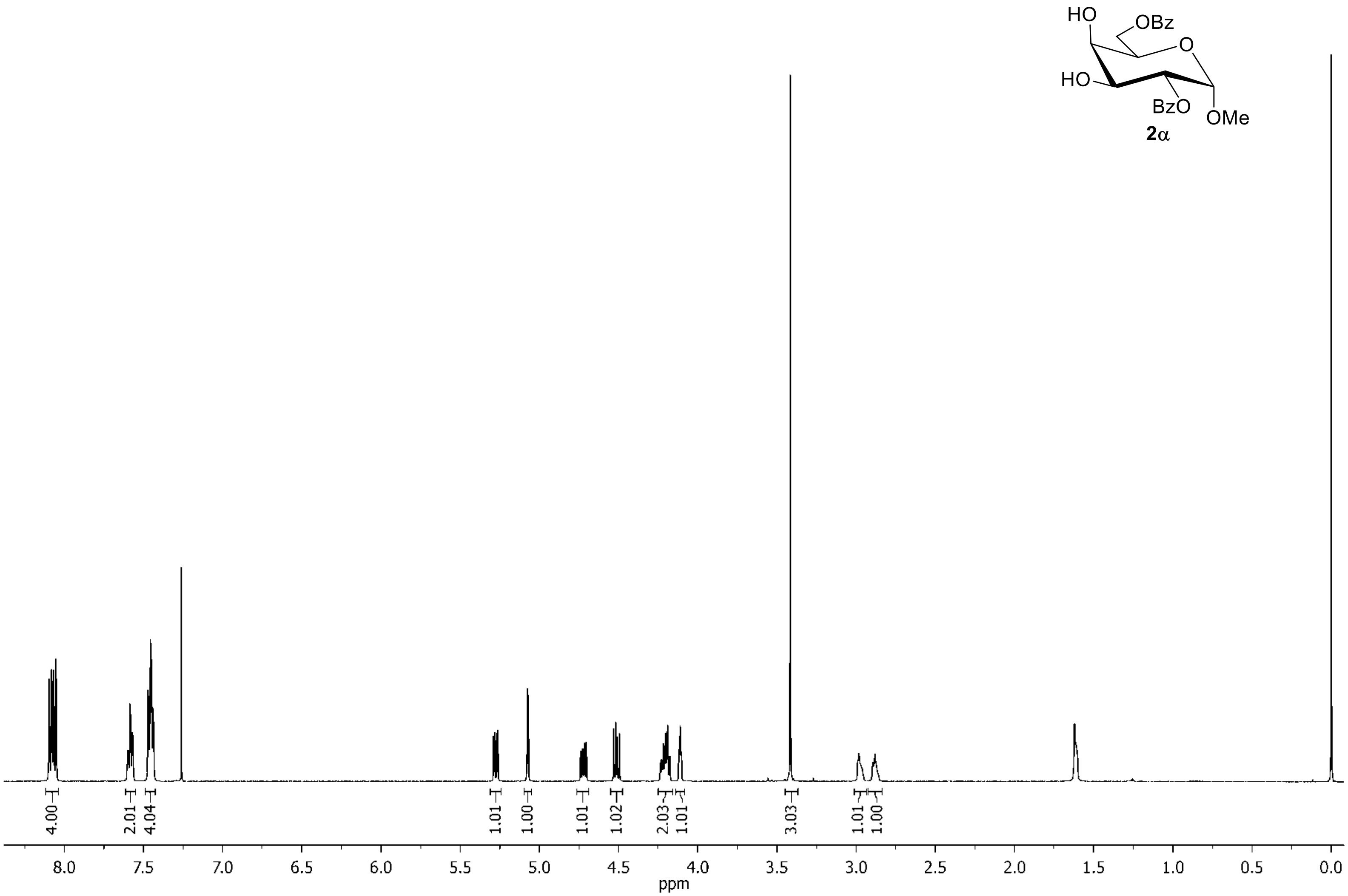

HO
OBz
O
HO
BzO
OMe
2α
4.00
2.01
4.04
1.01
1.00
1.01
1.02
2.03
1.01
3.03
1.01
1.00
8.0
7.5
7.0
6.5
6.0
5.5
5.0
4.5
4.0
3.5
3.0
2.5
2.0
1.5
1.0
0.5
0.0
ppm

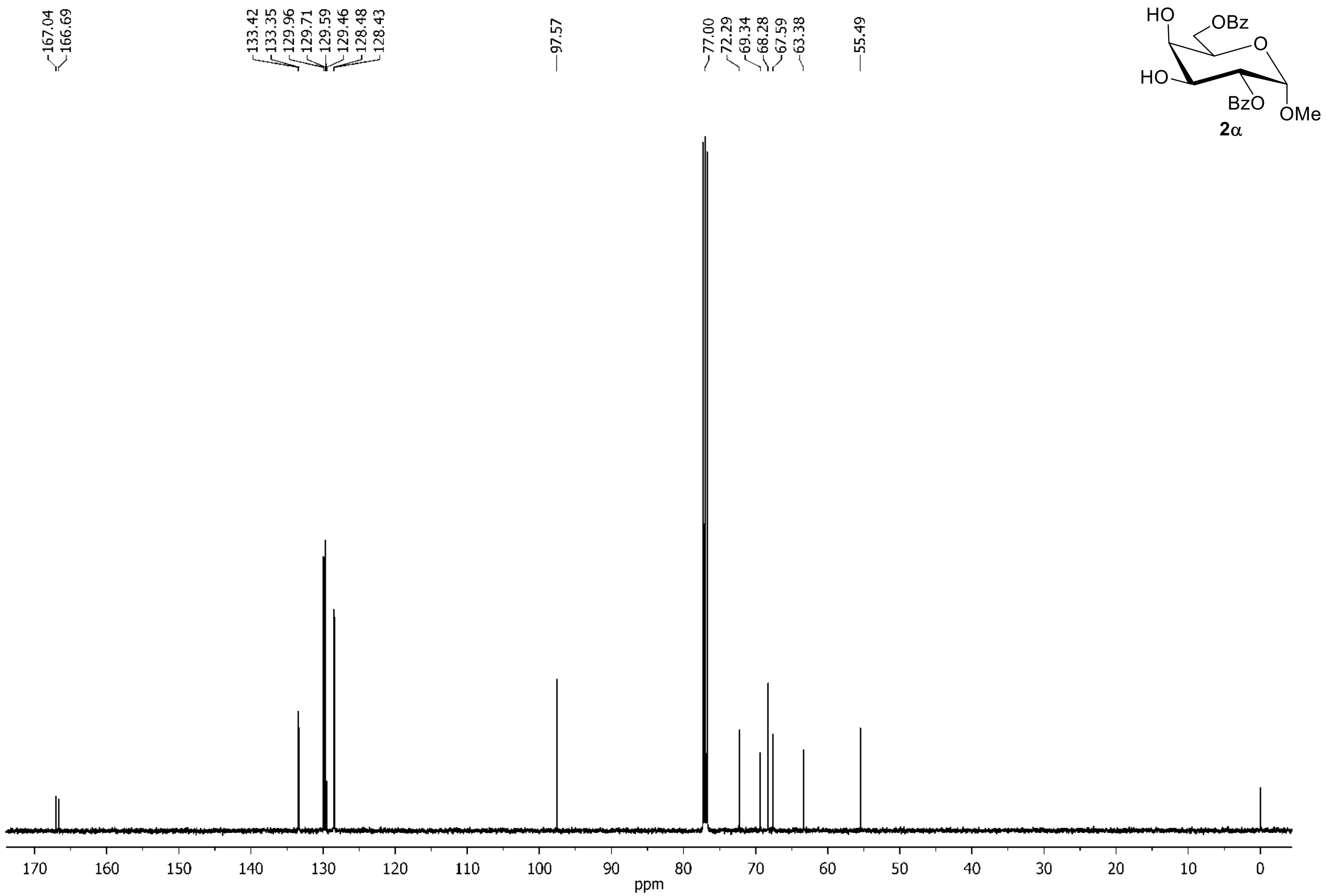
167.04
166.69
133.42
133.35
129.96
129.71
129.59
129.46
128.48
128.43
97.57
77.00
72.29
69.34
68.28
67.59
63.38
55.49
HO
OBz
O
HO
BzO
OMe
2α
170
160
150
140
130
120
110
100
90
80
70
60
50
40
30
20
10
0
ppm

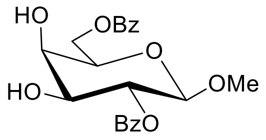
HO
OBz
O
OMe
HO
BzO

2β

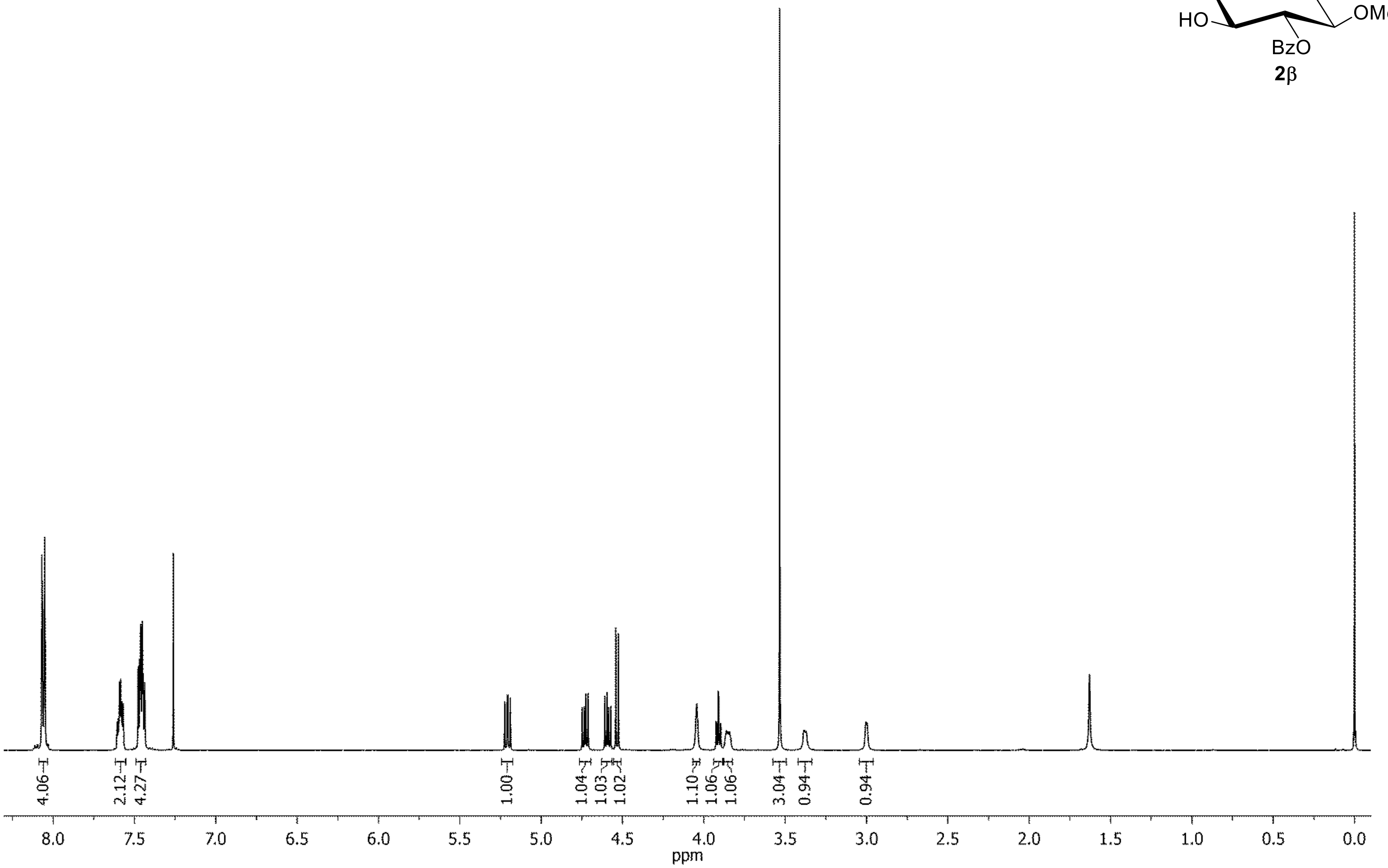
4.06
2.12
4.27
1.00
1.04
1.03
1.02
1.10
1.06
1.06
3.04
0.94
0.94
8.0
7.5
7.0
6.5
6.0
5.5
5.0
4.5
4.0
3.5
3.0
2.5
2.0
1.5
1.0
0.5
0.0
ppm

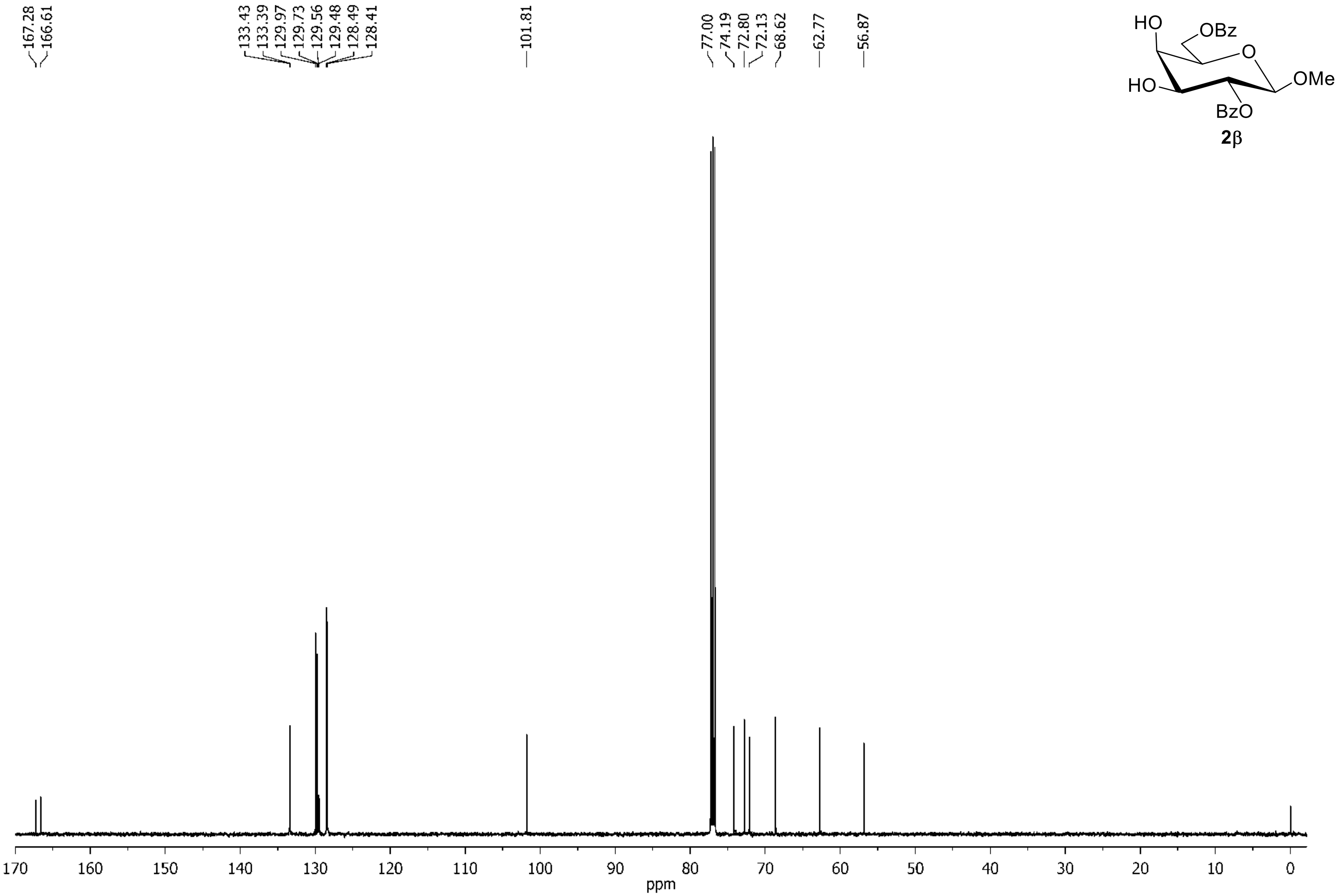

167.28
166.61
133.43
133.39
129.97
129.73
129.56
129.48
128.49
128.41
101.81
77.00
74.19
72.80
72.13
68.62
62.77
56.87
170
160
150
140
130
120
110
100
90
80
70
60
50
40
30
20
10
0
ppm
HO
OBz
O
OMe
HO
BzO

2β

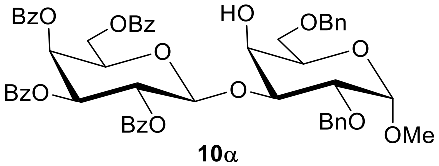
BzO
OBz
O
BzO
BzO
O
HO
OBn
O
BnO
OMe
10α

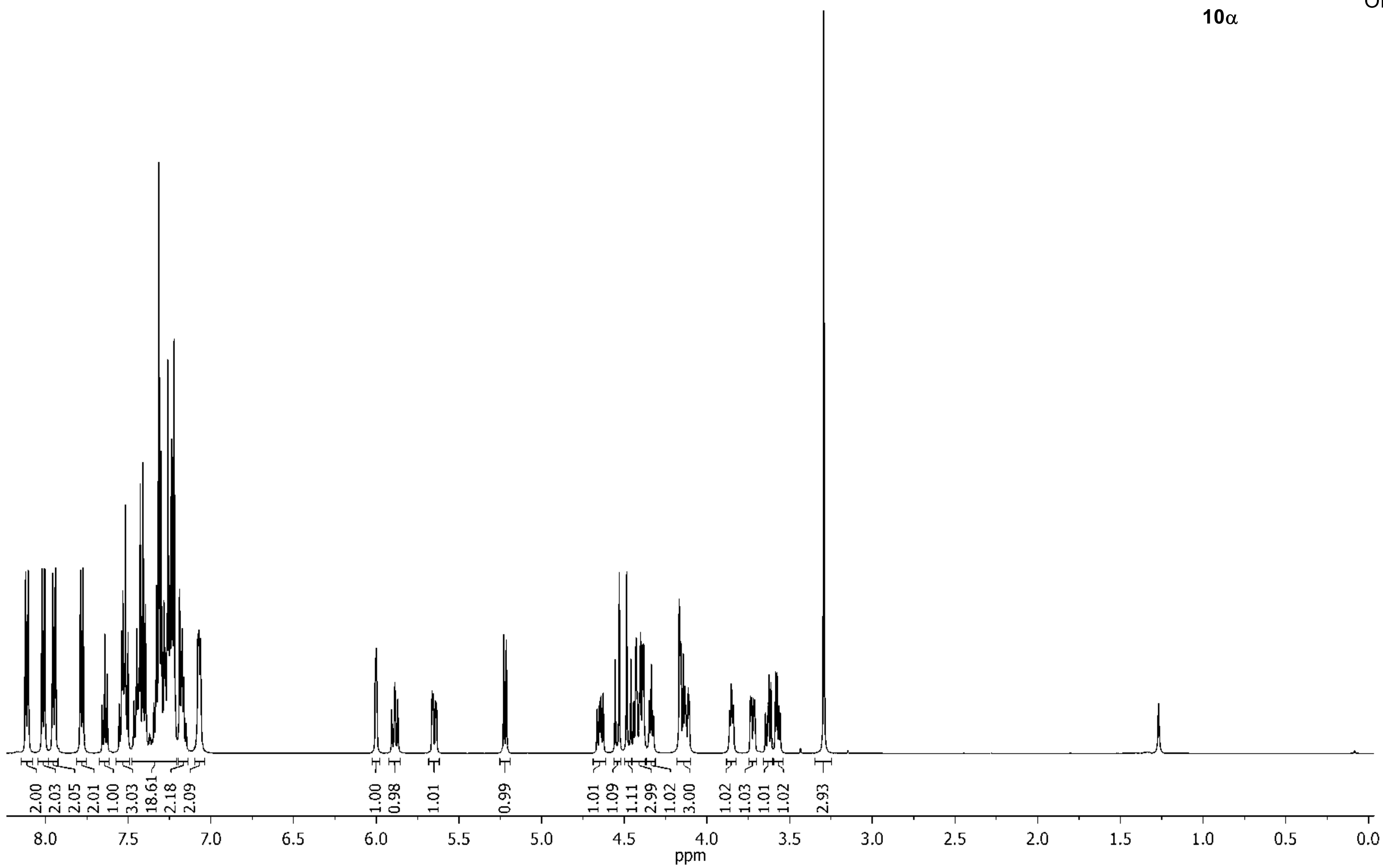
2.00
2.03
2.05
2.01
1.00
3.03
18.61
2.18
2.09
1.00
0.98
1.01
0.99
1.01
1.09
1.11
2.99
1.02
3.00
1.02
1.03
1.01
1.02
2.93
8.0
7.5
7.0
6.5
6.0
5.5
5.0
4.5
4.0
3.5
3.0
2.5
2.0
1.5
1.0
0.5
0.0
ppm

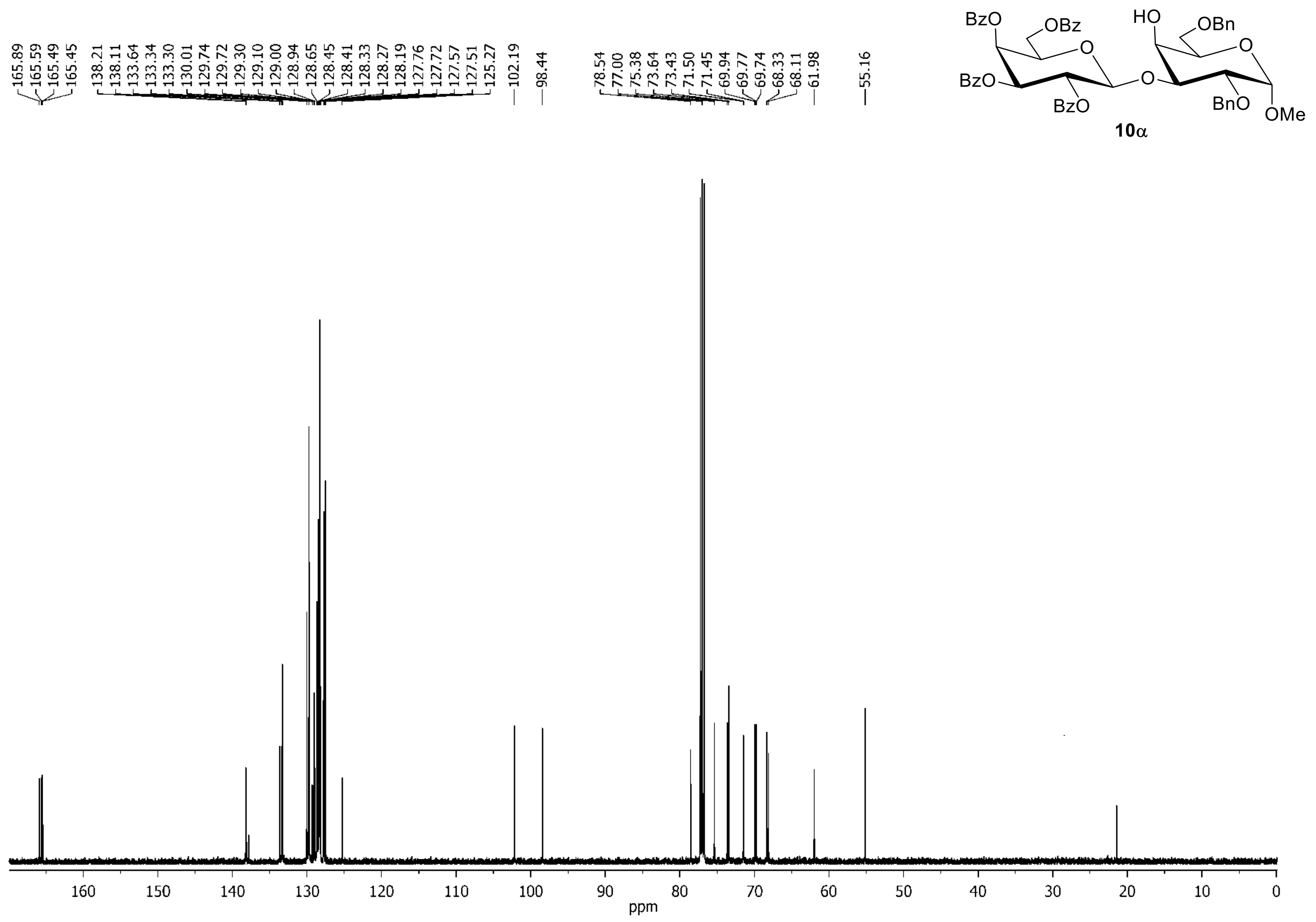
165.89
165.59
165.49
165.45
138.21
138.11
133.64
133.34
133.30
130.01
129.74
129.72
129.30
129.10
129.00
128.94
128.65
128.45
128.41
128.33
128.27
128.19
127.76
127.72
127.57
127.51
125.27
102.19
98.44
78.54
77.00
75.38
73.64
73.43
71.50
71.45
69.94
69.77
69.74
68.33
68.11
61.98
55.16
BzO
OBz
HO
OBn
BzO
BzO
BnO
OMe
160
150
140
130
120
110
100
90
80
70
60
50
40
30
20
10
0
ppm

10α

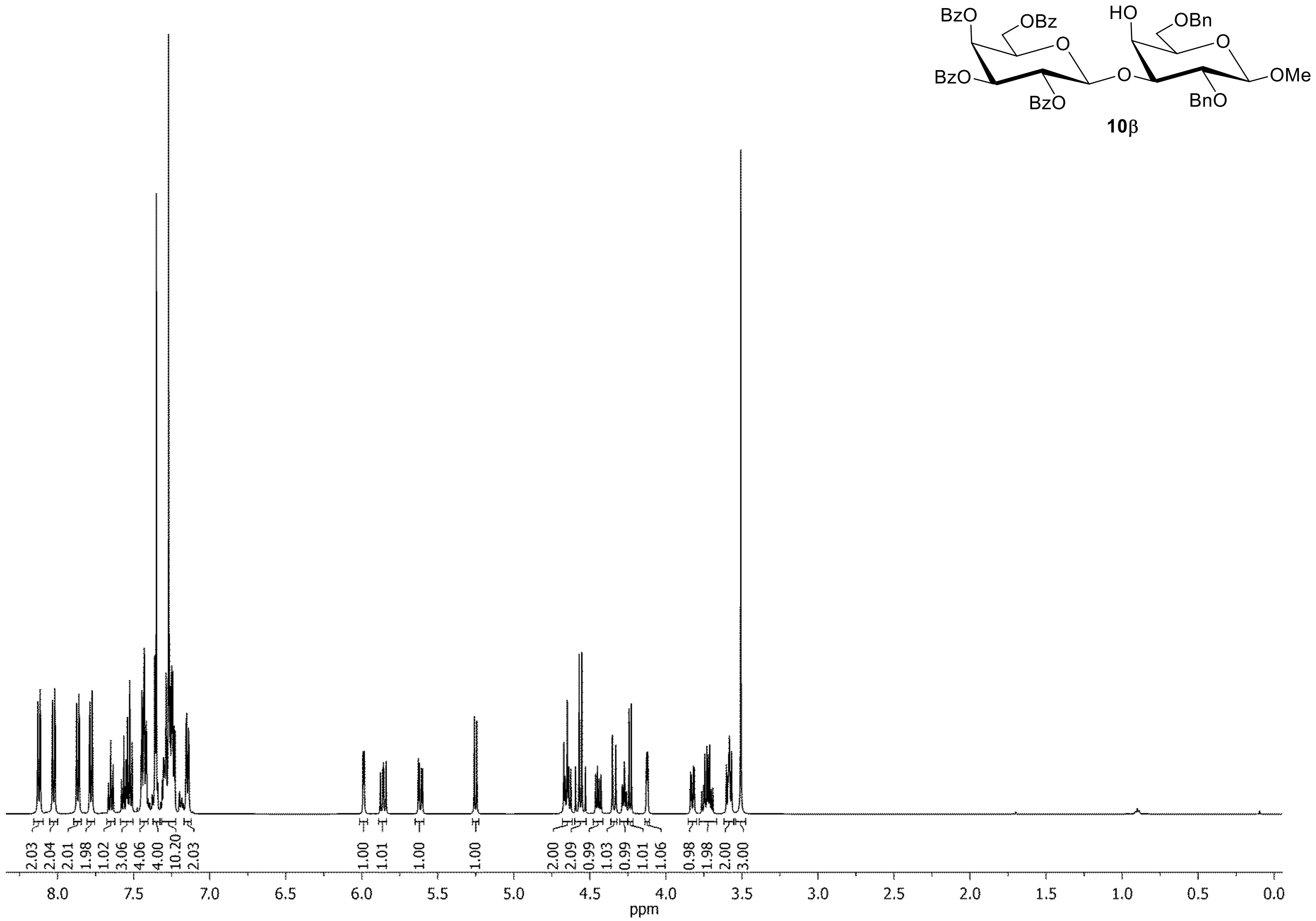

BzO
OBz
BzO
BzO
HO
OBn
OMe
BnO
10β
2.03
2.04
2.01
1.98
1.02
3.06
4.06
4.00
10.20
2.03
1.00
1.01
1.00
1.00
2.00
2.09
0.99
1.03
0.99
1.01
1.06
0.98
1.98
2.00
3.00
8.0
7.5
7.0
6.5
6.0
5.5
5.0
4.5
4.0
3.5
3.0
2.5
2.0
1.5
1.0
0.5
0.0
ppm

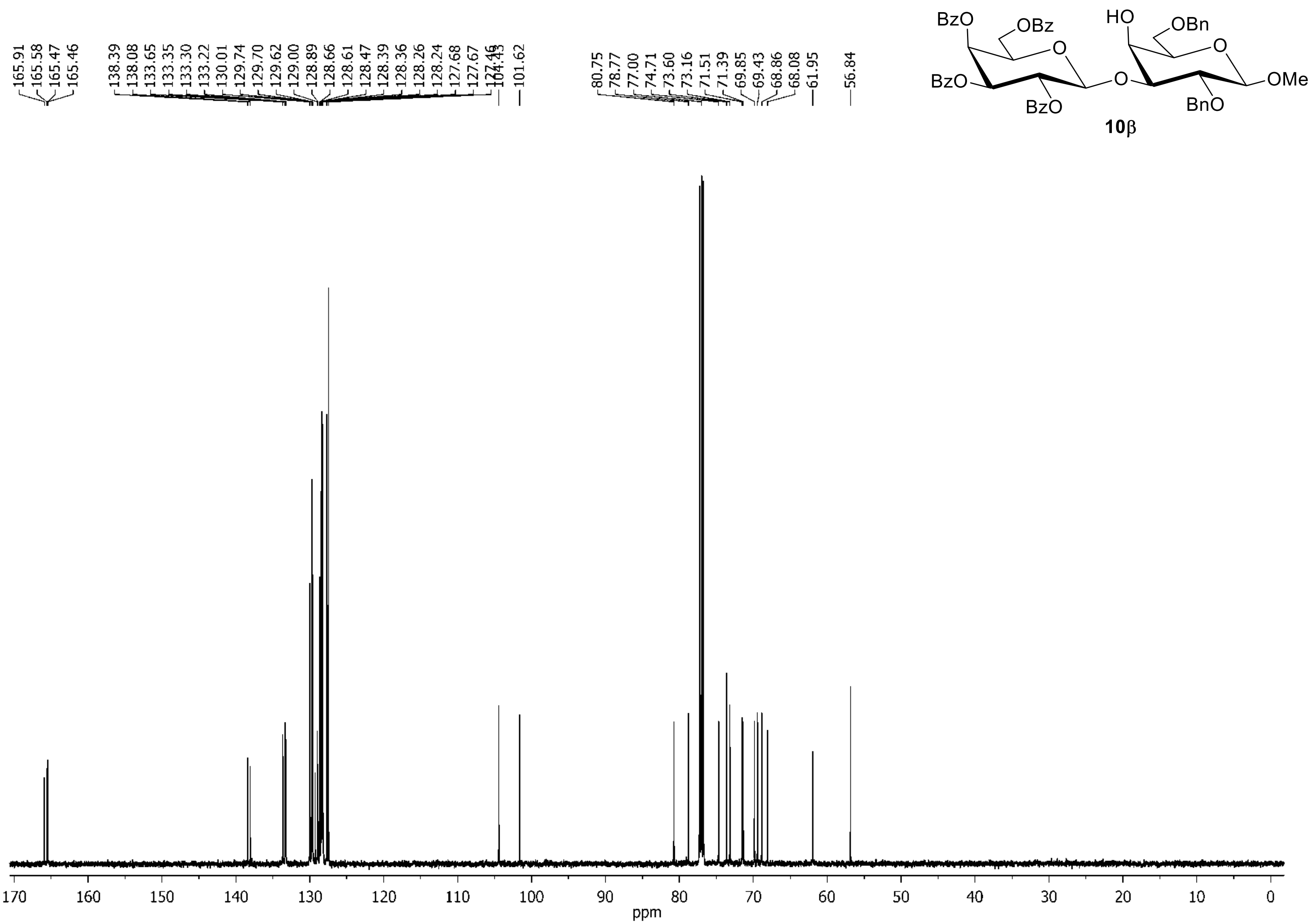
165.91
165.58
165.47
165.46
138.39
138.08
133.65
133.35
133.30
133.22
130.01
129.74
129.70
129.62
129.00
128.89
128.66
128.61
128.47
128.39
128.36
128.26
128.24
127.68
127.67
127.46
104.43
101.62
80.75
78.77
77.00
74.71
73.60
73.16
71.51
71.39
69.85
69.43
68.86
68.08
61.95
56.84
BzO
OBz
O
BzO
BzO
O
HO
OBn
O
OMe
BnO
10β
170
160
150
140
130
120
110
100
90
80
70
60
50
40
30
20
10
0
ppm

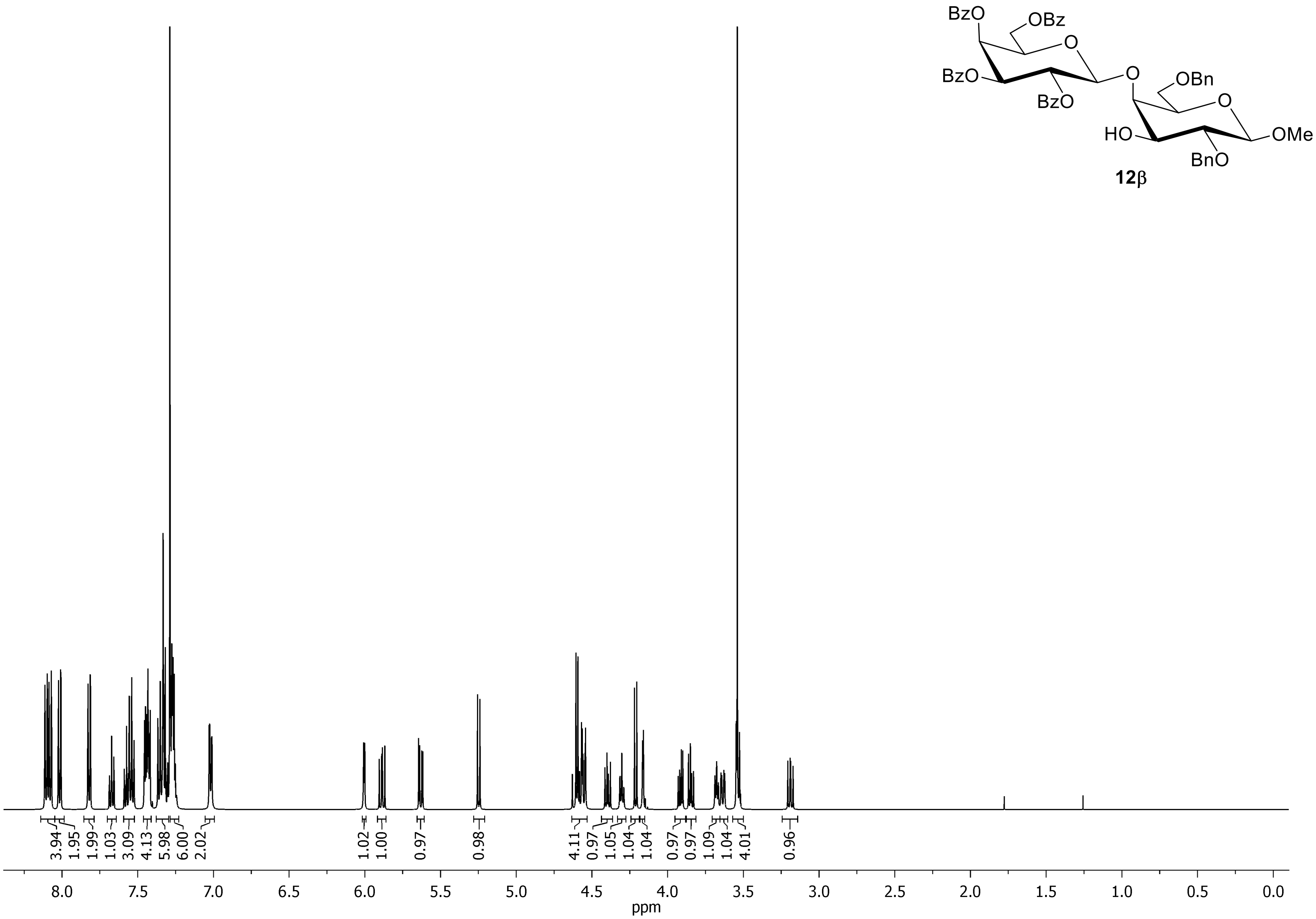
BzO
OBz
O
BzO
BzO
O
OBn
O
HO
OMe
BnO
3.94
1.95
1.99
1.03
3.09
4.13
5.98
6.00
2.02
1.02
1.00
0.97
0.98
4.11
0.97
1.05
1.04
1.04
0.97
0.97
1.09
1.04
4.01
0.96
8.0
7.5
7.0
6.5
6.0
5.5
5.0
4.5
4.0
3.5
3.0
2.5
2.0
1.5
1.0
0.5
0.0
ppm

12β

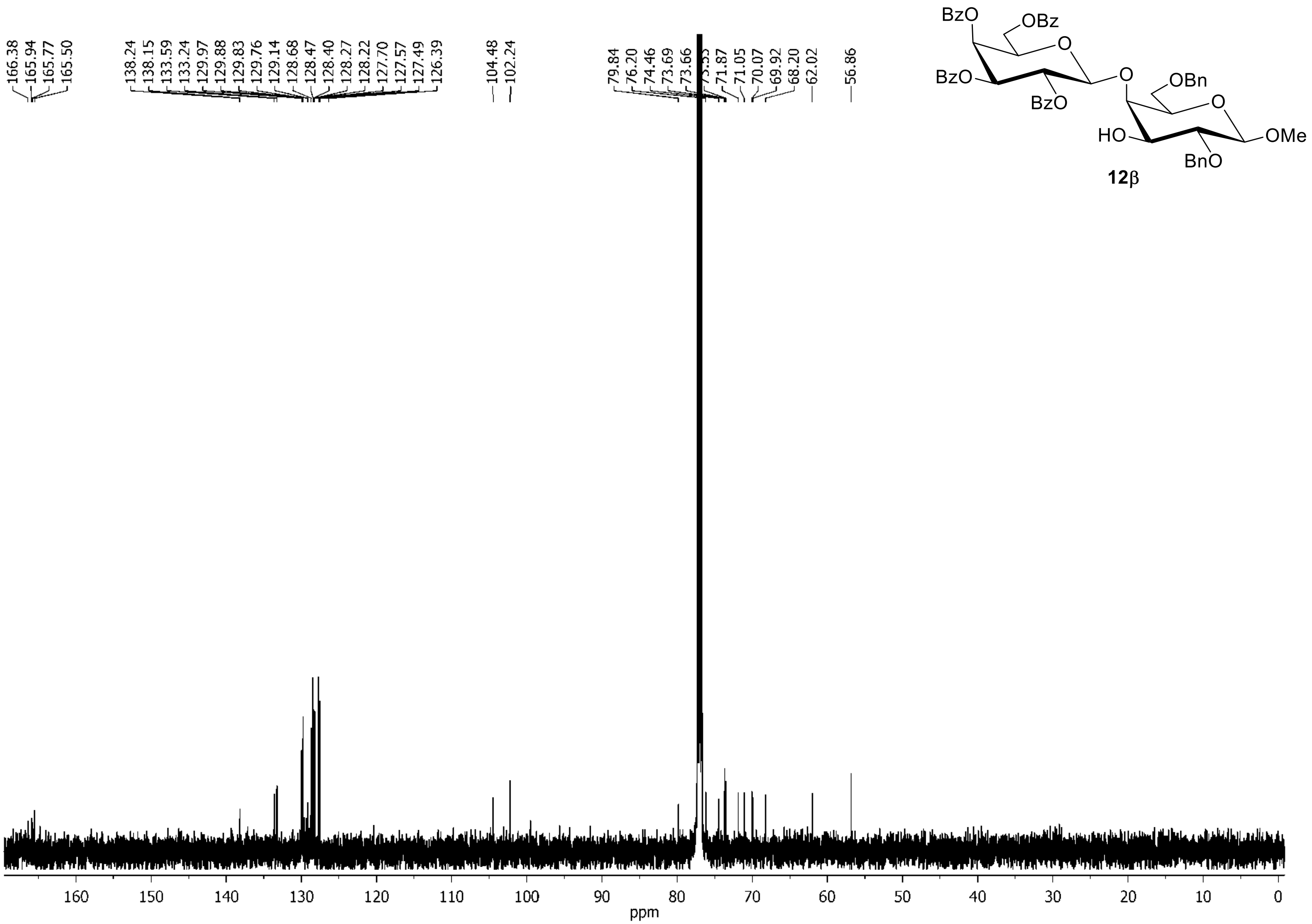
166.38
165.94
165.77
165.50
138.24
138.15
133.59
133.24
129.97
129.88
129.83
129.76
129.14
128.68
128.47
128.40
128.27
128.22
127.70
127.57
127.49
126.39
104.48
102.24
79.84
76.20
74.46
73.69
73.66
71.87
71.05
70.07
69.92
68.20
62.02
56.86
160
150
140
130
120
110
100
90
80
70
60
50
40
30
20
10
0
ppm
BzO
OBz
O
BzO
BzO
O
OBn
O
HO
OMe
BnO

12β

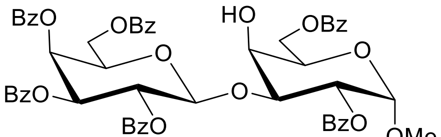
BzO
OBz
BzO
BzO
HO
OBz
BzO
OMe

11α

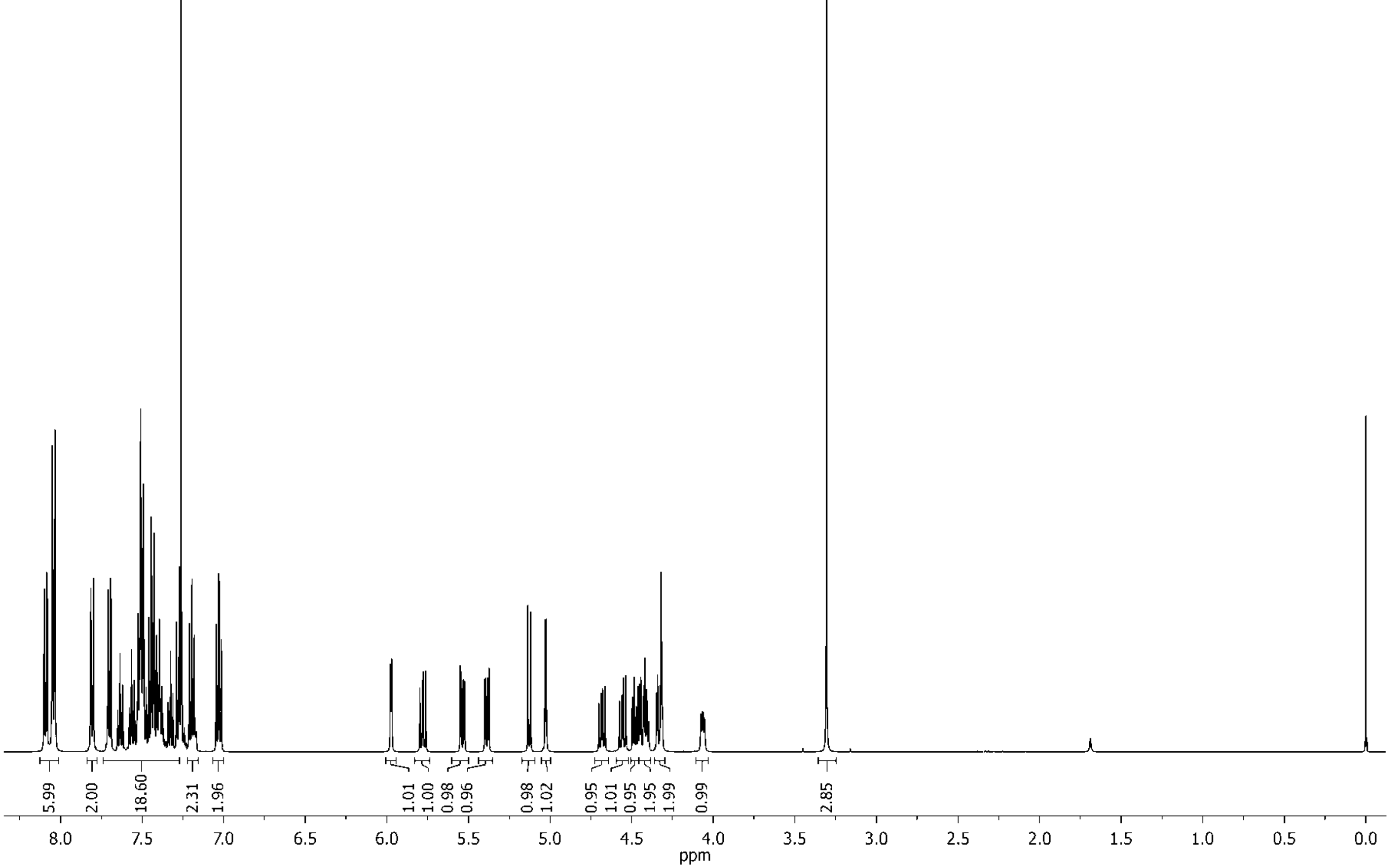
5.99
2.00
18.60
2.31
1.96
1.01
1.00
0.98
0.96
0.98
1.02
0.95
1.01
0.95
1.95
1.99
0.99
2.85
8.0
7.5
7.0
6.5
6.0
5.5
5.0
4.5
4.0
3.5
3.0
2.5
2.0
1.5
1.0
0.5
0.0
ppm

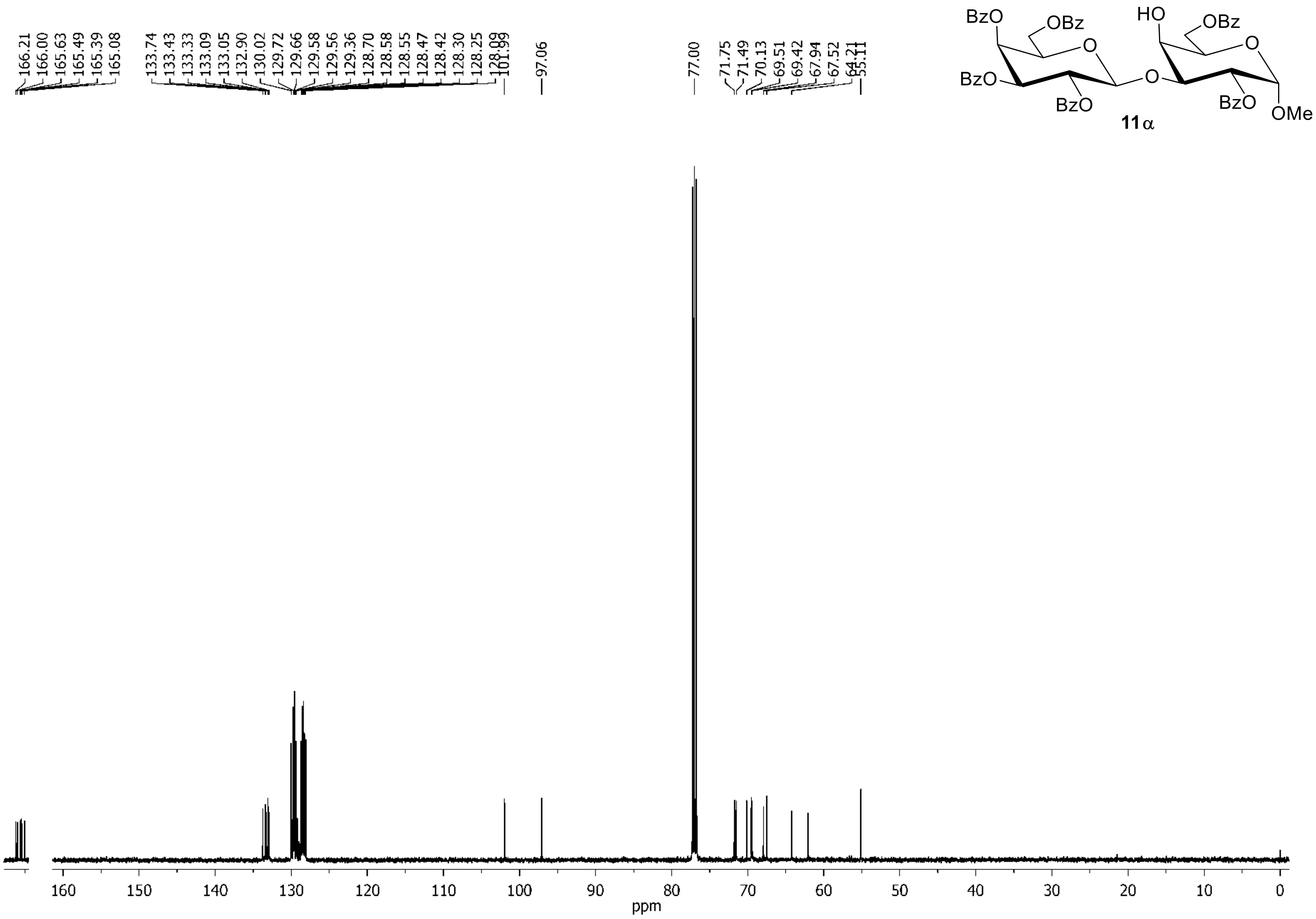

166.21
166.00
165.63
165.49
165.39
165.08
133.74
133.43
133.33
133.09
133.05
132.90
130.02
129.72
129.66
129.58
129.56
129.36
128.70
128.58
128.55
128.47
128.42
128.30
128.25
128.09
101.99
97.06
77.00
71.75
71.49
70.13
69.51
69.42
67.94
67.52
64.21
55.11
160
150
140
130
120
110
100
90
80
70
60
50
40
30
20
10
0
ppm
BzO
OBz
O
HO
OBz
O
BzO
O
BzO
BzO
OMe

11α

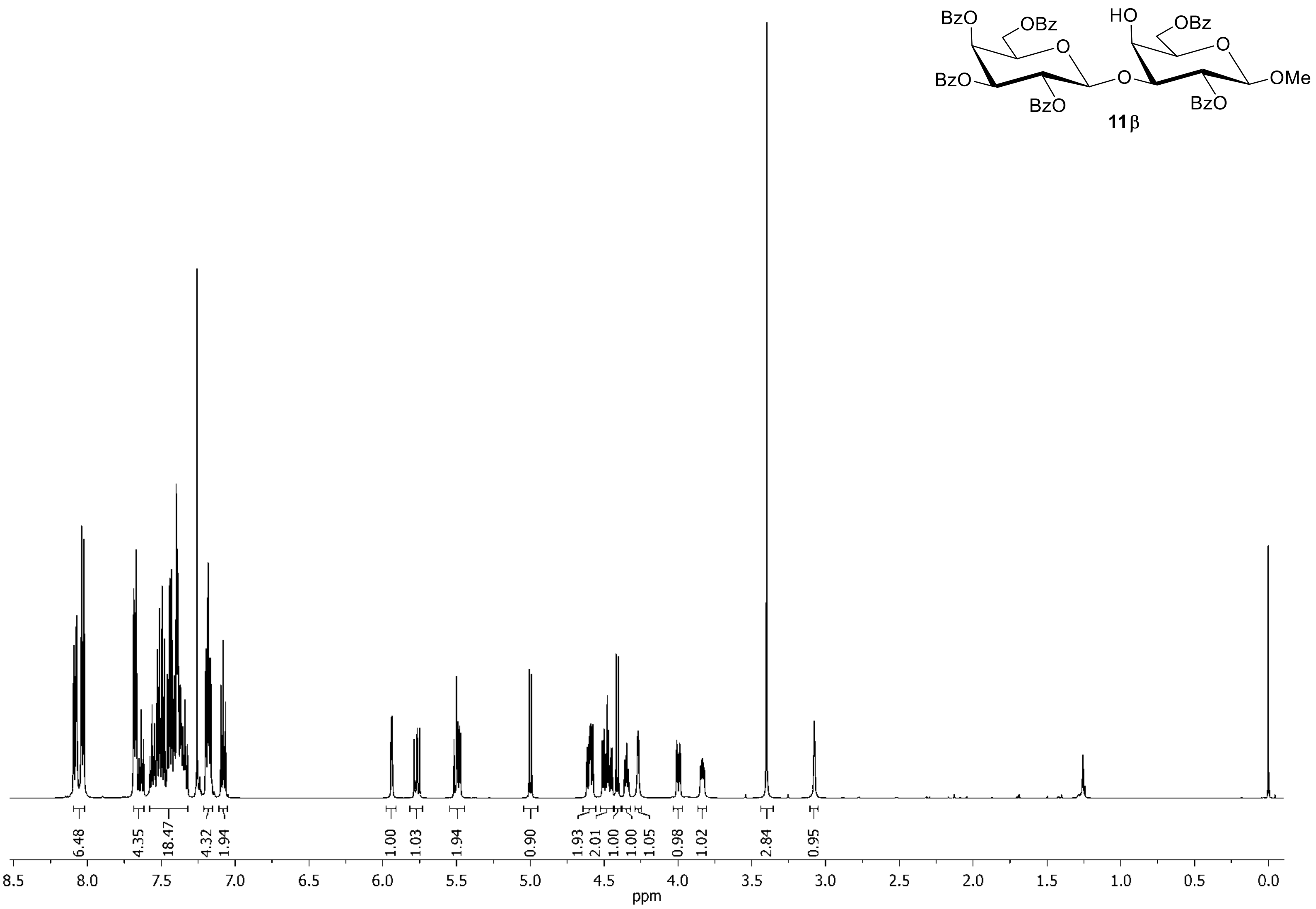

BzO
OBz
O
BzO
BzO
O
HO
OBz
O
OMe
BzO
11β
6.48
4.35
18.47
4.32
1.94
1.00
1.03
1.94
0.90
1.93
2.01
1.00
1.00
1.05
0.98
1.02
2.84
0.95
8.5
8.0
7.5
7.0
6.5
6.0
5.5
5.0
4.5
4.0
3.5
3.0
2.5
2.0
1.5
1.0
0.5
0.0
ppm

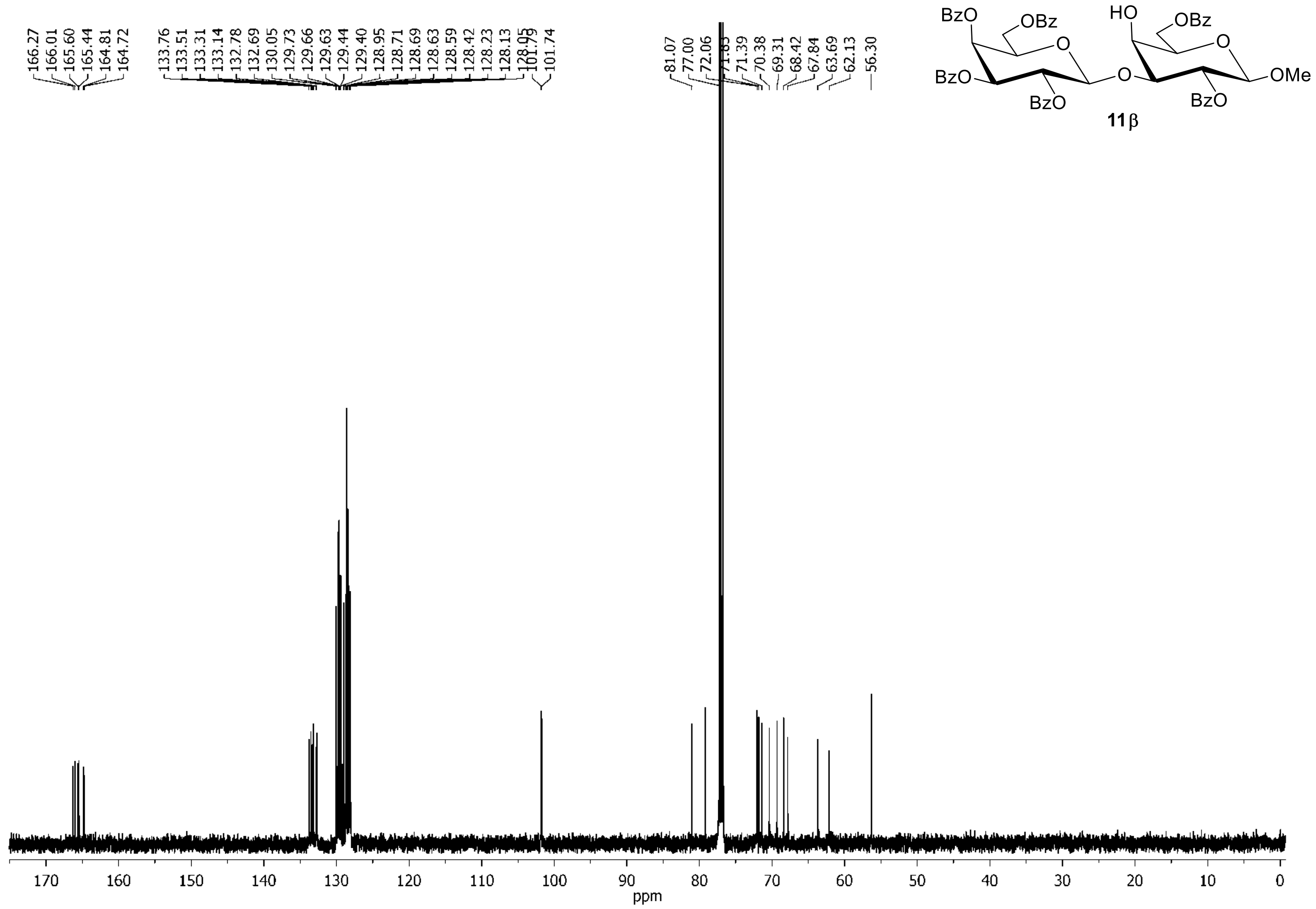

11β

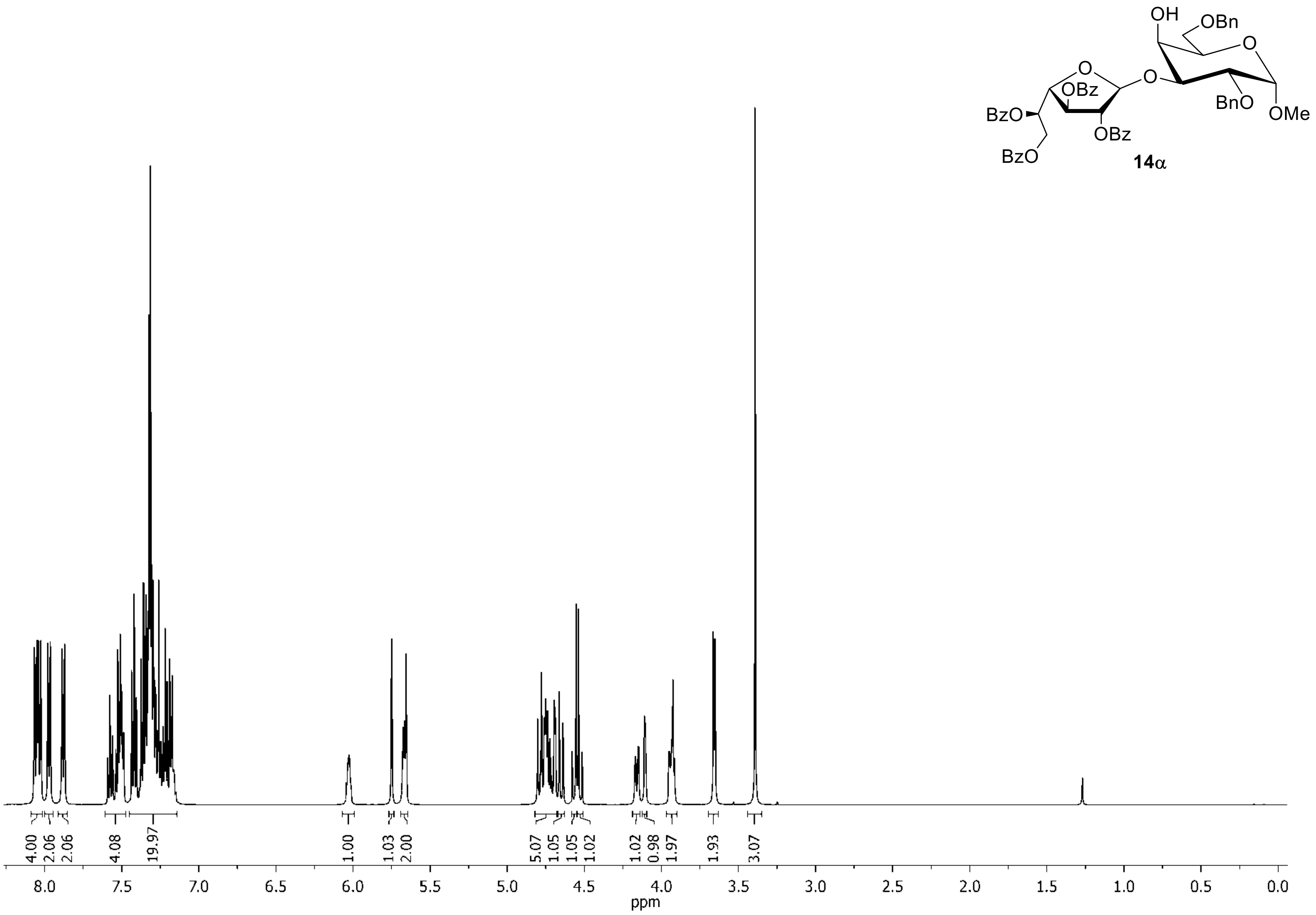
OH
OBn
O
O
OBz
BzO
OBz
BzO
BnO
OMe
14α
4.00
2.06
2.06
4.08
19.97
1.00
1.03
2.00
5.07
1.05
1.05
1.02
1.02
0.98
1.97
1.93
3.07
8.0
7.5
7.0
6.5
6.0
5.5
5.0
4.5
4.0
3.5
3.0
2.5
2.0
1.5
1.0
0.5
0.0
ppm

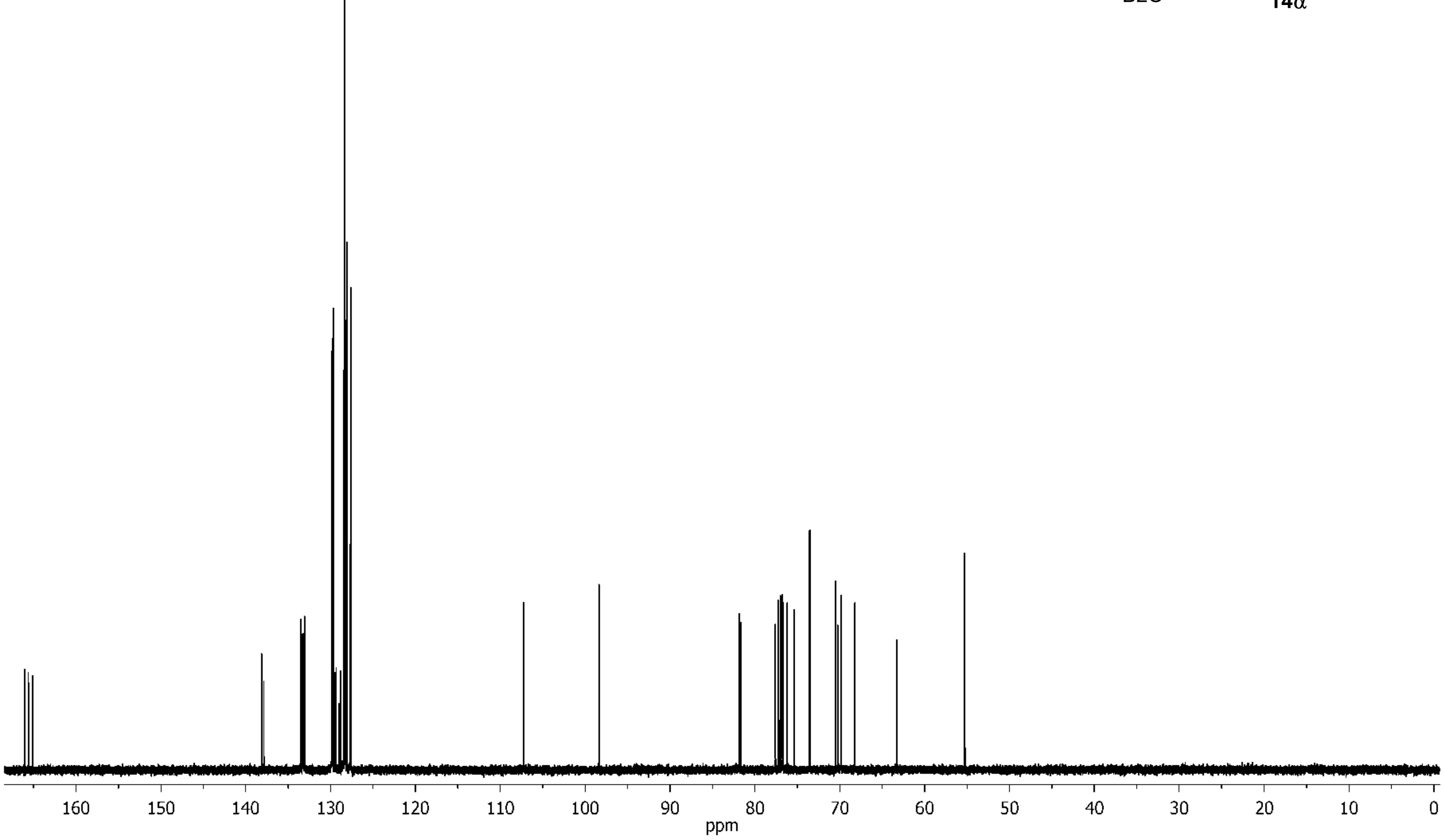
166.04
165.64
165.54
165.12
138.12
137.88
133.53
133.30
133.22
133.08
129.91
129.83
129.79
129.67
129.45
129.34
128.87
128.79
128.50
128.35
128.29
128.08
127.74
127.64
127.57
107.28
98.38
81.86
81.67
77.60
77.00
76.17
75.38
73.55
73.50
70.55
70.26
69.88
68.22
63.30
55.30
160
150
140
130
120
110
100
90
80
70
60
50
40
30
20
10
0
ppm

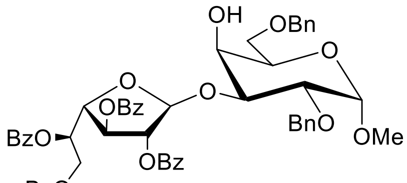
OH
OBn
O
O
O
OBz
BzO
OBz
BzO
BnO
OMe

14α

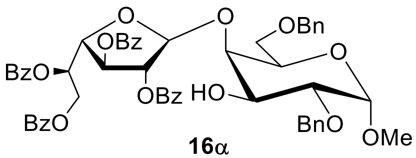
BzO
BzO
OBz
OBz
OBn
HO
BnO
OMe

16α

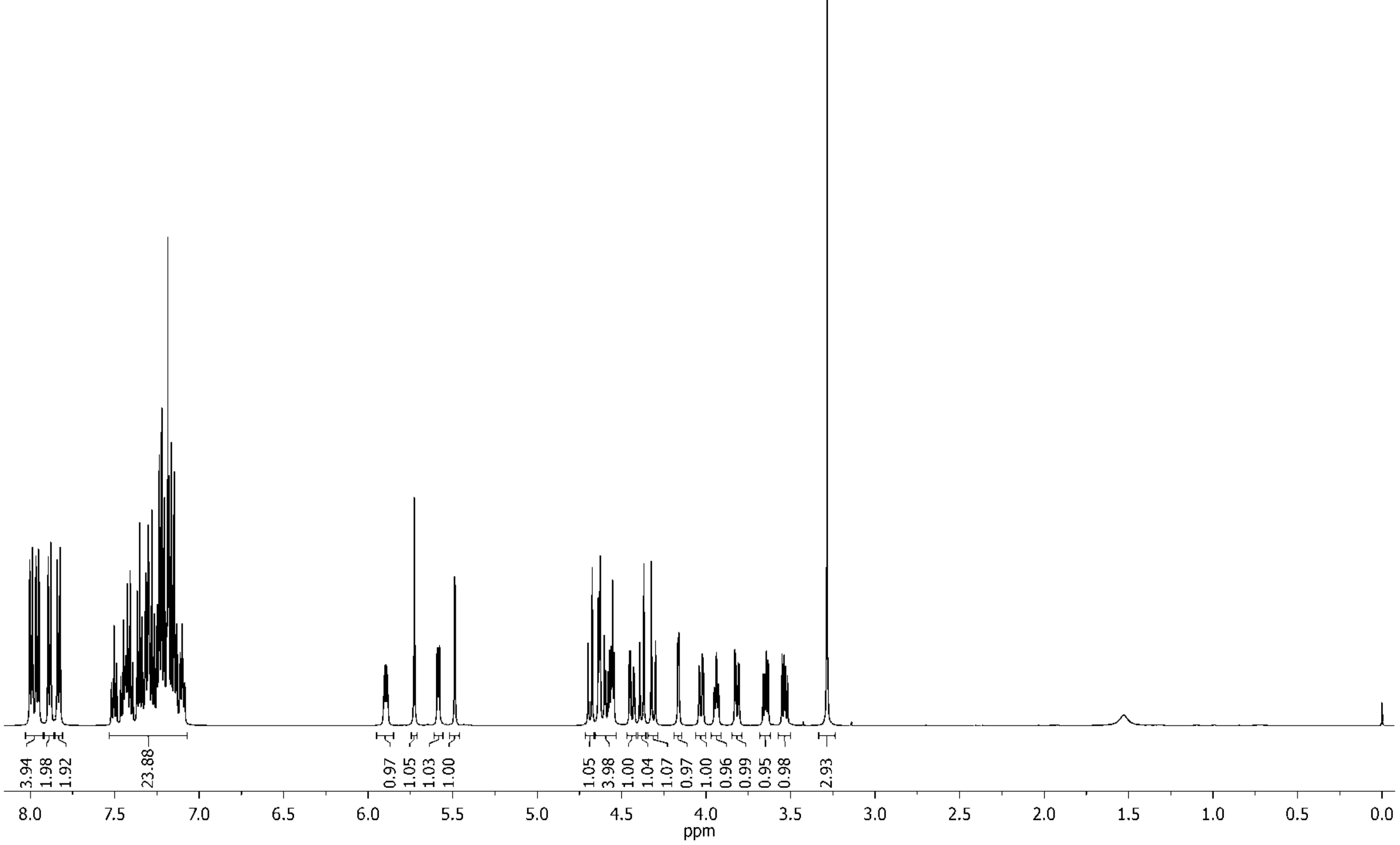
3.94
1.98
1.92
23.88
0.97
1.05
1.03
1.00
1.05
3.98
1.00
1.04
1.07
0.97
1.00
0.96
0.99
0.95
0.98
2.93
8.0 7.5 7.0 6.5 6.0 5.5 5.0 4.5 4.0 3.5 3.0 2.5 2.0 1.5 1.0 0.5 0.0
ppm

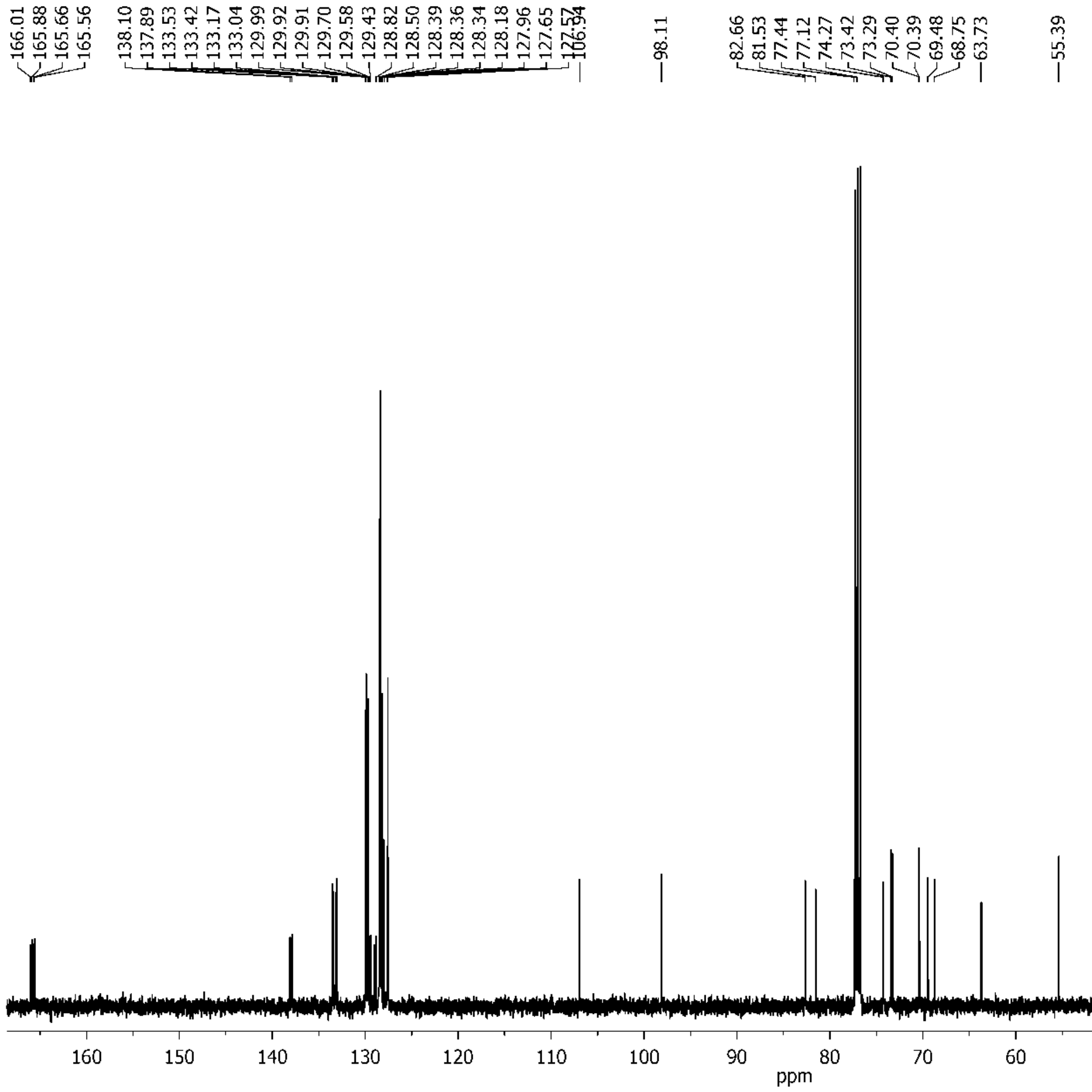
166.01
165.88
165.66
165.56
138.10
137.89
133.53
133.42
133.17
133.04
129.99
129.92
129.91
129.70
129.58
129.43
128.82
128.50
128.39
128.36
128.34
128.18
127.96
127.65
127.57
106.94
98.11
82.66
81.53
77.44
77.12
74.27
73.42
73.29
70.40
70.39
69.48
68.75
63.73
55.39
160
150
140
130
120
110
100
90
80
70
60
50
40
30
20
10
0
ppm

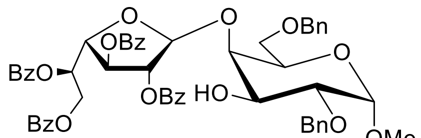
BzO
BzO
OBz
OBz
O
O
OBn
O
HO
BnO
OMe

16α

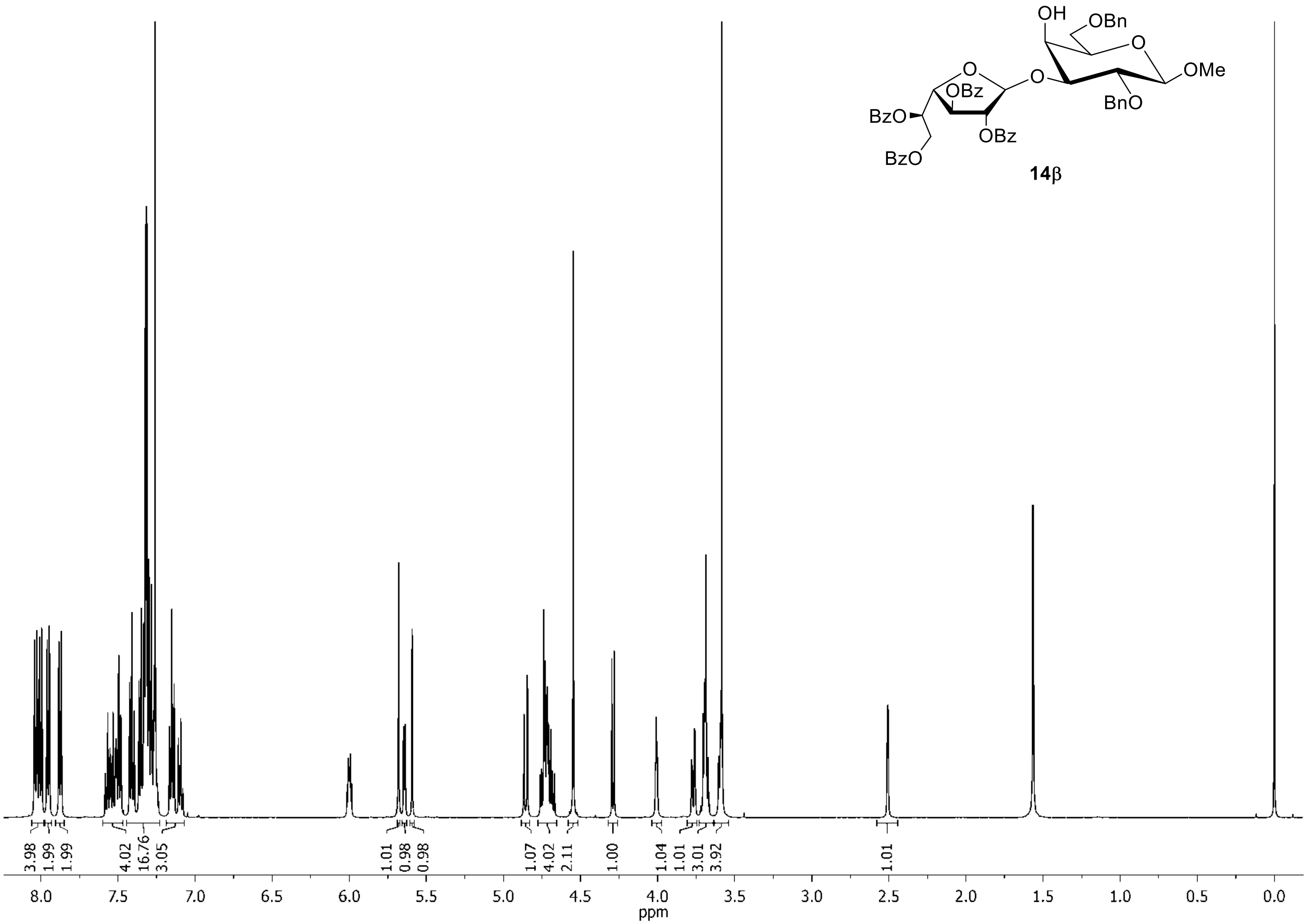
OH
OBn
O
OMe
BnO
O
OBz
BzO
OBz
BzO
3.98
1.99
1.99
4.02
16.76
3.05
1.01
0.98
0.98
1.07
4.02
2.11
1.00
1.04
1.01
3.01
3.92
1.01
8.0
7.5
7.0
6.5
6.0
5.5
5.0
4.5
4.0
3.5
3.0
2.5
2.0
1.5
1.0
0.5
0.0
ppm

14β

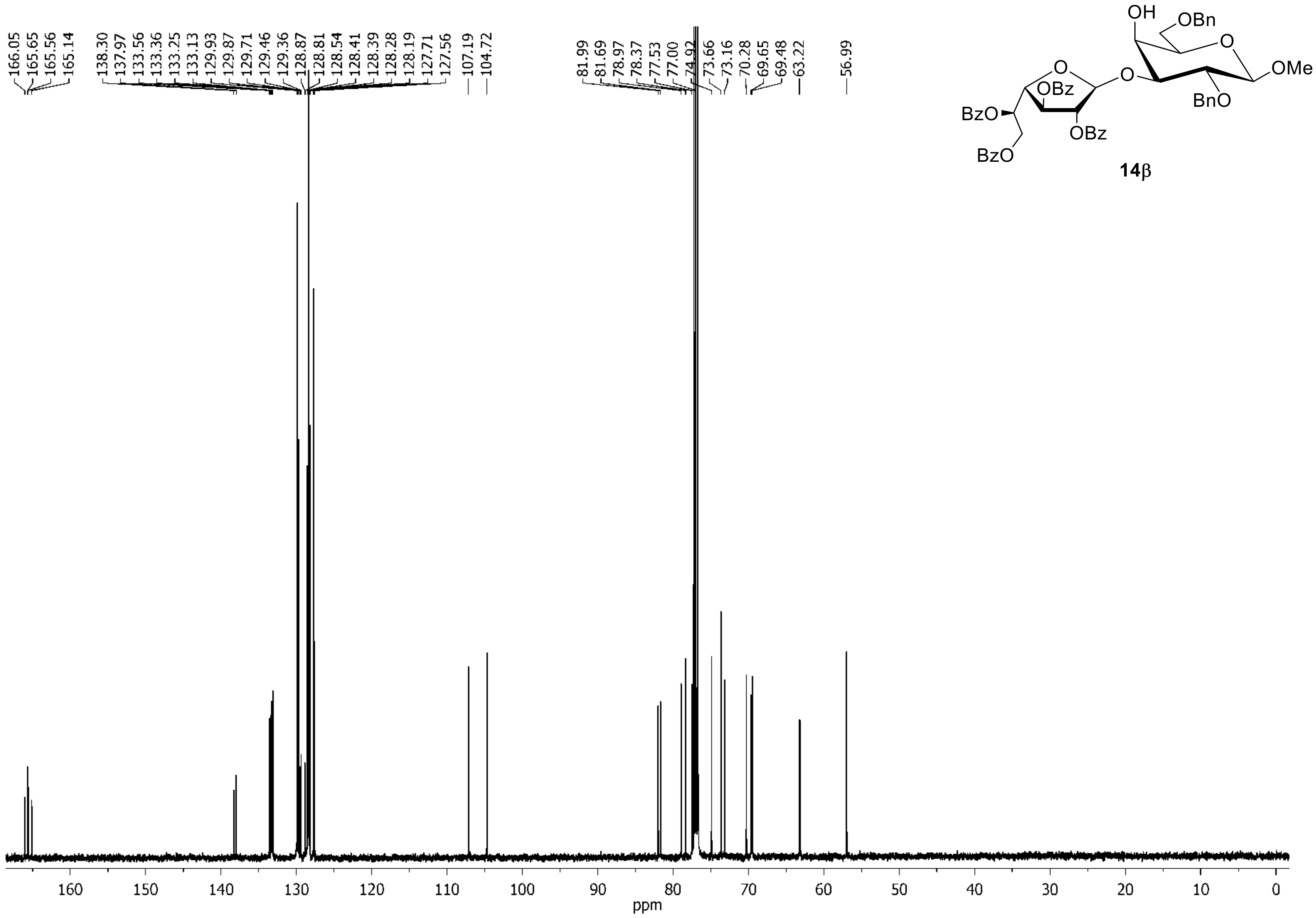
166.05
165.65
165.56
165.14
138.30
137.97
133.56
133.36
133.25
133.13
129.93
129.87
129.71
129.46
129.36
128.87
128.81
128.54
128.41
128.39
128.28
128.19
127.71
127.56
107.19
104.72
81.99
81.69
78.97
78.37
77.53
77.00
74.92
73.66
73.16
70.28
69.65
69.48
63.22
56.99
160
150
140
130
120
110
100
90
80
70
60
50
40
30
20
10
0
ppm
OH
OBn
O
OMe
O
O
OBz
BzO
BnO
OBz
BzO

14β

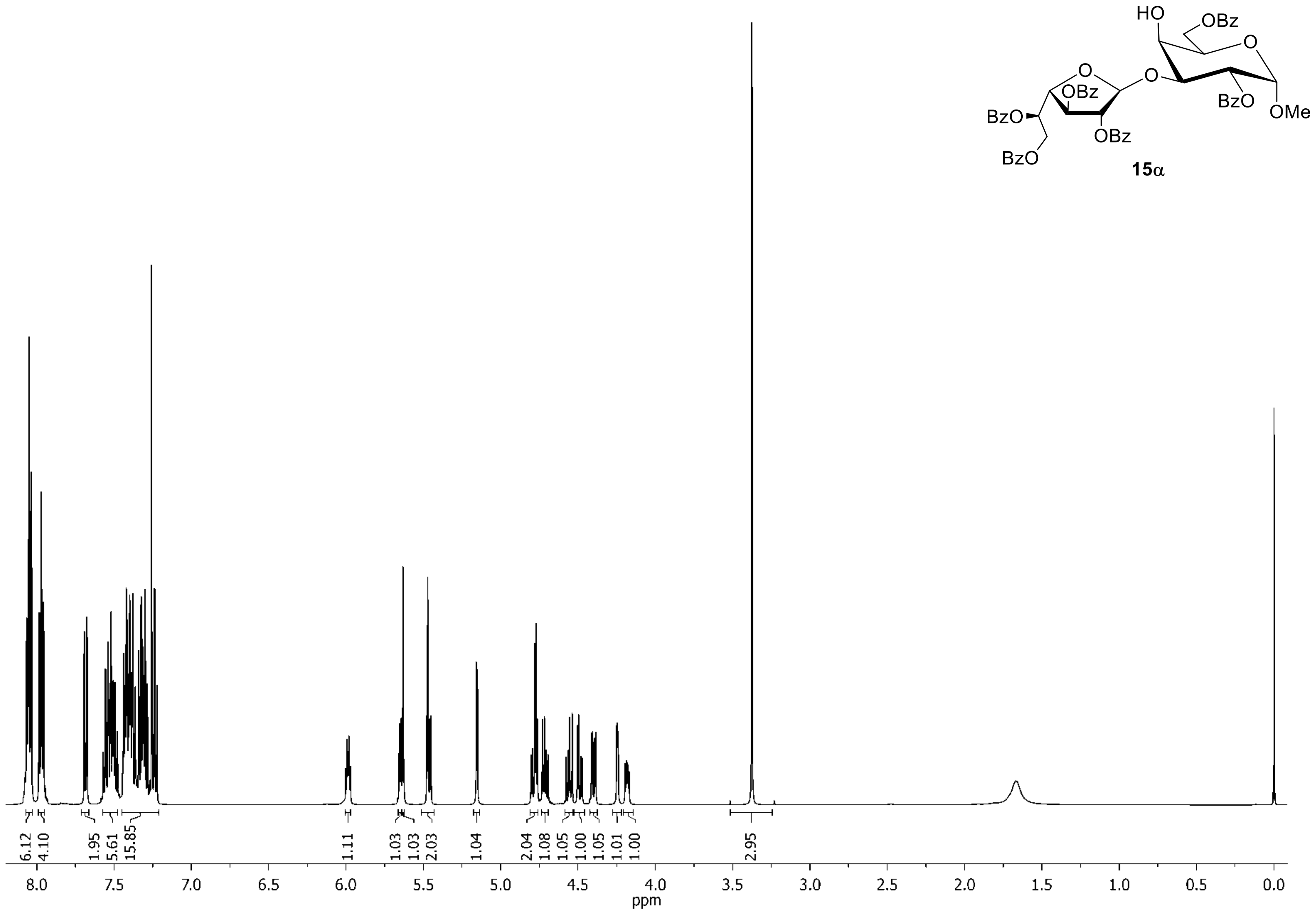
HO
OBz
O
O
O
OBz
BzO
BzO
OBz
BzO
OMe
15α
6.12
4.10
1.95
5.61
15.85
1.11
1.03
1.03
2.03
1.04
2.04
1.08
1.05
1.00
1.05
1.01
1.00
2.95
8.0
7.5
7.0
6.5
6.0
5.5
5.0
4.5
4.0
3.5
3.0
2.5
2.0
1.5
1.0
0.5
0.0
ppm

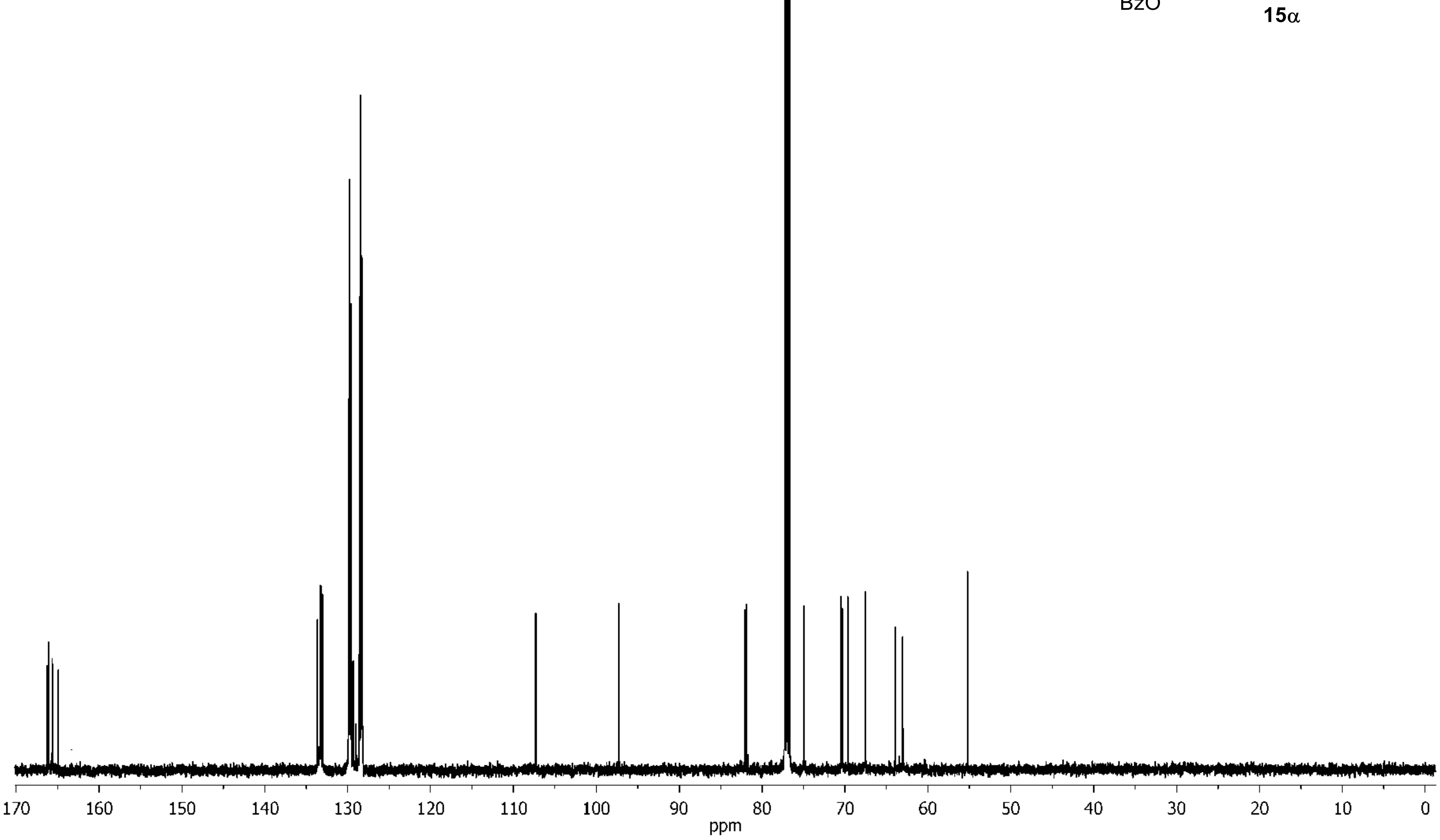
166.29
166.10
166.09
165.66
165.56
164.93
133.64
133.35
133.29
133.25
133.09
133.01
129.92
129.83
129.72
129.70
129.59
129.37
129.31
128.62
128.55
128.43
128.41
128.34
128.27
107.33
97.29
82.11
81.91
77.08
77.00
74.99
70.46
70.35
69.71
67.56
64.00
63.05
55.26
170 160 150 140 130 120 110 100 90 80 70 60 50 40 30 20 10 0
ppm

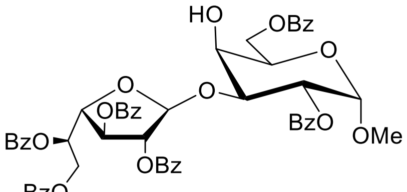
HO
OBz
O
O
O
OBz
BzO
BzO
OMe
OBz
BzO

15α

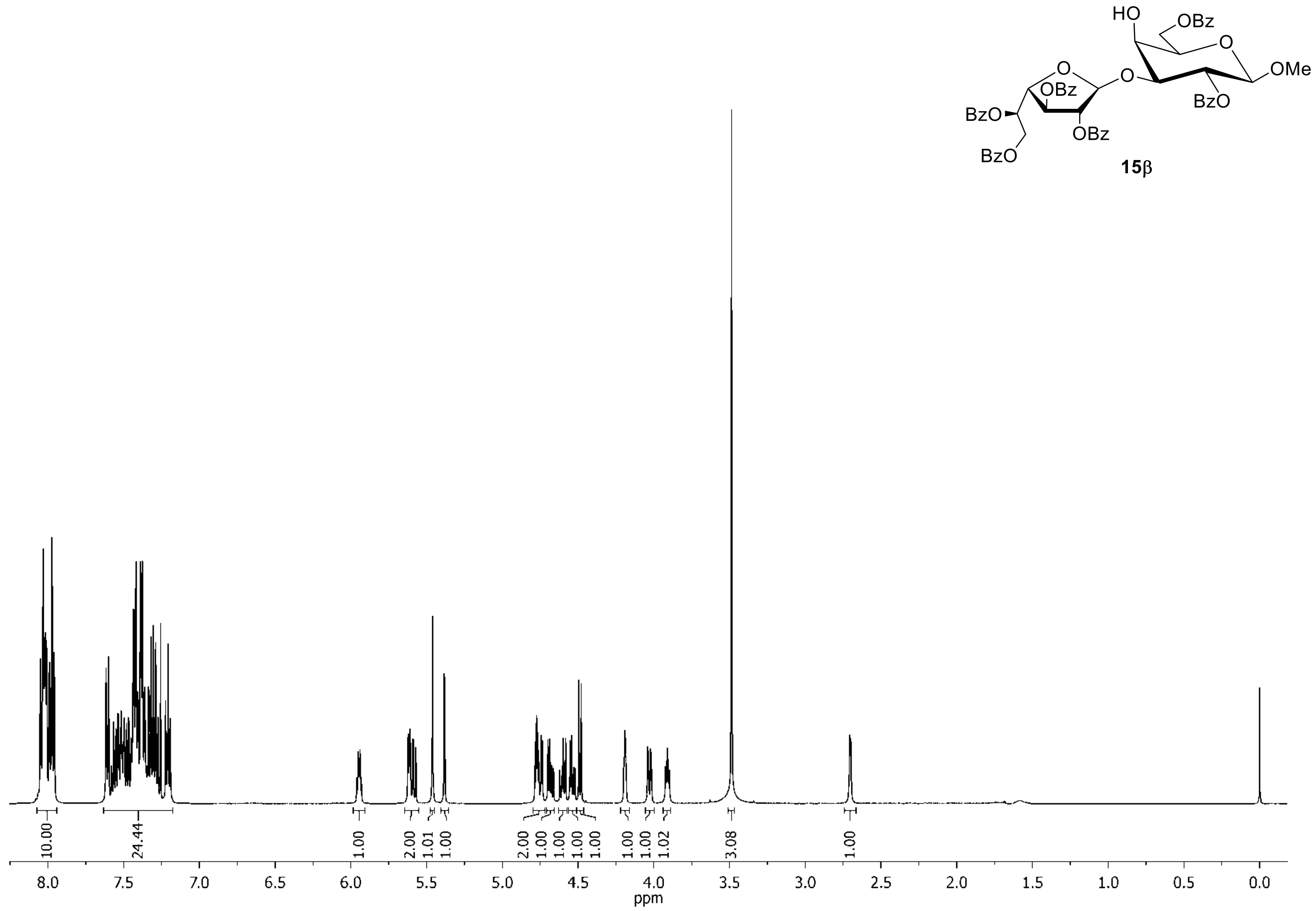
HO
OBz
O
O
OMe
O
OBz
BzO
BzO
OBz
BzO
10.00
24.44
1.00
2.00
1.01
1.00
2.00
1.00
1.00
1.00
1.00
1.00
1.00
1.02
3.08
1.00
8.0
7.5
7.0
6.5
6.0
5.5
5.0
4.5
4.0
3.5
3.0
2.5
2.0
1.5
1.0
0.5
0.0
ppm

15β

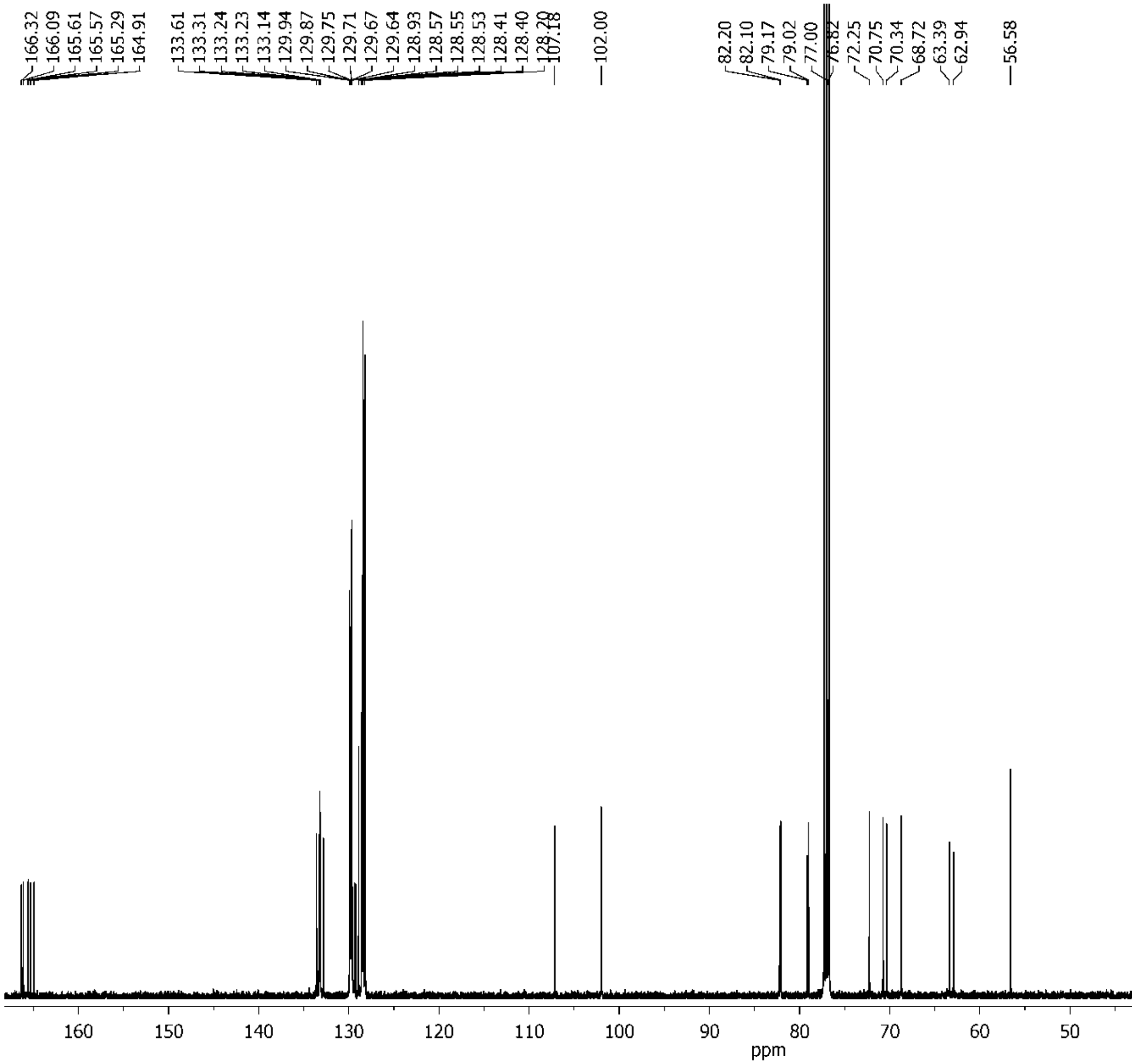
166.32
166.09
165.61
165.57
165.29
164.91
133.61
133.31
133.24
133.23
133.14
129.94
129.87
129.75
129.71
129.67
129.64
128.93
128.57
128.55
128.53
128.41
128.40
128.20
107.18
102.00
82.20
82.10
79.17
79.02
77.00
76.82
72.25
70.75
70.34
68.72
63.39
62.94
56.58
160
150
140
130
120
110
100
90
80
70
60
50
40
30
20
10
0
ppm

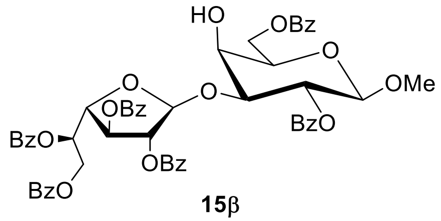
HO
OBz
O
OMe
O
O
OBz
BzO
BzO
OBz
BzO

15β

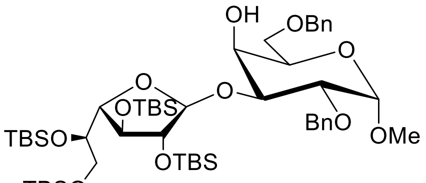
OH
OBn
O
O
OTBS
TBSO
OTBS
TBSO
BnO
OMe

18α

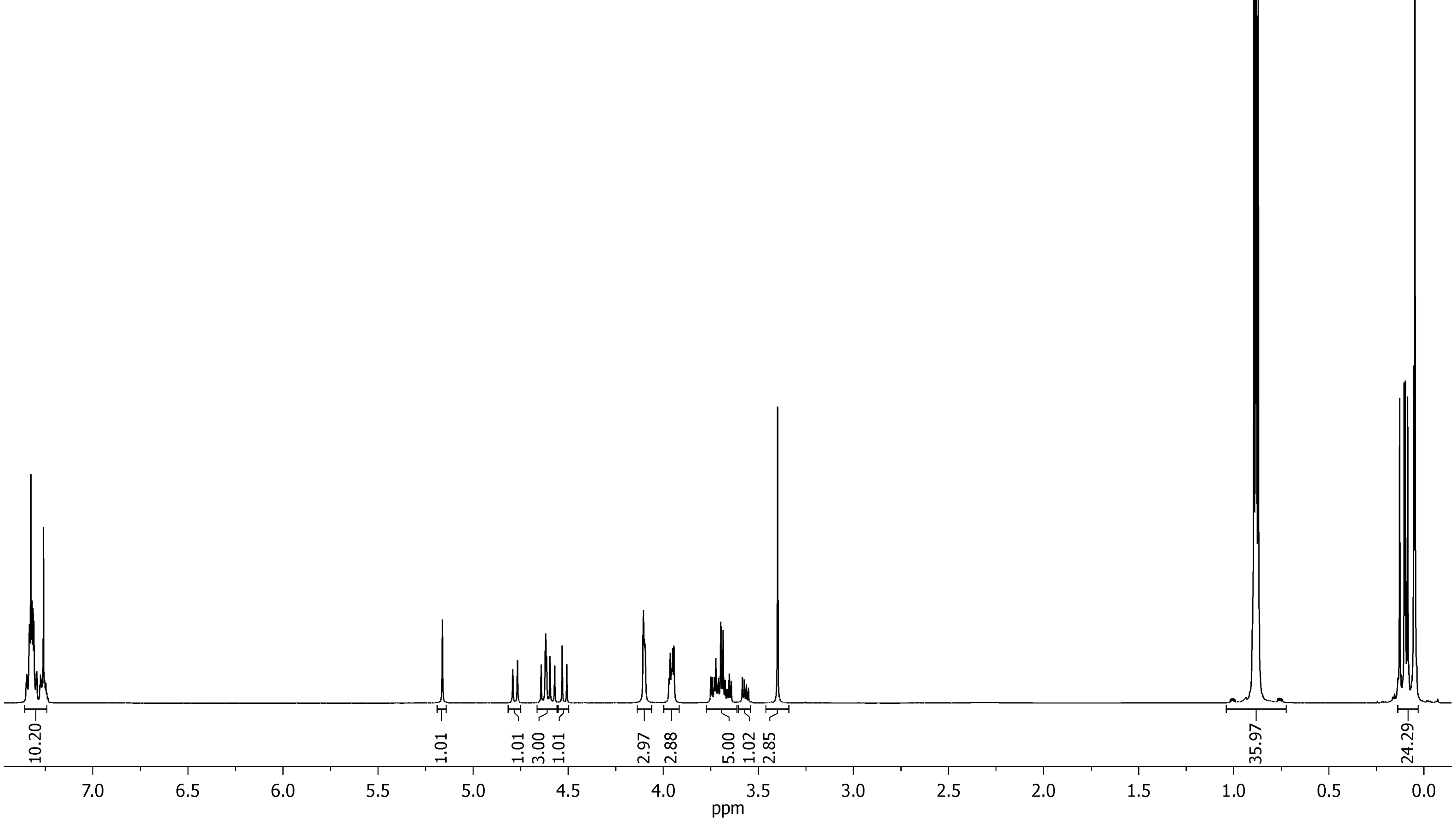
10.20
1.01
1.01
3.00
1.01
2.97
2.88
5.00
1.02
2.85
35.97
24.29
7.0
6.5
6.0
5.5
5.0
4.5
4.0
3.5
3.0
2.5
2.0
1.5
1.0
0.5
0.0
ppm

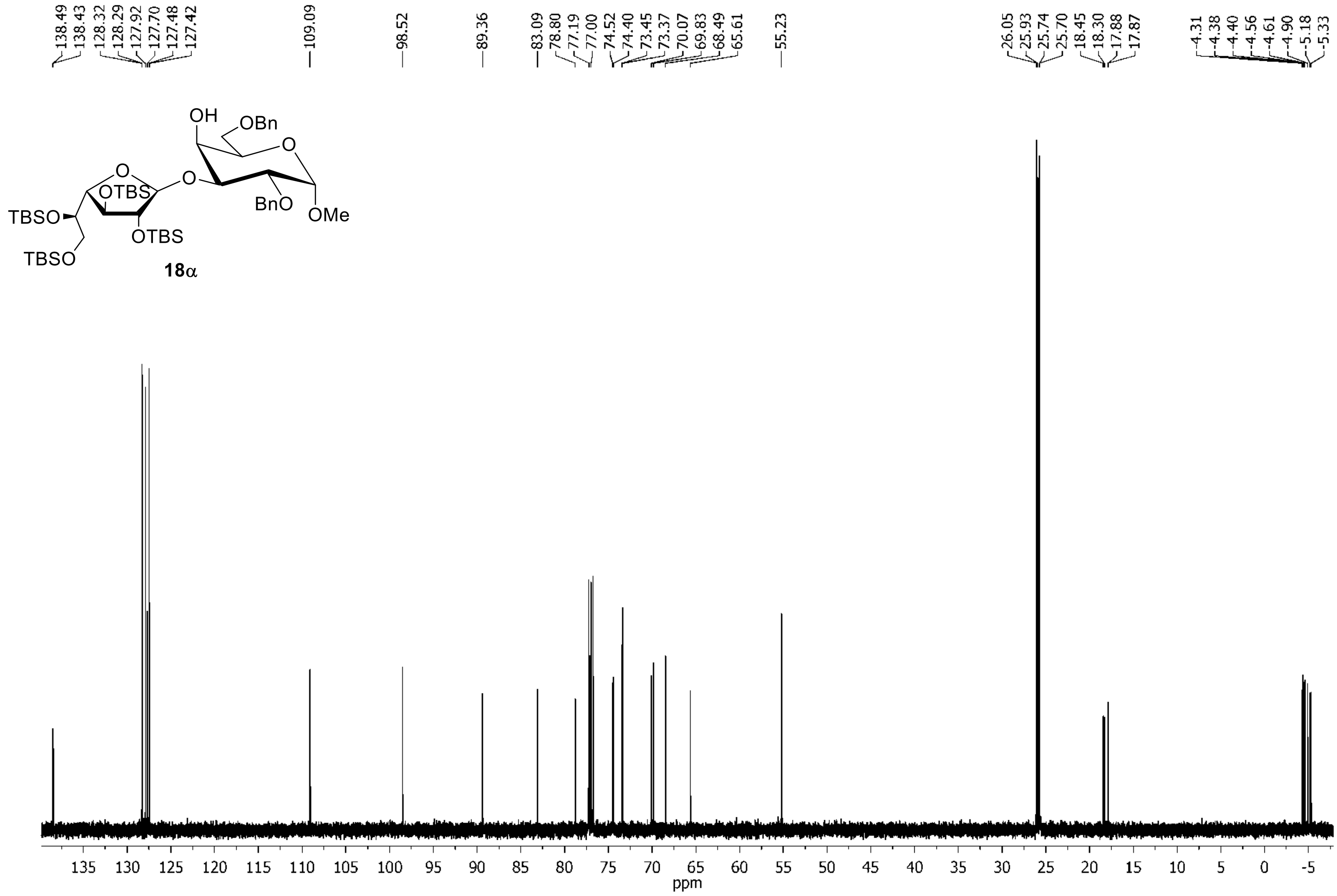
138.49
138.43
128.32
128.29
127.92
127.70
127.48
127.42
109.09
98.52
89.36
83.09
78.80
77.19
77.00
74.52
74.40
73.45
73.37
70.07
69.83
68.49
65.61
55.23
26.05
25.93
25.74
25.70
18.45
18.30
17.88
17.87
-4.31
-4.38
-4.40
-4.56
-4.61
-4.90
-5.18
-5.33
OH
OBn
O
O
OTBS
O
TBSO
OTBS
BnO
OMe
TBSO
18α
ppm

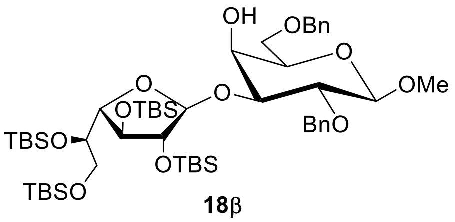
OH
OBn
O
OMe
O
O
OTBS
TBSO
OTBS
BnO
TBSO

18β

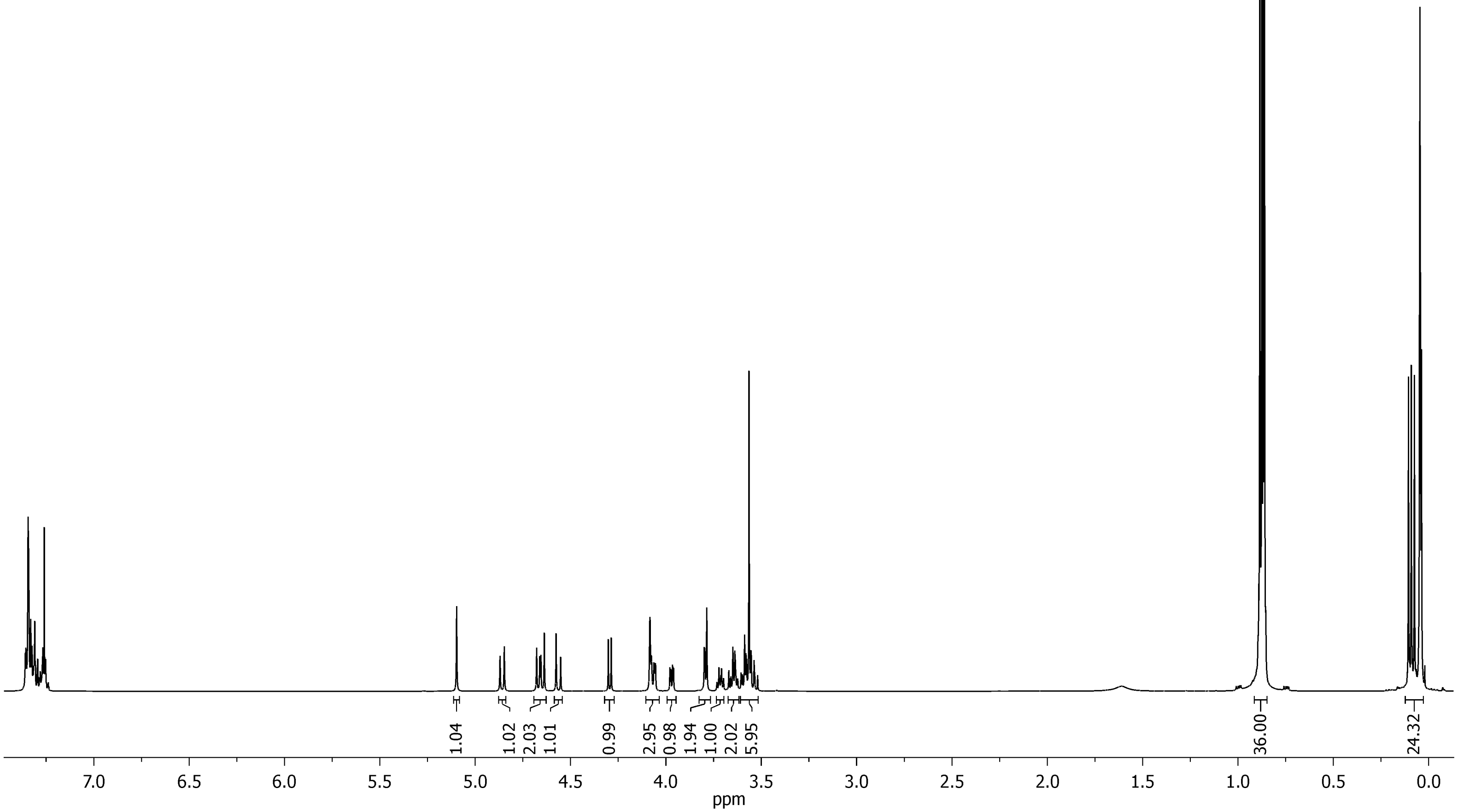
1.04
1.02
2.03
1.01
0.99
2.95
0.98
1.94
1.00
2.02
5.95
36.00
24.32
7.0
6.5
6.0
5.5
5.0
4.5
4.0
3.5
3.0
2.5
2.0
1.5
1.0
0.5
0.0
ppm

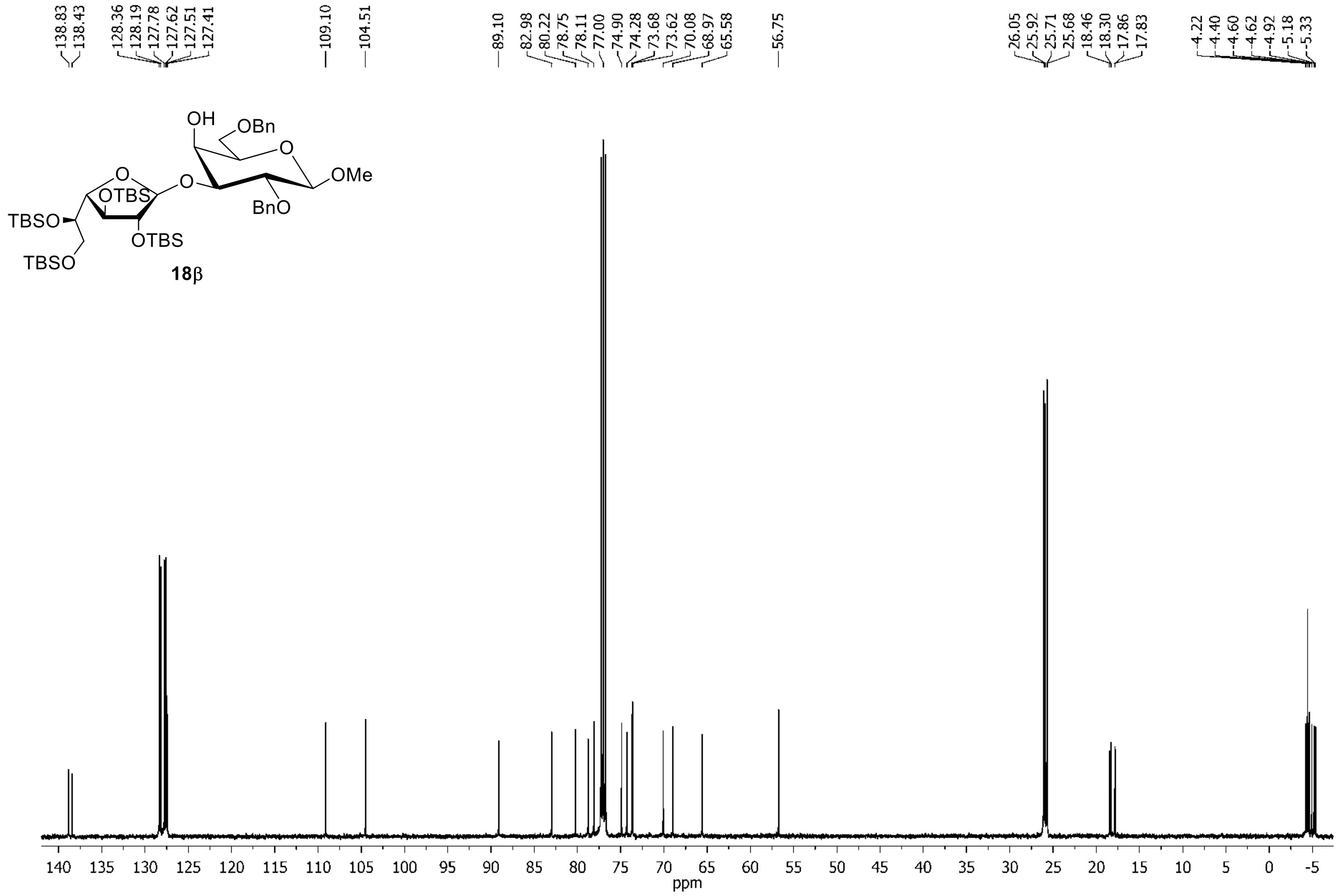
138.83
138.43
128.36
128.19
127.78
127.62
127.51
127.41
109.10
104.51
89.10
82.98
80.22
78.75
78.11
77.00
74.90
74.28
73.68
73.62
70.08
68.97
65.58
56.75
26.05
25.92
25.71
25.68
18.46
18.30
17.86
17.83
-4.22
-4.40
-4.60
-4.62
-4.92
-5.18
-5.33
OH
OBn
O
O
OMe
OTBS
TBSO
BnO
OTBS
TBSO
18β
ppm

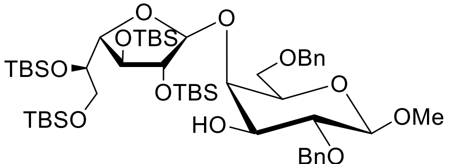
TBSO
TBSO
OTBS
OTBS
O
O
OBn
O
HO
BnO
OMe

19β

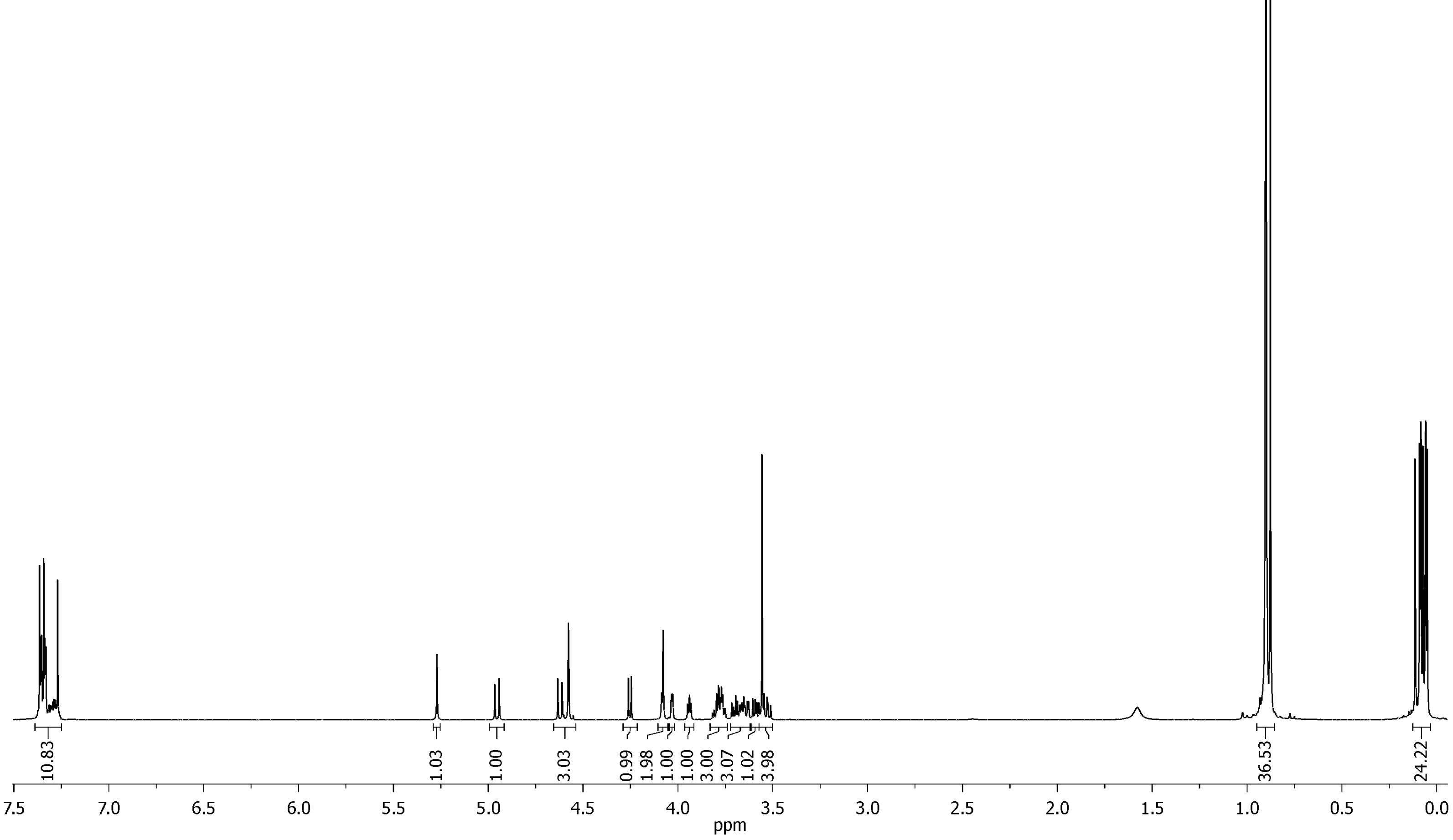
10.83
1.03
1.00
3.03
0.99
1.98
1.00
1.00
3.00
3.07
1.02
3.98
36.53
24.22
7.5 7.0 6.5 6.0 5.5 5.0 4.5 4.0 3.5 3.0 2.5 2.0 1.5 1.0 0.5 0.0
ppm

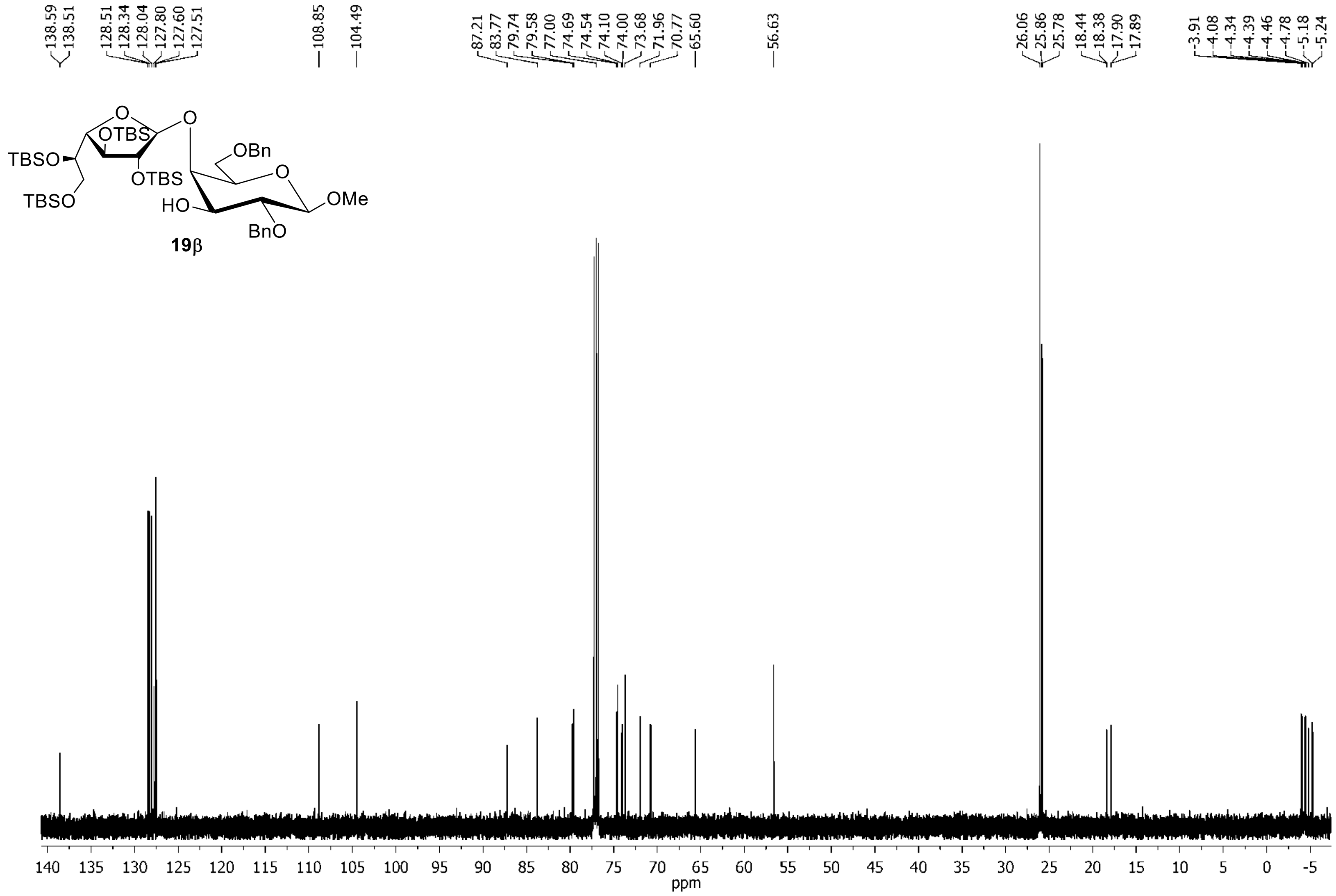
138.59
138.51
128.51
128.34
128.04
127.80
127.60
127.51
108.85
104.49
87.21
83.77
79.74
79.58
77.00
74.69
74.54
74.10
74.00
73.68
71.96
70.77
65.60
56.63
26.06
25.86
25.78
18.44
18.38
17.90
17.89
-3.91
-4.08
-4.34
-4.39
-4.46
-4.78
-5.18
-5.24
TBSO
TBSO
OTBS
OTBS
O
O
OBn
O
HO
OMe
BnO
19β
140 135 130 125 120 115 110 105 100 95 90 85 80 75 70 65 60 55 50 45 40 35 30 25 20 15 10 5 0 -5
ppm
